# Hypoxia-induced ATF3 escalates breast cancer invasion by increasing collagen deposition via P4HA1

**DOI:** 10.1038/s41419-025-07461-y

**Published:** 2025-02-27

**Authors:** Shruti Ganesh Dhamdhere, Anamika Bansal, Pranjal Singh, Parik Kakani, Shruti Agrawal, Atul Samaiya, Sanjeev Shukla

**Affiliations:** 1 Department of Biological Sciences, Indian Institute of Science Education and Research Bhopal, Bhopal, Madhya Pradesh 462066 India; 2 Department of Pathology, Bansal Hospital, Bhopal, Madhya Pradesh 462016 India; 3 Department of Surgical Oncology, Bansal Hospital, Bhopal, Madhya Pradesh 462016 India

**Keywords:** DNA methylation, Breast cancer

## Abstract

Activating transcription factors (ATFs), members of the adaptive-response gene family, participate in cellular processes to aid adaptations in response to extra and/or intracellular changes. In this study, we observed that one of the ATFs, Activating transcription factor 3 (ATF3), is upregulated under hypoxia via alterations in the epigenetic landscape of its promoter, followed by transcriptional upregulation. Under hypoxic conditions, Hypoxia-inducible factor 1-alpha (HIF1ɑ) alleviates methylation at the *ATF3* promoter by recruiting TET1 and induces *ATF3* transcription. In addition, our RNA-seq analysis showed that ATF3 globally affects transcription under hypoxia and controls the processes of EMT and cancer invasion by stimulating the transcription of Prolyl 4-Hydroxylase Subunit Alpha 1 (P4HA1), an enzyme which enhances invasion-conducive extracellular matrix (ECM) under hypoxic conditions. Prolyl hydroxylases play a critical role in the hydroxylation and deposition of collagen in the extracellular matrix (ECM) during the evolution of cancer, which is necessary for metastasis. Importantly, *P4HA1* undergoes alternative splicing under hypoxia, where the inclusion of exon 9a is increased. Interestingly, involvement of ATF3 in *P4HA1* splicing was also evident, as binding of ATF3 at intron 9a led to demethylation of this DNA region via recruitment of TET1. Furthermore, we also show that the demethylated DNA region of intron 9a then becomes accessible to CCCTC-binding factor (CTCF). Thus, a cascade of demethylation via ATF3 recruited TET1, followed by increased RNA Pol II pause at intron 9a via CTCF, leads to inclusion of exon 9a. The *P4HA1 9a* isoform leads to enhanced invasion under hypoxic conditions by increasing deposition of collagen in the ECM. These results reveal a novel hypoxia-induced HIF1ɑ-ATF3-P4HA1 axis which can potentially be exploited as a therapeutic target to impede EMT and ultimately breast cancer invasion.

**Figure:**
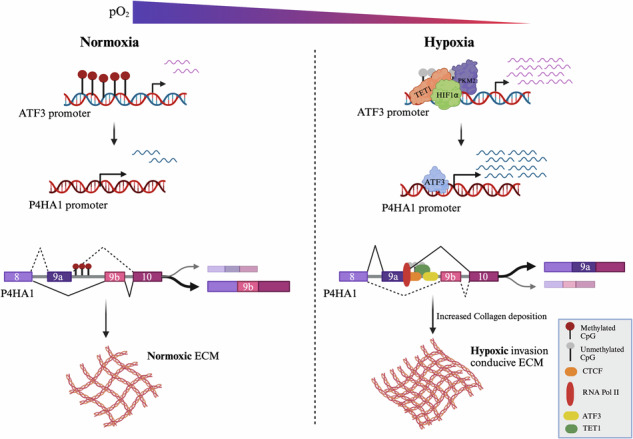
Hypoxia induced ATF3 regulates P4HA1 expression and alternative splicing to promote breast cancer invasion.

Hypoxia induced ATF3 regulates P4HA1 expression and alternative splicing to promote breast cancer invasion.

## Introduction

The tumor microenvironment (TME) is a dynamic ecosystem, which while evolving is accompanied by various physico-chemical stressors. As the tumor progresses, altered metabolism, and aberrant vasculature leads to generation of hypoxic niches [1, 2]. Hypoxia is a well-known orchestrator of genetic [3, 4] and epigenetic alterations [5, 6] and is considered to be an important hallmark of the TME that promotes epithelial to mesenchymal transition (EMT) [7, 8], cancer invasion [9], stemness [10] and drug resistance [11]. Hypoxia-inducible factor 1-alpha (HIF1α), the master transcription factor of hypoxia, empirically facilitates the transcription of major genes in response to the hypoxic stress [12]. However, there could be other genes that are involved in the adaptation of cancer cells and are indispensable in a hypoxic environment.

The adaptative response genes are the early response genes upregulated in response to various stress conditions, such as acidic stress, oxidative stress, osmotic stress, mechanical stress, and hypoxic stress [13–18]. These genes belong to the ATF/CREB family of transcription factors, which consists of almost 20 members [14]. The ATF/CREB family of transcription factors are DNA binding factors with a distinct basic region-leucine zipper (bZip) DNA binding domain. The basic region endows the members of this family with the ability to bind to DNA, whereas the bZip region allows them to form dimers with themselves or other family members, leading to differential DNA binding ability [14]. Among the ATF/CREB family members, ATF3 is a well-researched adaptive response gene that functions in cellular processes to support adaptations in response to extracellular and/or intracellular alterations [13]. Numerous reports suggest that ATF3 is an oncogene that enhances the cell motility and invasion potential by upregulating genes involved in metastasis, such as Snail, Slug, TWIST1, plasminogen activator inhibitor-1, caveolin-1, MMP13. In addition, ATF3 contributes to EMT by upregulating genes such as vimentin, fibronectin, and N-cadherin [19–21].

In this study, we elucidated the cascade of events that lead to hypoxia-mediated ATF3 induction and the associated epigenetic alterations. We observed a HIF1α-dependent recruitment of TET1 to the *ATF3* promoter, which led to demethylation of the promoter and induced ATF3 expression. Furthermore, we delineated the regulatory mechanism of expression of the collagen organizing enzyme P4HA1 by ATF3. P4HA1 is a crucial enzyme that hydroxylates proline residues of newly synthesized collagen to pack them into triple helical structures, which are subsequently deposited in the extracellular matrix (ECM) [22]. *P4HA1* undergoes a mutually exclusive alternative splicing event [23, 24], during which either exon 9a or 9b is included in the transcript. Interestingly, we found that ATF3 also plays an intricate role in P4HA1 splicing, as it is essential for demethylation and recruitment of a very well-known mediator of alternative splicing CTCF (CCCTC binding factor) [25–27] at intron 9a under hypoxia. Furthermore, CTCF mediates RNA Pol II pause at intron 9a, leading to the inclusion of exon 9a in the *P4HA1* transcript. The *P4HA1 9a* isoform renders enhanced invasive potential to cancer cells as it augments invasion conducive ECM by increasing deposition of collagen. Thus, our study unveils the HIF1α-ATF3-P4HA1 axis and its influence over enhancement of invasive potential in hypoxic breast cancer cells.

## Materials and methods

### Cell culture and hypoxia treatment

Human breast cancer cell lines MCF7 (ATCC HTB22) [28] and HCC1806 (ATCC CRL2335) [29] obtained from American Type Culture Collection (ATCC) were cultured in DMEM (Invitrogen, 12800017) and RPMI (Invitrogen, 23400021), respectively, HEK293T (ATCC CRL1573) was cultured in DMEM, Primary breast cell lines were cultured in DMEM/F12(1:1). Cell lines generated using CRISPR/Cas9 systems were also cultured in DMEM. The culture media for all the cell lines was supplemented with 10% fetal bovine serum (Sigma, F7524), 100 units/ml of penicillin and streptomycin (Invitrogen, 15140122) and 2 mmol/l L-glutamine (Invitrogen, 25030081). All cells were cultured at 37 °C with 5% CO_2_. Cells were treated under hypoxic conditions in Ruskinn INVIVO2 400 hypoxia chamber at 37 °C with 1% O_2_, 5% CO_2_. All the cell lines were authenticated from a national cell repository facility by short tandem repeats (STR) profiling and were routinely tested for mycoplasma contamination.

### Plasmids

The ATF3 overexpression construct was generated by cloning the *ATF3* CDS insert between *BamHI* and *XhoI* sites of the pCMV-3Tag-1A (Agilent, 240195) mammalian expression vector. For construction of overexpression vectors for *P4HA1 9a* and *9b* isoforms the *P4HA1* CDS insert was cloned between *BamHI* and *XhoI* sites of the pEGFP-C3 (Clontech, 6082-1) mammalian expression vector. The clones positive for *P4HA1* inserts were then used for screening for identifying the *9a* and *9b* isoforms. A fragment containing exons 8 to 10 was amplified and subjected to *BsmbI* digestion. The clones were identified as be 9a or 9b isoform on the basis that only the 9a isoform contained the *BsmbI* site. The primers used for generating the inserts and for screening of *P4HA1 9a* and *9b* isoforms are listed Supplementary Table S1.

### RNA isolation and cDNA synthesis

TRIzol (Invitrogen, 15596026) was used to extract total mRNA. The RNA concentrations were measured on Eppendorf BioSpectrometer. PrimeScript 1st strand cDNA Synthesis Kit (TaKaRa, 6110A) was used to convert the RNA into cDNA. RNA isolation and cDNA synthesis was done by as per manufacturer’s instructions.

### Quantitative RT-PCR

Amplification reactions were performed on using KAPA SYBR FAST (Sigma, KK4618) in qTOWER3 Touch Real Time PCR (analytikjena). The gene/exon expression were calculated using the following formula: 2^(Ct_control − Ct_target)^, where the housekeeping gene *RPS16* was used as control for normalization. In case of *P4HA1* splicing, a ratio of 9a/9b was used to study the inclusion of their respective exons. All the primers used for qPCR are listed in Supplementary Table S1.

### Western blotting

The cells were lysed using urea lysis buffer (8 M urea, 2 M thiourea, 2% CHAPS, 1% DTT) supplemented with 1× Protease inhibitor cocktail (0.5 mM Leupeptin, 250 μM Pepstatin, 50 mM EDTA) and 100 mM PMSF. For lysis, the cells were incubated on a rocker at 4 °C for 30 min followed by centrifugation at a speed of 14,000 × *g* at 4 °C. The supernatant was collected in a fresh tube and quantified using Bradford assay. Equal amounts of protein was used for all experiments. The proteins separated by SDS-PAGE were electro-transferred onto an activated PVDF membrane and blocked with skimmed milk. The blots were incubated with recommended dilutions of primary antibodies overnight at 4 °C or for 1 h at room temperature followed by secondary antibody incubation for 1 h at room temperature. The blots were scanned on Odyssey membrane scanning system and quantified using ImageJ software. The details for all antibodies used are provided in Supplementary Table S2. The original uncropped western blots are provided as a separate Supplementary file 2.

### Luciferase reporter assay

Fragments of ATF3 and P4HA1 promoters were amplified using MCF7 genomic DNA as template and cloned between *KpnI* and *XhoI* sites of the pGL3 basic luciferase reporter vector (Promega, E1751). Details about primers used to amplify them have been provided in Supplementary Table S1. Cells were seeded in 24-well plate for performing Luciferase assay. The promoter-cloned Luciferase vectors were co-transfected with pRL-TK Renilla Luciferase plasmid (Promega, E2231). After 12 h the cells were supplemented with fresh media and subjected to hypoxic condition. After treatment the cells were lysed with passive lysis buffer and the Firefly and Renilla Luciferase activity was measured using substrate buffers containing D-Luciferin and Coelenterazine, respectively, on Cytation Multiplate reader (Agilent Biotek). The relative luciferase activity of the promoter fragments were calculated by normalizing the Firefly luciferase activity values with Renilla luciferase activity values and the empty pGL3 basic luciferase plasmid.

### Site-directed mutagenesis

The HREs and the ATF3 binding sites on the respective *ATF3* and *P4HA1* promoters were mutated using site-directed mutagenesis. Wild-type luciferase reporter vectors (−553 fragment of *ATF3* promoter and −306 fragment of *P4HA1* promoter) were used as templates to mutate the HREs and ATF3 binding sites and construct the SDM luciferase reporter plasmids using oligonucleotides with mutated binding site sequences. The HRE sequences CGTG at 317, 334, 488, 492 bp upstream of the TSS at the *ATF3* promoter were mutated into TTTT or AAAA. Similarly, the ATF3 binding site at 248 bp upstream of the TSS at the *P4HA1* promoter was mutated from CGTTACAT to TTTTATGT. These constructs were used to perform Luciferase assay as mentioned for the wild-type constructs. Details of the SDM luciferase reporter constructs and primer have been provided in Supplementary Table S1. All the mutations were confirmed by DNA sequencing.

### Breast cancer sample collection and immunohistochemistry

Breast tumor tissue sections embedded in paraffin and fixed on poly-L-lysine coated slides were collected from Bansal Hospital, Bhopal, India. All the tumor samples were utilized post obtaining informed consent from the breast cancer patients. This study was performed after approval from the Institute Ethics Committee of the Indian Institute of Science Education and Research Bhopal, India (IISERB/IEC/Certificate/2021-I/08). The sections were deparaffinized overnight at 65 °C, treated with xylene and rehydrated using gradient concentrations of ethanol (100%, 95%, 70%, 50%). Antigen retrieval was done by boiling the tissue in citrate buffer (pH 6.0) for 13 min. To quench the endogenous peroxidase the tissues were treated with 10% hydrogen peroxide solution in ice cold methanol for 45 min at room temperature. The tissues were then blocked using 5% BSA for 1 h at room temperature. Primary antibodies against CAIX and ATF3 were incubated overnight at 4 °C in separate sections of the same sample. The next day, DAB staining was performed according to the manufacturer’s instructions using the Biogenex super sensitive™ polymer HRP IHC detection kit (QD430-XAKE, lot no. QD4300919). A total of 16 patient samples were used. The hypoxic regions were identified by checking for positive CAIX staining and the same regions were checked for ATF3 staining in other section to mark the co-localization. The details of these tissues obtained from breast cancer patients are provided in Supplementary Table S3. Quantification was performed using ImageJ (Fiji) with color deconvolution, and the mean gray values of three regions (identical in the two sections) containing cancerous cells (to avoid values from any staining in the stromal cells) were then converted to optical density. Mann-Whitney test was used for statistical comparison. Original uncropped images are provided as Supplementary file 3.

### Establishment of patient-derived cell line

Tumor tissues were collected from breast cancer patients undergoing surgery at Bansal Hospital, Bhopal, India after obtaining informed consent from the patients. This study was performed after approval from the Institute Ethics Committee of the Indian Institute of Science Education and Research Bhopal, India (IISERB/IEC/Certificate/2021-I/08). The tumor samples were transported in DMEM/F12 (1:1) media supplemented with 50 μg/mL gentamycin sulfate (TCI, Cat no. G0383), 200 units/ml of penicillin and streptomycin (Invitrogen, Cat no. 15140122), 0.01% antibiotic-antimycotic solution (Invitrogen, Cat no. 15240062) and 20% CELLect fetal bovine serum, gold, US origin (MP Biomedicals, Cat no. 092916754) immediately after the surgery. Once arrived the entire procedure was performed in sterile conditions inside a laminar hood. The transport media was discarded and the tissue was placed in a petri dish with a few drops of incomplete media (1:1 DMEM/F12) and minced followed by enzymatic disaggregation with 1 mg collagenase (Sigma, Cat no, C2674-1) and 120 U hyaluronidase (TCI, Cat no. H064) for 45 min–1 h at 37 °C with rigorous pipetting at an interval of 15 min. The cells obtained after digestion were then passed through a 70 μm cell strainer (Falcon, Cat no. 352350). The single-cell suspension was then centrifuged at 350 × *g* for 5 min. The cell pellet was then resuspended in 1X TAC buffer (1:9 ratio of 170 mM Tris, pH 7.4 and 150 mM NH_4_Cl, pH 7.4) and incubated at 37 °C for 10 min to remove all RBCs. The cell suspension was again centrifuged at 350 × *g* for 5 min. The obtained pellet was resuspended in the complete media (1:1 DMEM/F12, supplemented with 50 μg/ml gentamycin sulfate, 200 U/ml penicillin and streptomycin, 0.01% antibiotic-antimycotic solution and 10% CELLect fetal bovine serum, gold, US origin (MP Biomedicals, Cat no. 092916754)). This cell line was cultured at 37 °C with 5% CO_2_ and later used for various experiments. The cells were stained by immunofluorescence for Epithelial/Mesenchymal markers, fibroblast markers for characterization. The results validated the epithelial origin of this patient derived cell line and showed that it did not contain any fibroblast population (Fig. S1I).

### RNA interference

HEK293T cells were used to produce lentiviruses containing shRNAs against the target genes. The plasmid containing shRNA (Sigma, Mission Human Genome shRNA Library) was transfected along with the packaging plasmids into HEK293T cells and lentivirus was collected after 24 and 48 h of transfection. MCF7 and HCC1806 cells seeded at a density of 3 × 10^5^ cells per well in a six-well culture plate were inoculated with the media containing lentivirus obtained from HEK293T cells with 8 μg/ml polybrene (Sigma, H9268, lot no. SLBH5907V). After 12 h the media was changed and the cells were selected with 1 μg/ml puromycin (Sigma, P9620, lot no. 034M4008V) for 72 h before using for any subsequent experiments. The list of shRNAs and their sequences used in this study are provided in Supplementary Table S1.

### Genomic DNA isolation and methylated DNA immunoprecipitation

After treatment, the cells were processed to obtain genomic DNA using gDNA isolation kit (Sigma, G1N70). Approximately 3 μg of gDNA was sonicated to obtain a fragment size ~400–100 bp using the Bioruptor sonicator (Diagenode). The sonicated DNA was then denatured at 95 °C for 10 min and then incubated with 5-methyl cytosine antibody and a control normal rabbit IgG antibody overnight at 4 °C and 5% Input DNA was collected before adding antibodies. Next day, Dynabeads Protein G (Invitrogen, Cat No. 10004D) were added to capture the antibody-DNA complexes. The obtained complexes were de-crosslinked in TE buffer with 1% SDS and proteinase K (Invitrogen, Cat no. 25530049) overnight at 65 °C. The obtained DNA and input were then PCR purified using PCR purification kit (QIAGEN, 28106). The immunoprecipitated samples along with input were quantitatively analyzed by qPCR using KAPA SYBR FAST (Sigma, KK4618) in qTOWER3 Touch Real Time PCR (analytikjena). The enrichment was calculated using input as normalization factor and the following formula: 2^(Ct_input − Ct_Immunoprecipitation)^. In addition, the resultant values were normalized by the enrichment values of normal rabbit IgG. The final values are represented as mean ± SD of triplicates. The details of primers used for MeDIP qPCR are provided in Supplementary Table S1.

### Chromatin immunoprecipitation

After treatment, the cells were fixed using 1% formaldehyde and subsequently quenched with 0.125 M glycine. The cells were then scraped in PBS and pelleted by centrifugation to be lysed using three consecutive lysis buffers LB1 (50 mM Hepes-KOH, pH 7.5, 140 mM NaCl, 1 mM EDTA, 10% glycerol, 0.5% NP40, 0.25% Triton X-100), LB2 (10 mM Tris HCl, pH 8, 200 mM NaCl, 1 mM EDTA, 0.5 mM EGTA) and LB3 (10 mM tris HCl, pH 8, 100 mM NaCl, 1 mM EDTA, 0.5 mM EGTA, 0.1% Na-Deoxycholate, 0.5% N-lauroylsarcosine). The samples were lysed in each lysis buffer for 10 min while pipetting every 3 min and pelleted down before proceeding to the next buffer. In LB3 the sample was pipetted approximately 50 times with a 200 μl pipette and passed through a 25 gauge needle thrice. The chromatin retrieved from lysis was then subjected to sonication to obtain a fragment size ~400–100 bp. About 25 μg of the sonicated chromatin was incubated with the antibodies of interest along with 1/20th of 20% of Triton X-100 overnight at 4 °C. Control IgG antibodies were used as controls, and 5% input was collected before adding antibodies. Next day, Dynabeads Protein G (Invitrogen, Cat No. 10004D) were added to capture the antibody-Protein-chromatin complexes. The obtained complexes were eluted and treated with RNase A for 30 min at 37 °C followed by de-crosslinking in TE buffer with 1% SDS and proteinase K (Invitrogen, Cat no. 25530049) overnight at 65 °C. The obtained DNA and input was then PCR purified using PCR purification kit (QIAGEN, 28106). The immunoprecipitated samples along with input were quantitatively analyzed by qPCR using KAPA SYBR FAST (Sigma, KK4618) in qTOWER3 Touch Real Time PCR (analytikjena). The enrichment was calculated using input as normalization factor and the following formula: 2^(Ct_input − Ct_Immunoprecipitation)^. In addition, the resultant values were normalized by the enrichment values of IgG. The final values are represented as mean ± SD of triplicates. The details of primers used for ChIP qPCR are provided in Supplementary Table S1.

### Transwell invasion assay

Transwell inserts (Corning, Cat no. 3422) were coated with 100 μl Matrigel (Corning, Cat no. 356230) at a concentration of 0.25% and allowed to set for 3 h at 37 °C. Then about 3 × 10^4^ cells knocked down or overexpressed with the gene of interest and the control wild-type cells were seeded onto the Matrigel in incomplete media (without FBS) with complete media (10% FBS) in the lower chamber. These cells were then incubated in normoxic or hypoxic conditions for 48–72 h. The inserts were then washed in PBS mildly to remove the non-invasive cells and media followed by fixation with 4% formaldehyde for 10 min and staining with 0.05% crystal violet (made in 10% methanol in PBS). The inserts were then dried and imaged using an inverted microscope (Olympus CKX41). Five random fields were used to count the number of invaded cells. The percentage of invaded cells were represented as mean ± SD of triplicates.

### COL1A1 immunostaining

Cells were seeded on a coverslip and subjected to normoxia or hypoxia. At the end of the treatment the cells were fixed with 4% formaldehyde for 15 min at room temperature. Then cells were blocked with 1% BSA made in 0.03% Triton-X PBS for 1 h at room temperature followed by incubation with primary antibody of COL1A1 at the dilution of 1:100 overnight at 4 °C. The next day primary antibody was removed and the cells were washed with 0.1% PBST thrice. The cells were then incubated with anti-rabbit Alexa fluor 555 secondary antibody at a dilution of 1:1000 for 1 h at room temperature. The secondary antibody was then removed and the cells were washed with 0.1% PBST thrice. Further, the cells were stained with DAPI and then the coverslips were mounted on the slides.

### sgRNA designing and cloning in dCas9-DNMT3A expression vector and cell transfection

sgRNAs were designed using CRISPick (https://portals.broadinstitute.org/gppx/crispick/public) and CHOPCHOP (https://chopchop.cbu.uib.no). It was made sure that the sgRNA spanned around the binding sites of transcription factors of interest. All the sgRNAs were annealed and cloned into the *BbsI* site of pdCas9-DNMT3A-PuroR_v2 (Addgene #74407). Before using these cloned sgRNAs for experiments the plasmids were sequence verified. Details for all sgRNA used in this study are provided in Supplementary Table S1. MCF7 cells seeded at 80% confluency were transfected with sgRNAs cloned in pdCas9-DNMT3A-PuroR_v2 targeting the region of interest using 1:3 ratio of DNA:TurboFect (Thermo Scientific, Cat No. R0531) according to the manufacturer’s instructions. Twelve hours post transfection the cells were supplemented with fresh media and treated under hypoxic conditions. These cells were subsequently used for harvesting protein, MeDIP and ChIP.

### RNA-seq data analysis

Total RNA was extracted from control and *ATF3* knockdown, after 24 h of hypoxic treatment using TRIzol (*n* = 1) (Invitrogen, 15596026). rDNase (Macherey-Nagel, 740963) treatment was given for 10 min at 37 °C. 3 μg of total RNA was used for library preparation. Before library preparation mRNA enrichment was done using NEBNext^®^ Poly(A) mRNA Magnetic Isolation Module (E7490S). The libraries were constructed using NEBNext^®^ Ultra^™^ II RNA Library Prep Kit for Illumina^®^ (E7770S) and sequenced on Illumina NovaSeq sequencing system (Neuberg Center for Genomic Medicine, Ahmedabad, India). Raw sequencing reads were aligned to the human reference genome (hg38 assembly) with STAR Aligner (version 2.7.1a). Quantification of gene expression was performed using featureCounts from Subread (version 2.0.0). Differentially expressed genes (DEGs) in control vs *ATF3* knockdown cells were identified using NOIseq (version 2.44.0) analysis [30]. Genes with probability of difference of 0.6 or more were called as DEGs. The RNA-seq data generated is deposited under the GEO accession number: GSE274936. List of genes downregulated in *ATF3* knockdown under hypoxia are mentioned in Supplementary Table S4.

### Retrieval of publicly available datasets and their analyses

Raw reads files of RNA-seq data for GSE166203 and GSE71401 datasets was retrieved from GEO. The raw sequencing reads were aligned to the human reference genome (hg38 assembly) with STAR Aligner (version 2.7.1a). Quantification of gene expression was performed using featureCounts from Subread (version 2.0.0). For GSE166203 Differentially expressed genes (DEGs) in normoxia vs 5 h, 10 h, and 16 h of hypoxia were identified using NOIseq (version 2.44.0) analysis. Log2(ratio) values of the normoxic and hypoxic means for genes belonging to the ATF/CREB family were used to generate a heatmap using MORPHEUS (https://software.broadinstitute.org/morpheus/). For GSE71401 DEGs in normoxia vs hypoxia were identified using DESeq2 (version 1.40.2) analysis. MaGIC Volcano Plot Tool (https://volcano.bioinformagic.tools) was used to plot the Log2(ratio) values. List of genes along with their Log2(ratio) values are provided in Supplementary Table 5. For HTA 2.0 microarray dataset GSE190401, the CEL files were analyzed using Transcriptome Array Console 4.0 (Invitrogen, version 4.0.2.15) using the gene + exon-SST-RMA method of summarization. Fold change >2, *P* < 0.05 and False Discovery Rates (FDRs) < 0.05 was set as the threshold to identify the DEGs.

### Statistical analysis

All experiments were performed at least thrice, unless otherwise specified. Statistical analyses for all data is presented as mean values ± SD (*n* = 3 biological replicates, unless otherwise specified) as calculated using either two-tailed Student’s t-test (for comparisons between two groups) or one-way ANOVA with Dunnett’s multiple comparisons test (for comparisons between more than two groups). The statistical tests used for each analysis are mentioned within the figure legends or respective materials and methods sections. *P*-value less than 0.05 was considered to be significant. *P ≤ 0.05, **P ≤ 0.01, ***P ≤ 0.001, ns = not significant. GraphPad Prism 9 was used for all analyses. RStudio (version 2024.04.2 + 764) was used for analysis of RNA-seq data.

## Results

### ATF3 is induced under hypoxic stress via HIF1ɑ in breast cancer cells

**Fig. 1:**
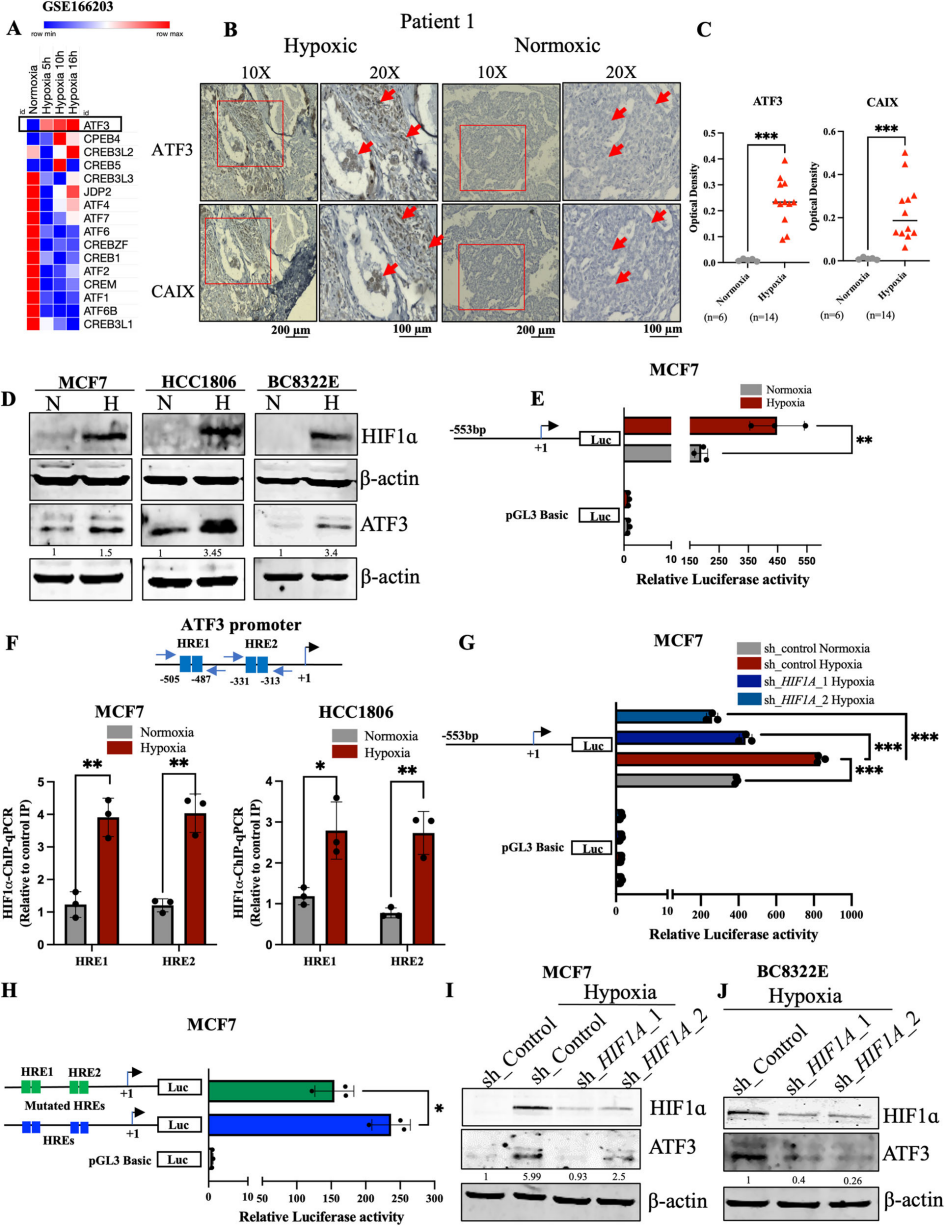
Regulation of ATF3 induction in hypoxic breast cancer cells via HIF1ɑ. **A** Heatmap representation of ATF/CREB family members from a Normoxia Vs Hypoxia RNA seq data analysis (GSE166203). **B** Immunohistochemistry for ATF3 and CAIX in breast cancer patient tissue sample and **C** quantification for the same, Mann-Whitney test was used for statistical comparison (scale bars, 10×, 200 µm; 20×, 100 µm). **D** Immunoblot for HIF1ɑ and ATF3 showing induction in hypoxia in MCF7, HCC1806 breast cancer cell lines and BC8322E patient derived cell line. **E** Luciferase promoter assay for *ATF3* promoter in MCF7 cells. **F** Schematic for position of HREs on the *ATF3* promoter and HIF1ɑ-ChIP-qPCR showing the increased occupancy of HIF1ɑ under hypoxia in MCF7 and HCC1806 cells. **G** Luciferase promoter assay for *ATF3* promoter showing decreased activity in *HIF1A* knockdown MCF7 cells (one-way ANOVA). **H** Luciferase promoter assay comparing wild type pGL3_*ATF3*pro to mutant pGL3_*ATF3*pro luciferase activity in MCF7 cells (one-way ANOVA). **I** Immunoblot depicting decreased expression of ATF3 in *HIF1A* knockdown MCF7 cells. **J** Immunoblot showing decreased expression of ATF3 in *HIF1A* knockdown BC8322E patient derived cell line. Error bars show mean values ± SD (*n* = 3 (biological replicates), unless otherwise specified) as calculated using two-tailed Student’s t-test, unless otherwise specified, *P ≤ 0.05, **P ≤ 0.01, ***P ≤ 0.001, ns, not significant.

Since our interest lies in understanding the adaptation of breast cancer to hypoxia, we started with identifying which members of the ATF/CREB family showed induced expression when subjected to reduced oxygen concentrations. Upon investigating a previously reported RNA-seq dataset performed in MCF7 cells (GSE166203) [31], we found that, *ATF3* showed significant increase in expression under hypoxic conditions as compared to normoxia (Fig. 1A). Another RNA-seq dataset performed in MCF7 (GSE71401) [32] and an HTA 2.0 analysis previously reported by our group (GSE190401) [33] also revealed that *ATF3* was induced under hypoxic conditions (Fig. S1A, B). We also confirmed the induction of ATF3 in immunohistology analysis of breast tissue sections obtained from breast cancer patients. The hypoxic niches generated within the breast tumors positively stained for ATF3 and coincided with the staining of a universal hypoxia marker CAIX, showing a positive correlation (Figs. 1B, C and S2). Since MCF7 and HCC1806 are the widely studied breast cancer cell lines, we have used them throughout this study to validate that our findings are not restricted to a particular breast cancer subtype. Similar to the RNA-seq findings, we also detected induction of ATF3 under 1% O_2_ from our immunoblot analysis in MCF7, HCC1806 as well as a patient derived cell line BC8322E, expression of HIF1ɑ confirms the hypoxic induction. (Fig. 1D).

As the induced expression was also reflected in the qPCR analysis performed to quantify the mRNA transcript levels of *ATF3* (Fig. S1C), we speculated a transcriptional upregulation of *ATF3* under hypoxic conditions. To validate the role of transcriptional regulation for induction of the *ATF3* gene, we generated delete construct of the *ATF3* promoter and cloned it into a luciferase reporter construct. The luciferase assay performed with pGL3_*ATF3*pro construct showed increased activity under hypoxia compared to normoxia control (Figs. 1E and S1E). Interestingly, we found HIF1ɑ binding sites known as hypoxia responsive elements (HREs) within the pGL3_*ATF3*pro construct region (Fig. 1F). To verify occupancy of HIF1ɑ on *ATF3* promoter at these HREs we performed HIF1ɑ-ChIP-qPCR analysis. As compared to normoxia, we found that HIF1ɑ binding increased at these HREs under hypoxic conditions, however, there was no significant increase in HIF1ɑ binding at 10 kb upstream of the *VEGFA* promoter, which has earlier been shown as a negative control for HIF1ɑ-ChIP-qPCR [34] (Figs. 1F and S1D). To further authenticate the role of HIF1ɑ as the transcriptional regulator of *ATF3* under hypoxia we performed a luciferase assay using pGL3_*ATF3*pro in control and *HIF1A* knockdown and knockout cells. Luciferase activity of pGL3_*ATF3*pro decreased in MCF7 *HIF1A* knockdown cells as well as HCC1806 *HIF1A* knock out cells (Figs. 1G and S1F). To ensure the significance of the HREs present within pGL3_*ATF3*pro and the necessity of binding of HIF1ɑ at the *ATF3* promoter, we selectively mutated both HREs using site-directed mutagenesis. When wild-type pGL3_*ATF3*pro was compared to mutant pGL3_*ATF3*pro under hypoxic condition we found that luciferase activity of the mutant pGL3_*ATF3*pro was relatively reduced (Figs. 1H and S1G). We also validated the role of HIF1ɑ in ATF3 induction at protein level and observed that ATF3 expression was reduced in *HIF1A* knockdown and knockout cells as compared to control MCF7 and BC8322E cells (Fig. 1I, J) as well as wild-type HCC1806 cells (Fig. S1H), respectively. Thus, these results prove HIF1ɑ to be the transcriptional regulator of ATF3 under hypoxic condition.

### *ATF3* promoter undergoes epigenetic alterations under hypoxia

**Fig. 2:**
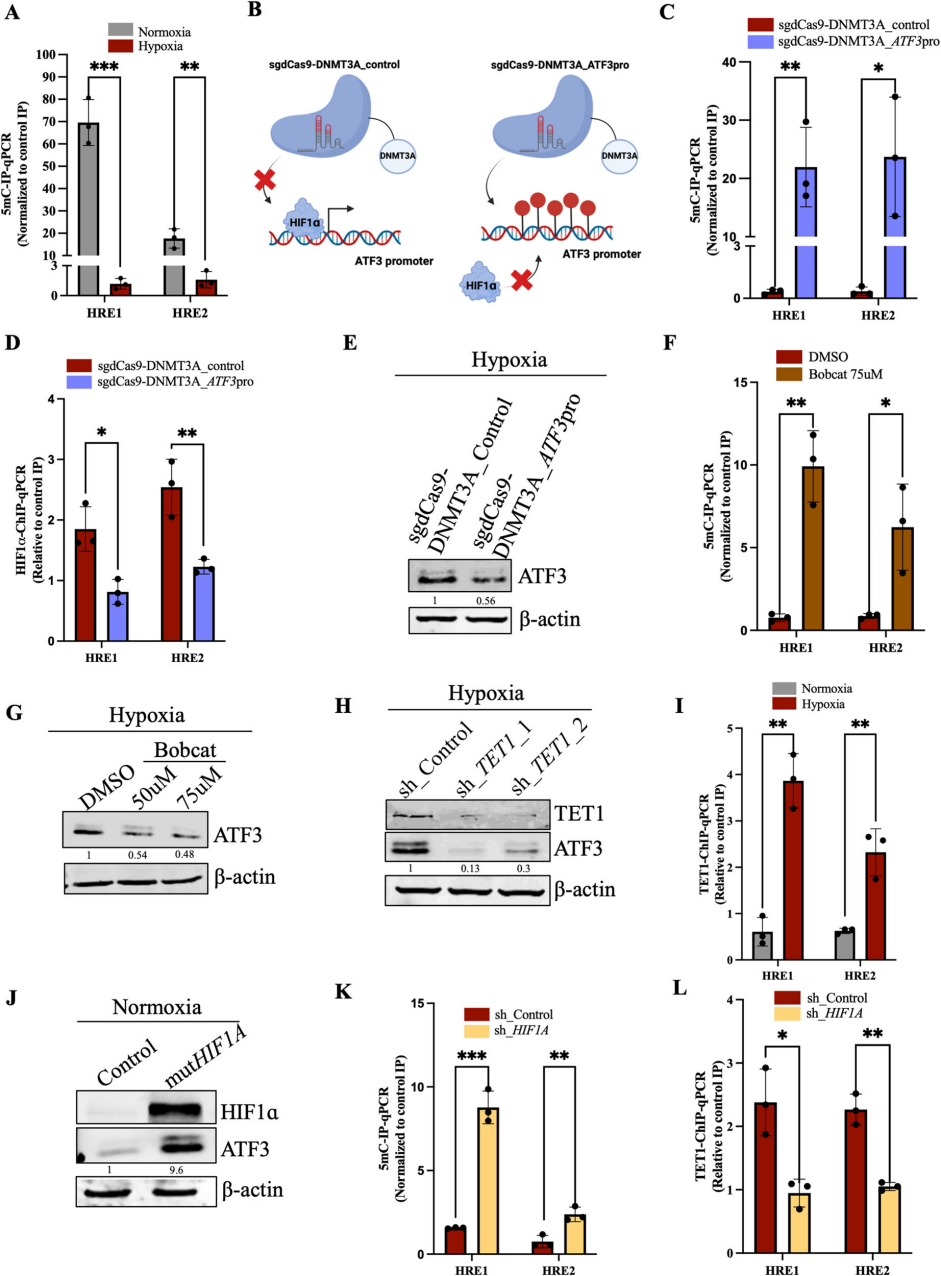
Epigenetic basis of regulation of *ATF3* transcription under hypoxia. **A** MeDIP-qPCR showing decreased methylation at the *ATF3* promoter under hypoxia in MCF7 cells. **B** Schematic representation of mode of function of sgdCas9-DNMT3A_ATFpro targeting the *ATF3* promoter and maintaining methylation to inhibit HIF1ɑ binding. **C** MeDIP-qPCR showing increased methylation at *ATF3* promoter in sgdCas9-DNMT3A_ATF3pro transfected MCF7 cells under hypoxia. **D** HIF1ɑ-ChIP-qPCR depicting decreased occupancy of HIF1ɑ at the *ATF3* promoter in sgdCas9-DNMT3A_ATF3pro transfected MCF7 cells compared to the sgdCas9-DNMT3A_control transfected control MCF7 cells under hypoxia. **E** Immunoblot comparing ATF3 expression in sgdCas9-DNMT3A_ATF3pro transfected MCF7 cells and sgdCas9-DNMT3A_control transfected MCF7 cells under hypoxia. **F** MeDIP-qPCR showing increased methylation at *ATF3* promoter after Bobcat treatment in MCF7 cells under hypoxic condition. **G** Immunoblot for ATF3 expression after Bobcat treatment revealing decreased expression of ATF3 after treatment in MCF7 cells under hypoxic condition. **H** Immunoblot showing decreased expression of ATF3 in *TET1* knockdown MCF7 cells under hypoxic condition. **I** TET1-ChIP-qPCR showing increased occupancy of TET1 at *ATF3* promoter in MCF7 cells under hypoxia. **J** Immunoblot for comparing ATF3 expression in control and mut *HIF1A* transfected MCF7 cells under normoxic condition. **K** MeDIP-qPCR showing increased methylation at *ATF3* promoter in *HIF1A* knockdown MCF7 cells under hypoxic condition. **L** TET1-ChIP-qPCR showing decreased occupancy of TET1 at *ATF3* promoter in *HIF1A* knockdown MCF7 cells under hypoxic condition. Error bars show mean values ± SD (*n* = 3 (biological replicates), unless otherwise specified) as calculated using two-tailed Student’s t-test, unless otherwise specified, *P ≤ 0.05, **P ≤ 0.01, ***P ≤ 0.001, ns, not significant.

The converse relation between HIF1ɑ binding and the methylation status of a particular DNA region is long known [35]. Notably, demethylation of promoter region of a gene is often indispensable for its transcription as it offers access to several factors. As we encountered a dense CpG island at the *ATF3* promoter region encompassing HREs (Fig. S3A), we further checked the role of demethylation as a salient prerequisite for HIF1ɑ binding and the resulting transcriptional upregulation of *ATF3*. Our MedIP-qPCR analysis showed that *ATF3* promoter was methylated at the HREs in normoxic conditions and underwent demethylation when under hypoxia (Figs. 2A and S3B). To authenticate the requirement of demethylation of *ATF3* promoter at the HREs for its induction under hypoxia, we employed the dCas9-DNMT3A, an epigenetic modulation system, to ectopically maintain methylation at the HREs guided by a sgRNA complimentary to the promoter region. dCas9-DNMT3A_ATF3pro maintained methylation at the 5mC residues present at the HREs under hypoxia as compared to the dCas9-DNMT3A_Control (Figs. 2C and S3C). As a result of the steric hinderance caused by increased methylation at the HREs, HIF1ɑ binding at *ATF3* promoter HREs reduced as observed in the HIF1ɑ-ChIP-qPCR analysis (Figs. 2D and S3D) and depicted in the schematic in Fig. 2B. Also, ATF3 expression was found to be decreased in the dCas9-DNMT3A_ATF3pro transfected cells (Figs. 2E and S3E). To understand the underlying mechanism by which demethylation occurs and if there was a role of the TET (Ten Eleven Translocases) enzymes, we used the Bobcat inhibitor. Firstly, we checked if Bobcat treatment could alter methylation at *ATF3* promoter HREs. By performing, MeDIP-qPCR we observed that even under hypoxic conditions Bobcat inhibited demethylation at *ATF3* promoter HREs (Figs. 2F and S3F). As speculated, ATF3 expression was also reduced upon Bobcat treatment under hypoxia (Figs. 2G and S3G). Further, to identify which TET was responsible for this action, we knocked down all three TET enzymes and checked for ATF3 expression, after treating them in hypoxic condition. Only the *TET1* knockdown cells showed reduced ATF3 expression under hypoxia as compared to control cells (Figs. 2H, S3H, I, J). TET1-ChIP-qPCR analysis also showed a significant increase in binding of TET1 at the *ATF3* promoter HREs under hypoxia (Figs. 2I and S3K). These results suggested that TET1 recruitment at *ATF3* promoter and resultant demethylation is necessary for HIF1ɑ binding and ATF3 induction under hypoxia.

Interestingly, we also found that overexpression of mut *HIF1A* (stable form of HIF1ɑ under normoxic conditions) could lead to induction of ATF3 even under normoxic condition (Figs. 2J and S3L). Furthermore, upon performing MeDIP-qPCR we found that *ATF3* promoter did not undergo demethylation in *HIF1A* knockdown and knockout cells under hypoxic conditions (Figs. 2K and S3M). It had been shown earlier that HIF1ɑ can physically bind to TET1 and conduct demethylation [36]. Thus, we investigated the possibility that TET1 occupancy at *ATF3* promoter was reliant on HIF1ɑ binding. The results of TET1-ChIP-qPCR indicated that HIF1ɑ is required for TET1 binding at the *ATF3* promoter because, in hypoxic conditions, TET1 occupancy at HREs was reduced in *HIF1A* knockdown and knockout cells (Figs. 2L and S3N). Similarly, *TET1* knockdown cells showed a decrease in HIF1ɑ occupancy at HREs, suggesting that TET1 is also required for HIF1ɑ binding (Fig. S3O, P). Consequently, HIF1ɑ and TET1 work together to modulate the *ATF3* promoter and carry out its transcription. In addition, we also observed ATF3 to be downregulated in a HTA 2.0 array dataset performed in *PKM2* knockout MCF7 cells under hypoxia (GSE190401) (Fig. S4A). Our group has earlier shown that PKM2 modulates the HIF1ɑ mediated transcription under hypoxia [33]. Therefore, we investigated if PKM2 has any role in the induction of ATF3 in hypoxic breast cancer cells. ATF3 protein levels were diminished in *PKM2* knockout cells under hypoxia as compared to wild type cells (Fig. S4B, C). Interestingly, we found PKM2 to bind *ATF3* promoter at the HREs from our PKM2-ChIP-qPCR analysis (Fig. S4D, E). PKM2 could affect the transcription of *ATF3* as the luciferase activity of pGL3_*ATF3*pro construct decreased in *PKM2* knockout cells (Fig. S4F). Loss of PKM2 did not show any significant increase in the HIF1ɑ occupancy at the *ATF3* promoter under hypoxia compared to normoxia as shown in *PKM2* knockout cells compared to wild-type cells. This resulted in decrease in the transcriptional activity of the *ATF3* promoter (Fig. S4G, H).

### ATF3 enhances invasive potential in breast cancer cells under hypoxic stress by inducing collagen organizing enzyme P4HA1

**Fig. 3:**
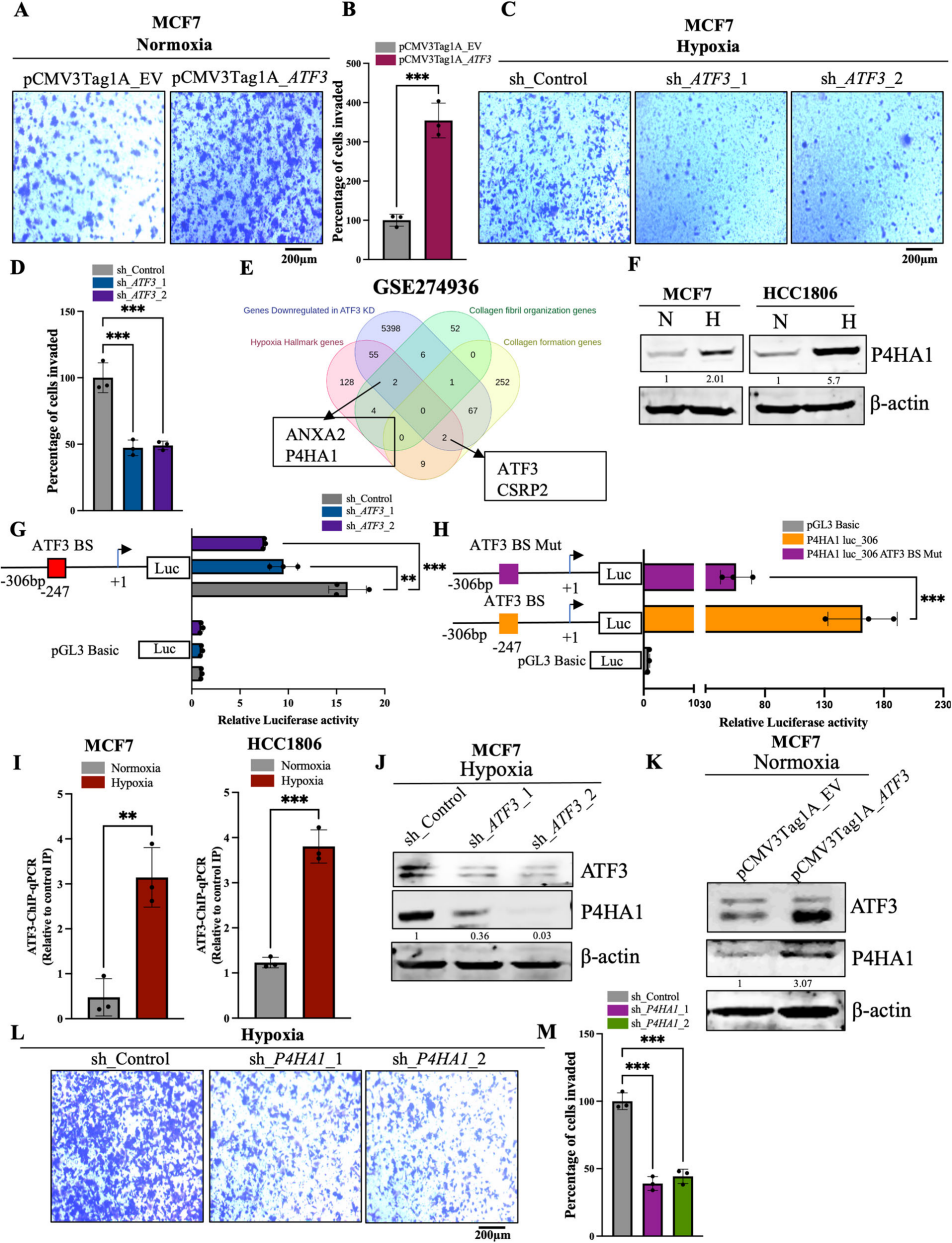
Enhancement of invasive potential of hypoxic breast cancer cells by ATF3 via induction of P4HA1. **A** Invasion assay and **B** its quantification in control and ATF3 overexpressing MCF7 cells. **C** Invasion assay and **D** its quantification in control and *ATF3* knockdown (two shRNA used) MCF7 cells (one-way ANOVA). **E** Venn diagram representing RNA seq analysis performed in *ATF3* knockdown cells and showing the common target genes. **F** Immunoblot for P4HA1 expression in normoxic and hypoxic MCF7 and HCC1806 cells. **G** Luciferase promoter assay for *P4HA1* promoter showing decreased activity in *ATF3* knockdown MCF7 cells (one-way ANOVA). **H** Luciferase promoter assay comparing wild type pGL3_*P4HA1*pro to mutant pGL3_*P4HA1*pro luciferase activity in MCF7 cells (one-way ANOVA). **I** ATF3-ChIP-qPCR showing increased occupancy of ATF3 at *P4HA1* promoter under hypoxia in MCF7 and HCC1806 cells. **J** Immunoblot showing decreased expression of P4HA1 in *ATF3* knockdown MCF7 cells under hypoxia. **K** Immunoblot showing increased expression of P4HA1 in *ATF3* overexpressing MCF7 cells under normoxia. **L** Invasion assay and **M** its quantification in control and *P4HA1* knockdown (two shRNA used) MCF7 cells (one-way ANOVA). Error bars show mean values ± SD (*n* = 3 (biological replicates), unless otherwise specified) as calculated using two-tailed Student’s t-test, unless otherwise specified, *P ≤ 0.05, **P ≤ 0.01, ***P ≤ 0.001, ns, not significant.

The induced expression of EMT-related factors, ECM enhancing factors, MMPs as well as factors involved in collagen synthesis, organization and deposition in the ECM contribute to the invasive nature of breast cancer cells under hypoxic conditions [7, 37–42]. ATF3 has also been known to enhance EMT and invasion in various cancer types by upregulating several EMT and cell motility-related genes [19–21]. As EMT and invasion are one of the aggressive phenotypes of breast cancer, we were interested to delineate the role of ATF3 in hypoxia induced invasion of breast cancer cells. To ensure ATF3’s function in the increased invasive potential of breast cancer, we performed overexpression of ATF3 using the *ATF3* cloned pCMV3Tag1a vector. Under normoxic conditions, overexpression of ATF3 led to increased invasion in both MCF7 and HCC1806 cell lines as compared to the empty vector control (Figs. 3A, B and S4I, J). In addition, when *ATF3* was knocked down the invasive potential of these cells was diminished (Figs. 3C, D and S4L, M). Since SNAIL is known to be an ATF3 target [19], we checked for SNAIL expression in ATF3 overexpressing as well as *ATF3* knockdown cells, which showed a positive correlation of SNAIL with ATF3 (Fig. S4K, N). Similarly, expression for SLUG and Vimentin also decreased in the *ATF3* knockdown cells while expression of the epithelial marker EpCAM was increased in the *ATF3* knockdown cells under hypoxic condition (Fig. S4O). These observations validated the role of ATF3 in invasiveness of hypoxic breast cancer cells.

Collagen induction and deposition in the ECM increases when cells experience hypoxia, this aids in the invasiveness of the cells as the collagen acts as highways for the invading cells. HIF1ɑ is also known to modify the collagen network under hypoxia [43, 44]. However, a major step in collagen deposition is the organization of collagen fibrils into a triple helical procollagen molecule. We performed RNA-seq analysis in *ATF3* knockdown cells to identify the ATF3 target genes which helps in hypoxia-mediated invasion via collagen modification (GSE274936). When we compared the list of genes which were downregulated in the *ATF3* knockdown cells under hypoxia with the list of genes involved in collagen formation and organization we found *P4HA1, ANXA2, CSRP2*, and *ATF3* to be the common target genes (Fig. 3E). P4HA1 is a collagen prolyl hydroxylase enzyme, which is found in the endoplasmic reticulum. Hydroxylation of the proline residues of the collagen polypeptides by P4HA1 is essential for formation of the triple helical collagen fibrils and is a prerequisite for collagen deposition in the ECM [45–48]. Interestingly, *P4HA1* was found to be the most upregulated gene under hypoxia in our HTA 2.0 analysis from GSE190401 (Fig. S4P). Considering the above verities, we chose P4HA1 for further studies to delineate the ATF3-dependent invasion of breast cancer cells in hypoxia.

Similar to HTA 2.0 data, *P4HA1* mRNA and protein expression levels were increased under hypoxia in both MCF7 and HCC1806 cells (Figs. 3F and S4Q). When we checked the *P4HA1* promoter using EPD tool and JASPAR we found a ATF3 binding site at −247 position upstream of the TSS (Fig. S4R). To validate the role of ATF3 in *P4HA1* transcriptional upregulation, we cloned the part of *P4HA1* promoter containing the ATF3 binding site in a luciferase reporter construct. Promoter luciferase activity of pGL3_*P4HA1*pro was reduced in *ATF3* knockdown cells as compared to control cells under hypoxia (Figs. 3G and S5A). Further, we also mutated the ATF3 binding site in pGL3_*P4HA1*pro construct using site-directed mutagenesis. The mutated pGL3_*P4HA1*pro showed a reduced luciferase promoter activity as compared to wild-type pGL3_*P4HA1*pro (Figs. 3H and S5B). We also checked for ATF3 binding at the *P4HA1* promoter by performing ATF3-ChIP-qPCR. Binding of ATF3 at −247 position upstream of TSS at *P4HA1* promoter was increased in hypoxic conditions (Fig. 3I). In addition, immunoblot analysis showed that the expression of P4HA1 was reduced in *ATF3* knockdown cells under hypoxia (Figs. 3J and S5C). Furthermore, when we overexpressed pCMV3Tag_*ATF3* in normoxic conditions it led to increased expression of P4HA1 (Figs. 3K and S5D). Like the *ATF3* knockdown cells, *P4HA1* knockdown cells also showed decreased invasive potential under hypoxia (Figs. 3L, M and S5E, F). Thus, these results suggest that ATF3 enhances invasiveness of the hypoxic breast cancer cells by upregulating the collagen organizing enzyme P4HA1, which is crucial for an invasion conducive ECM.

### P4HA1 undergoes alternative splicing under hypoxic conditions

**Fig. 4:**
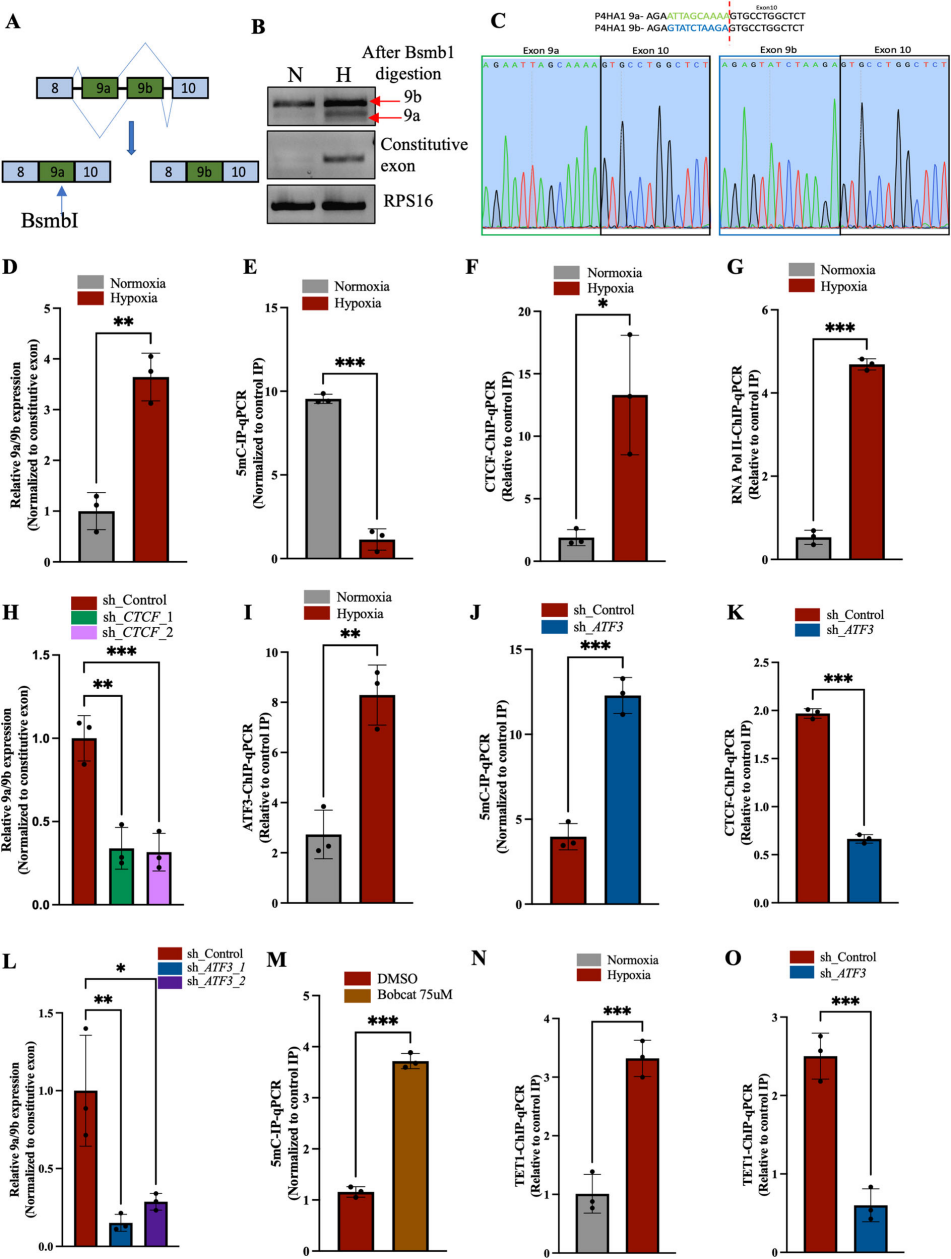
Mechanism of *P4HA1* alternative splicing under hypoxic conditions. **A** Schematic representation of *P4HA1* splicing, where either of the mutually exclusive exons (exon 9a (contains a BsmbI site) or exon 9b) are included. **B** Semi-quantitative PCR showing increased inclusion of exon 9a under hypoxia. **C** Chromatogram for Sanger sequencing of qPCR products obtained from primers which specifically amplify exon 9a and exon 9b. **D** qPCR analysis showing increased 9a/9b ratio in hypoxic conditions in MCF7 cells. **E** MeDIP-qPCR depicting decreased methylation at the intron 9a under hypoxia in MCF7 cells. **F** CTCF-ChIP-qPCR showing increased occupancy of CTCF at intron 9a in hypoxic MCF7 cells. **G** RNA Pol II-ChIP-qPCR showing increased occupancy of RNA Pol II at intron 9a in hypoxic MCF7 cells. **H** qPCR analysis showing decreased 9a/9b ratio in hypoxic *CTCF* knockdown MCF7 cells (**I**) ATF3-ChIP-qPCR showing increased occupancy of ATF3 at intron 9a in hypoxic MCF7 cells. **J** MeDIP-qPCR depicting increased methylation at the intron 9a under hypoxia in *ATF3* knockdown MCF7 cells. **K** CTCF-ChIP-qPCR showing decreased occupancy of CTCF at intron 9a in hypoxic *ATF3* knockdown MCF7 cells. **L** qPCR analysis showing decreased 9a/9b ratio in hypoxic *ATF3* knockdown MCF7 cells. **M** MeDIP-qPCR showing increased methylation at the intron 9a upon Bobcat treatment in hypoxic MCF7 cells. **N** TET1-ChIP-qPCR showing increased occupancy of TET1 at intron 9a in hypoxic MCF7 cells. **O** TET1-ChIP-qPCR showing decreased occupancy of TET1 at intron 9a in hypoxic *ATF3* knockdown MCF7 cells. Error bars show mean values ± SD (*n* = 3 (biological replicates), unless otherwise specified) as calculated using two-tailed Student’s t-test, unless otherwise specified, *P ≤ 0.05, **P ≤ 0.01, ***P ≤ 0.001, ns, not significant.

*P4HA1* pre-mRNA generates two isoforms in which either of the two mutually exclusive exons 9a or 9b are included in the mRNA transcript [23, 24]. However, the mechanism of *P4HA1* splicing and functional relevance of the two isoforms have not yet been elucidated. First, we checked which isoform was predominant under hypoxia. We could see in our semi-quantitative PCR analysis, where the product from *P4HA1* exon 8-exon 10 PCR was subjected to *BsmbI* digestion (which could only digest exon 9a (Fig. 4A)), that inclusion of exon 9a was increased however mRNA transcripts containing exon 9b were also present, as *P4HA1* itself upregulates under hypoxia (Fig. 4B). Similarly, qPCR analysis performed using junction primers that could specifically amplify the two separate isoforms (products were confirmed by sequencing (Fig. 4C)) showed that the ratio of 9a/9b was increased from normoxia to hypoxia, which suggested that inclusion of exon 9a increased under hypoxia (Figs. 4D and S5G).

When we scanned the region from exon 9a to exon 9b in JASPAR, we found a CTCF binding site at intron 9a (Fig. S5H). Since there are several evidences of differential CTCF binding affecting alternative splicing [25–27], we checked the possible involvement of CTCF in *P4HA1* splicing. Our MeDIP-qPCR analysis showed methylation at the CTCF binding site at intron 9a under normoxic conditions, which when exposed to hypoxia underwent demethylation (Figs. 4E and S5I). This corroborates with the previous findings which suggest CTCF binds specifically to unmethylated DNA regions [49–51]. Correspondingly, CTCF-ChIP-qPCR analysis ensured increased CTCF binding at intron 9a under hypoxic conditions (Figs. 4F and S5J). Further, to check if *P4HA1* splicing follows the CTCF-mediated RNA Pol II pause-dependent roadblock model, we also checked for occupancy of RNA Pol II along with CTCF at the intron 9a. As seen in RNA Pol II-ChIP-qPCR analysis, RNA Pol II binding also increased under hypoxic condition and decreased in *CTCF* knockdown cells (Figs. 4G and S5K, L, M, O, P). In addition, we also found that the ratio of 9a/9b decreased in *CTCF* knockdown cells as compared to the control cells under hypoxia (Figs. 4H and S5N). This confirmed that the step wise demethylation of CTCF binding site at intron 9a, CTCF binding, and RNA PolII pause are essential for inclusion of exon 9a.

Interestingly, along with CTCF we also found binding site of ATF3 just 380 bp downstream to the CTCF binding site (Fig. S5H). ATF3-ChIP-qPCR analysis showed that ATF3 binding at this site increased under hypoxic condition (Figs. 4I and S5Q). Even though DNA demethylation or influencing mRNA splicing are not canonical functions of ATF3, it was compelling to investigate if ATF3 played any role in *P4HA1* splicing. To our excitement *ATF3* knockdown under hypoxia resulted in increased methylation at the CTCF binding site (Figs. 4J and S5R). The increased methylation certainly hindered CTCF binding and reduced CTCF occupancy at intron 9a (Figs. 4K and S6A) leading to decreased inclusion of exon 9a, as showed in the qPCR analysis (Figs. 4L and S6B). The next step to unveil was the mechanism by which ATF3 attained demethylation at intron 9a. For this we first inhibited demethylation by using Bobcat to check if the reduced methylation at intron 9a was a result of active demethylation by the TET enzymes. Upon Bobcat inhibition the methylation at intron 9a increased (Figs. 4M and S6C), which suggested the role of a TET enzyme and that ATF3 probably recruits the TET enzyme at intron 9a. It was very intriguing that our TET1-ChIP-qPCR analysis showed that TET1 occupancy was increased at the intron 9a under hypoxia (Figs. 4N and S6D). Furthermore, TET1 occupancy decreased in *ATF3* knockdown cells, suggesting the importance of ATF3 at intron 9a for TET1 recruitment (Figs. 4O and S6E). Overall, there is a series of events taking place at intron 9a, where consecutive binding of ATF3, demethylation by TET1, recruitment of CTCF and RNA Pol II pause causes the inclusion of exon 9a.

### P4HA1 9a isoform renders invasive potential to breast cancer cells under hypoxia

**Fig. 5:**
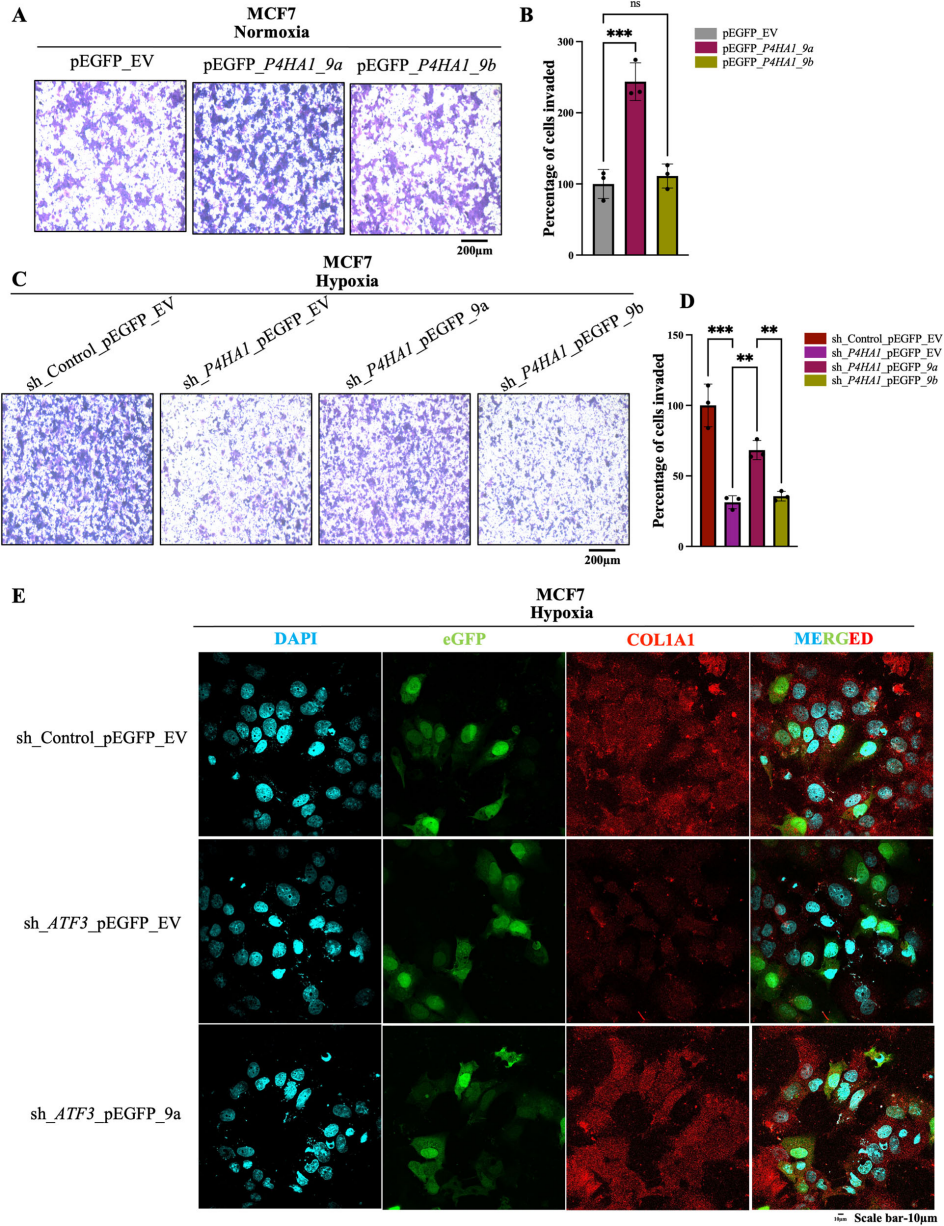
*P4HA1 9a* isoform increases the invasiveness of hypoxic breast cancer cells. **A** Invasion assay and **B** its quantification in control and *P4HA1 9a* or *9b* isoform overexpressing MCF7 cells under normoxic condition. **C** Invasion assay and **D** its quantification in *P4HA1* knockdown MCF7 cells rescued by overexpressing *P4HA1* 9a or 9b isoform as compared to the control *P4HA1* knockdown cells as well as the control cells transfected with a non-targeting shRNA in hypoxic conditions. **E** Immunostaining of COL1A1 in *ATF3* knockdown MCF7 cells which is then rescued by *P4HA1 9a* overexpression (DAPI staining is done to show the nucleus and overexpression of the *P4HA1* isoforms is confirmed by GFP, scale bars, 10 µm). Error bars show mean values ± SD (*n* = 3 (biological replicates), unless otherwise specified) as calculated using two-tailed Student’s t-test, unless otherwise specified, *P ≤ 0.05, **P ≤ 0.01, ***P ≤ 0.001, ns, not significant.

Finally, to assess the functional importance of the *P4HA1 9a* isoform and its role in hypoxia-mediated invasion of breast cancer cells, we overexpressed the two isoforms in wild type normoxic cells and *P4HA1* knockdown hypoxic cells. Under normoxic conditions, overexpression of *P4HA1 9a* led to increased invasion as compared to control cells as well as cells overexpressing *P4HA1 9b* isoform (Figs. 5A, B and S6J, K). Similarly, under hypoxic condition, the invasive potential had decreased in the *P4HA1* knockdown cells when compared to control cells, which could be rescued by the *P4HA1 9a* isoform but not by the *P4HA1 9b* isoform (Figs. 5C, D and S6L, M).

To assess if the enhanced invasion mediated by *P4HA1 9a* isoform was due to an increase in the deposition of collagen, we performed immunostaining assay for Collagen Type I alpha 1 chain (COL1A1). Collagen type I is the most abundant collagen in the breast tissue [52, 53]; therefore, it is an ideal choice to study the ECM composition in relation with elevated invasiveness. As *ATF3* was found to be one of the genes involved in collagen formation (Fig. 3E) and to check its direct effect on collagen deposition we checked for COL1A1 staining in *ATF3* knockdown cells. We observed that COL1A1 staining was decreased in *ATF3* knockdown cells under hypoxic conditions (Fig. S6O, P). However, there was no significant difference in the COL1A1 staining between control and *ATF3* knockdown cells in normoxic condition (Fig. S6O, P). Interestingly, the decreased COL1A1 deposition could be rescued by overexpression of *P4HA1 9a* isoform in the *ATF3* knockdown cells (Figs. 5E and S6N). Similarly, decreased COL1A1 deposition in *P4HA1* knockdown cells could be rescued with overexpression of *P4HA1*. However, overexpression of *P4HA1 9a* isoform led to more COL1A1 deposition as compared to *P4HA1 9b* isoform (Fig. S6Q). Moreover, we also checked for EMT-related genes SNAIL and Vimentin, these mesenchymal marker genes showed increased expression upon *P4HA1 9a* overexpression under normoxia (Fig. S6F, H). In addition, the decreased expression of SNAIL and Vimentin in *P4HA1* knockdown cells was restored upon *P4HA1 9a* overexpression as compared to *P4HA1 9b* overexpression under hypoxic condition (Fig. S6G, I). Thus, the *P4HA1 9a* isoform is increased under hypoxic conditions and renders the breast cancer cells more invasive by providing a more invasion conducive ECM.

## Discussion

Hypoxia is one of the major stressors within the tumor microenvironment of a solid tumor and influences an aggressive phenotype in the cancer cells. Under hypoxia, cancer cells experience a series of alterations in cellular processes, such as reduced proliferation, metabolic switch, induced autophagy, increased tolerance toward therapeutics and enhanced invasive potential. These changes are governed by the upregulation of a set of genes known as the hypoxia hallmark genes and are majorly regulated by HIF1ɑ, the master transcription factor of hypoxia. Apart from HIF1ɑ, other transcription factors can also be essential for the adaptation of cancer cells to hypoxia. One of such transcription factor is ATF3, an adaptive stress responsive gene belonging to the ATF/CREB family of transcription factors. Being localized on the chromosome 1q, the *ATF3* gene has a >2 copy number and is observed to show an elevated protein expression in breast cancer [19]. It has been observed to be involved in a positive feedback loop in which TGFβ regulates ATF3 expression, and the former then upregulates the latter [21]. ATF3 was discovered to be a dichotomous molecule that helps cancer cells evade apoptosis or cell cycle arrest by thwarting the harmful stress response. On the other hand, *ATF3* overexpression in untransformed cells increases the apoptotic pathway [19]. ATF3 also induces cell proliferation by activating CCND1 [54] and is a key regulator of paclitaxel-aggravated metastasis [55] and PI3K-AKT pathway mediated radio-resistance [56].

In this study, we identified *ATF3*, an adaptive response gene, to be induced in hypoxic breast cancer cells. Induction of ATF3 is a consequence of modulation of the epigenetic landscape of the *ATF3* promoter via synergistic efforts of HIF1ɑ and TET1, together with PKM2, where HIF1ɑ binding on the *ATF3* promoter is PKM2-dependent. The HIF1ɑ and TET1 duo decreases the methylation at *ATF3* promoter at the HREs and elevate HIF1ɑ binding to the unmethylated DNA leading to induction of ATF3 under hypoxia. This is an appealing aspect of this study as it provides insights into the less explored role of HIF1ɑ in regulation of promoter methylation. Inhibition of this epigenetic switch by either targeting TET1 or ectopically maintaining methylation at *ATF3* promoter leads to diminished induction of ATF3. This highlights the indispensable role of the epigenetic status of the *ATF3* promoter for its induction under hypoxia. ATF3 aids in adaptation to hypoxic conditions by increasing the invasive potential of the hypoxic cells. Role of ATF3 in enhancing EMT, cell motility and invasion has been studied previously, and in those reports the focus was majorly on the EMT and invasion-related genes. However, in this study, we have explored the regulation of a collagen organizing enzyme P4HA1 by ATF3. ATF3 actively binds to the *P4HA1* promoter and upregulates its transcription under hypoxia. P4HA1 is an endoplasmic reticulum residing collagen-prolyl hydroxylase, that performs a rate-limiting step in collagen organization and deposition in the ECM. P4HA1 has been individually studied to be essential for increasing collagen deposition and is associated with enhanced invasion phenotype.

Interestingly, we found that in addition to regulation of *P4HA1* transcription, ATF3 also plays a crucial role in the splicing of *P4HA1*. ATF3 binds at the intron 9a site leading to enhanced recruitment of TET1 at intron 9a. TET1 in turn demethylates this region making it accessible to CTCF. Owing to its specificity for unmethylated DNA, CTCF occupancy at intron 9a increases under hypoxic conditions as compared to normoxia. Following the roadblock model CTCF binding enhances the RNA Pol II pause downstream to exon 9a leading to increased inclusion of exon 9a under hypoxia. Furthermore, we found that the hypoxia-specific *P4HA1 9a* isoform could rescue the decreased collagen deposition in *ATF3* and *P4HA1* knockdown cells as well as the decreased invasion phenotype in *P4HA1* knockdown cells. The HIF1ɑ-ATF3-P4HA1 axis is therefore crucial for the invasion of hypoxic breast cancer cells. Thus, this study attempts to provide a comprehensive understanding of the emphasis of ATF3 in invasive hypoxic breast cancer cells. However, this might just be the beginning; there may be many unidentified routes through which ATF3 is implicated in adaptation of breast cancer cells to hypoxia. More research is needed to fully comprehend the role that ATF3 plays in alternative splicing of other genes and the adaptability of cancer cells to hypoxia.

## Supplementary information

Supplementary File 1

Supplementary File 2

Supplementary File 3

Supplementary Table 1

Supplementary Table 2

Supplementary Table 3

Supplementary Table 4

Supplementary Table 5

## Data Availability

The RNA-seq data generated in this study is deposited under the GEO accession number: GSE274936. All the other data supporting the findings in this study are included in the article and the supplementary information.
