# Supplementary material for: Hypoxia-induced ATF3 escalates breast cancer invasion by increasing collagen deposition via P4HA1
Source: Cell Death Dis. 2025 Feb 27;16(1):142. doi: 10.1038/s41419-025-07461-y (PMC11868403; doi:10.1038/s41419-025-07461-y)
Supplement: Supplementary file 1 [file 41419_2025_7461_MOESM1_ESM.pdf]

Supplementary Figures

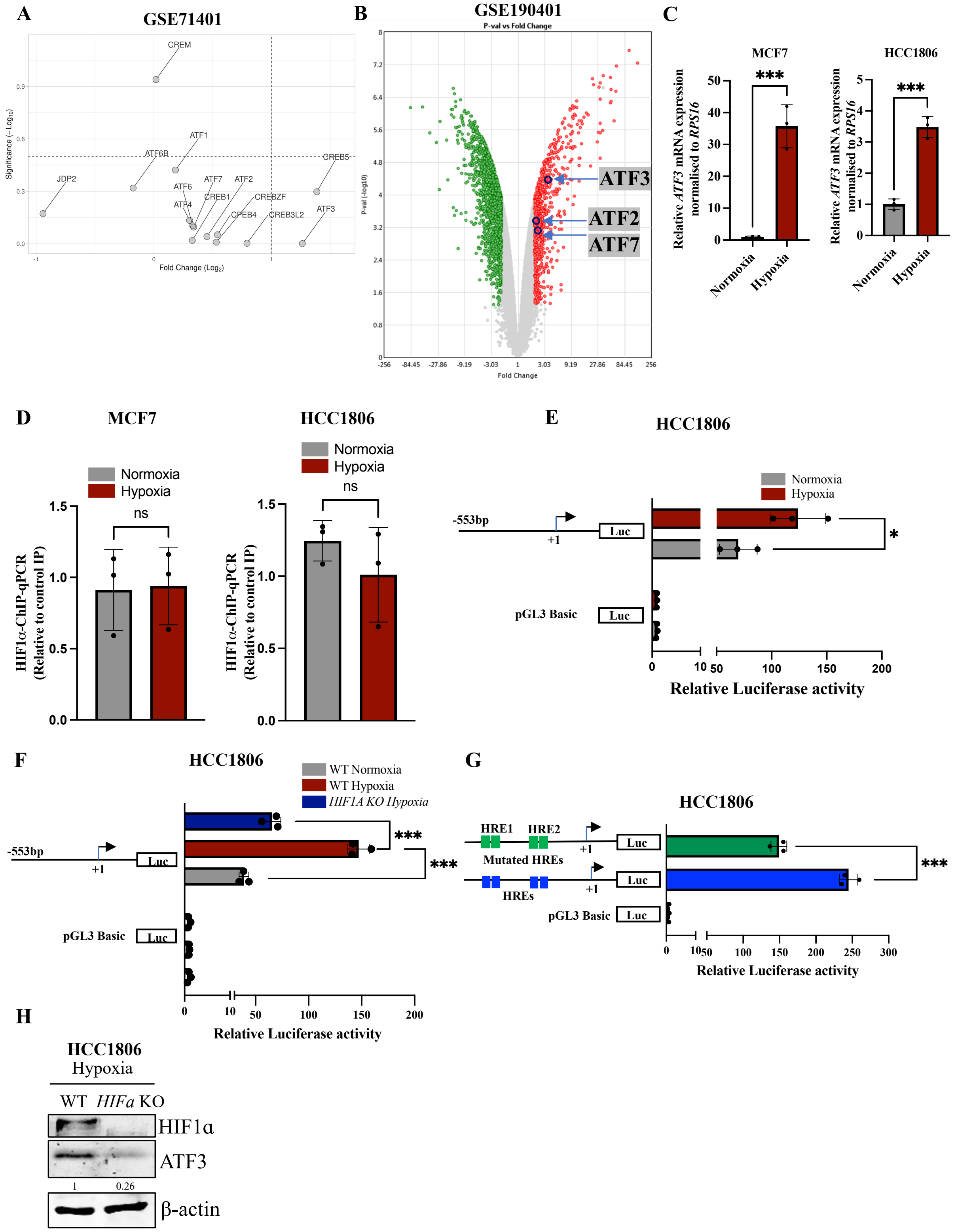

I

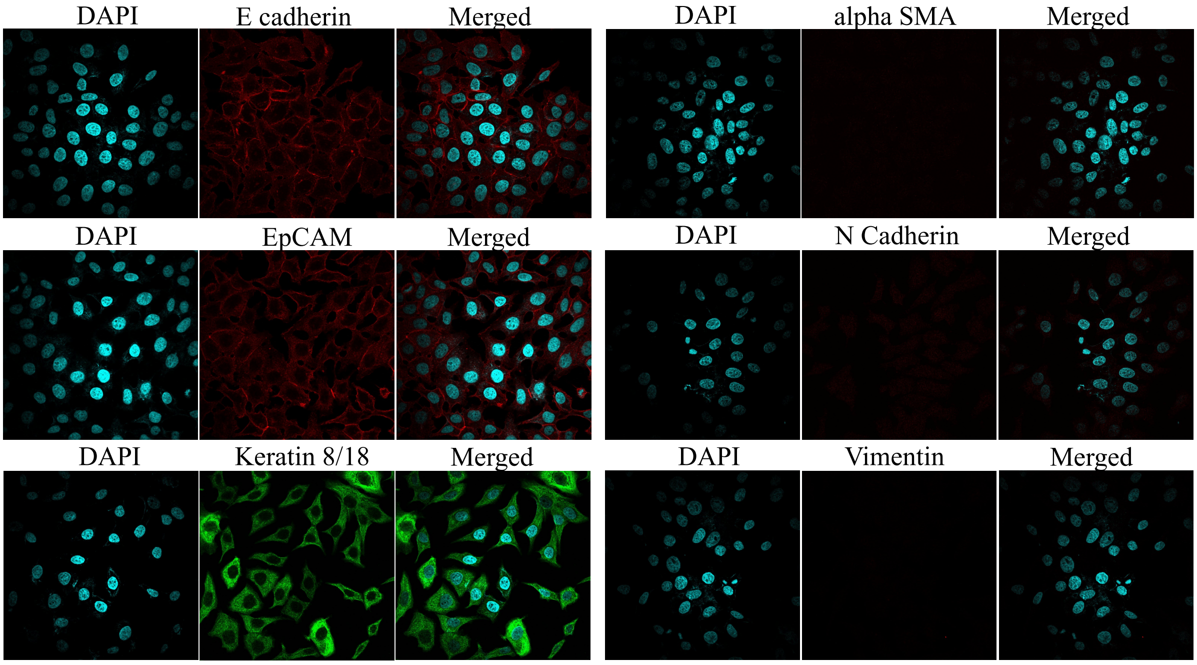

Figure S1. Regulation of ATF3 induction in hypoxic breast cancer cells via HIF1α. A) Heat map representation of ATF/CREB family members from a Normoxia Vs Hypoxia RNA seq data analysis (GSE71401) **B)** Volcano Plot from HTA 2.0 microarray data analysis (GSE190401) showing expression of ATFs, ATF3 is seen to be most significantly induced under hypoxia **C)** qPCR analysis for ATF3 showing induction in hypoxia in MCF7 and HCC1806 cells **D)** HIF1α-ChIP-qPCR showing no significant difference in the occupancy of HIF1α at a region 10kb upstream of TSS in VEGFA promoter in MCF7 and HCC1806 cells **E)** Luciferase promoter assay for *ATF3* promoter in HCC1806 cells **F)** Luciferase promoter assay for *ATF3* promoter showing decreased activity in *HIF1A* knockout HCC1806 cells (one-way ANOVA) **G)** Luciferase promoter assay comparing wild type pGL3_*ATF3*pro to mutant pGL3_*ATF3*pro luciferase activity in HCC1806 cells (one-way ANOVA) **H)** Immunoblot depicting decreased expression of ATF3 in *HIF1A* knockout HCC1806 cells **I)** Characterization of BC8322E patient derived cell line by immunostaining for E cadherin,

EpCAM, Keratin 8/18 (epithelial markers); N cadherin, Vimentin (mesenchymal markers) and alpha SMA (fibroblast marker). Error bars show mean values ± SD (n=3, unless otherwise specified) as calculated using two-tailed Student's t-test, unless otherwise specified, *$P \leq 0.05$, **$P \leq 0.01$, ***$P \leq 0.001$, ns = not significant.

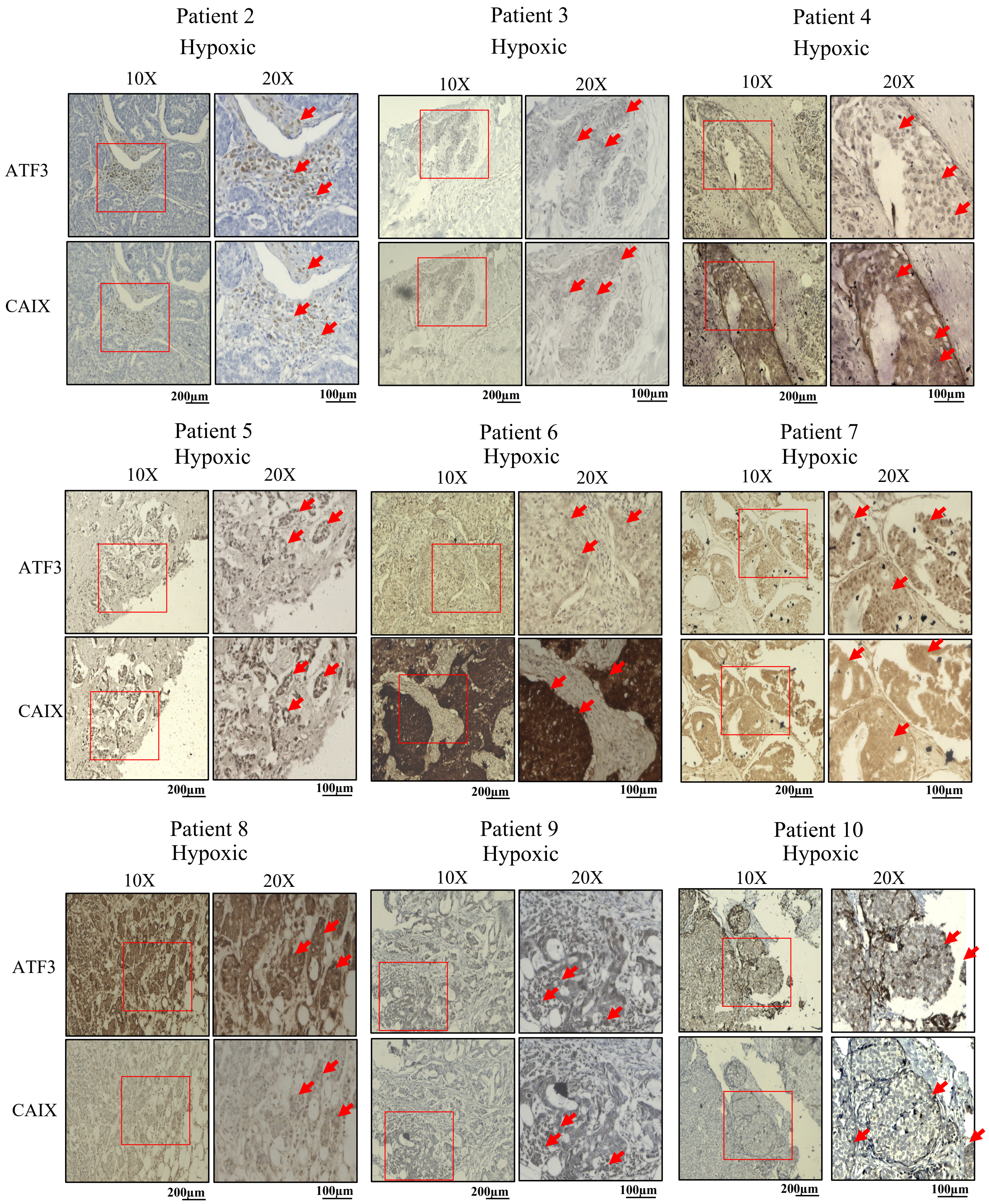

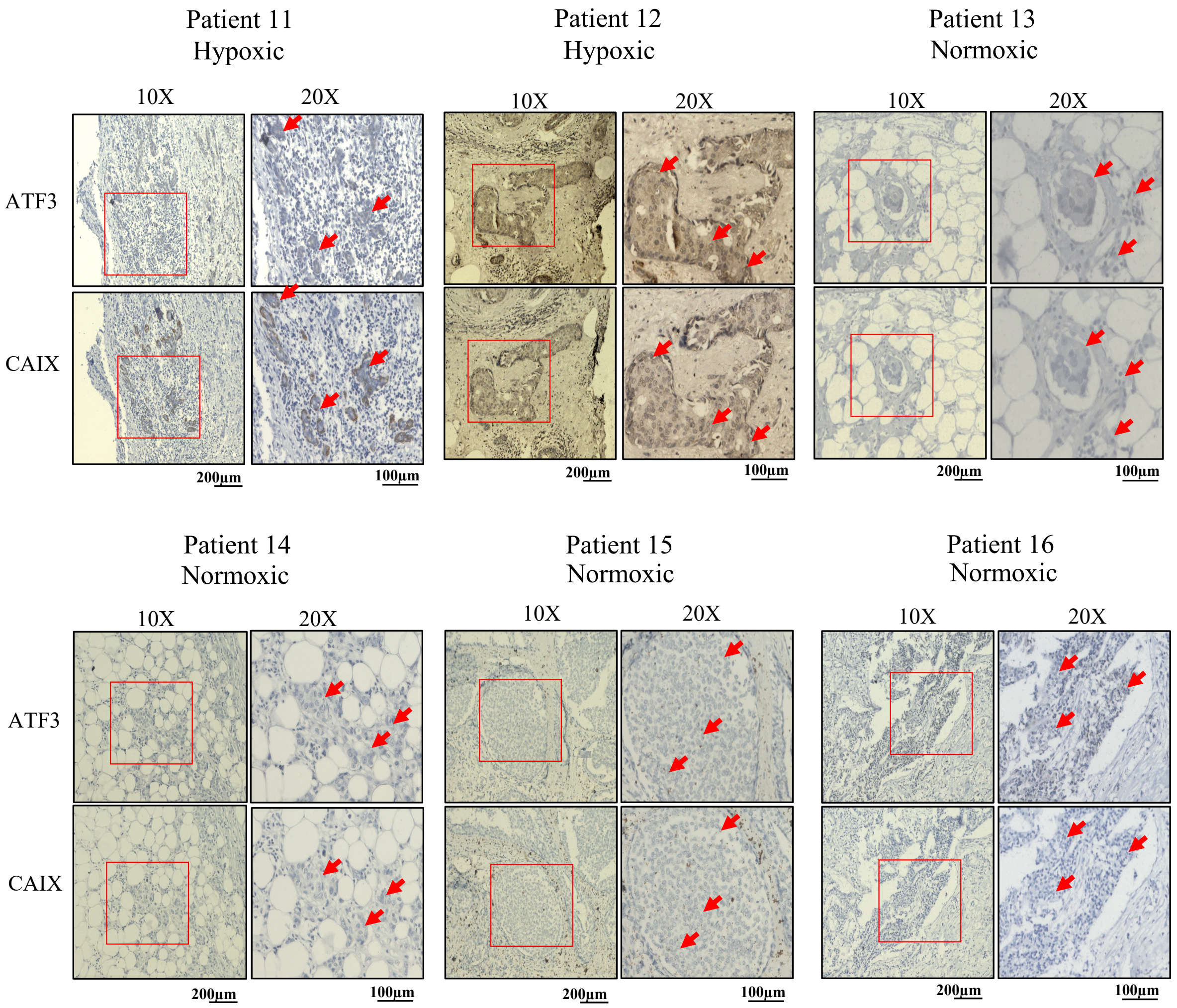

Figure S2. Immunohistochemistry for ATF3 and CAIX in breast cancer patient tissue samples (scale bars, 10×, 200 μm; 20×, 100 μm)

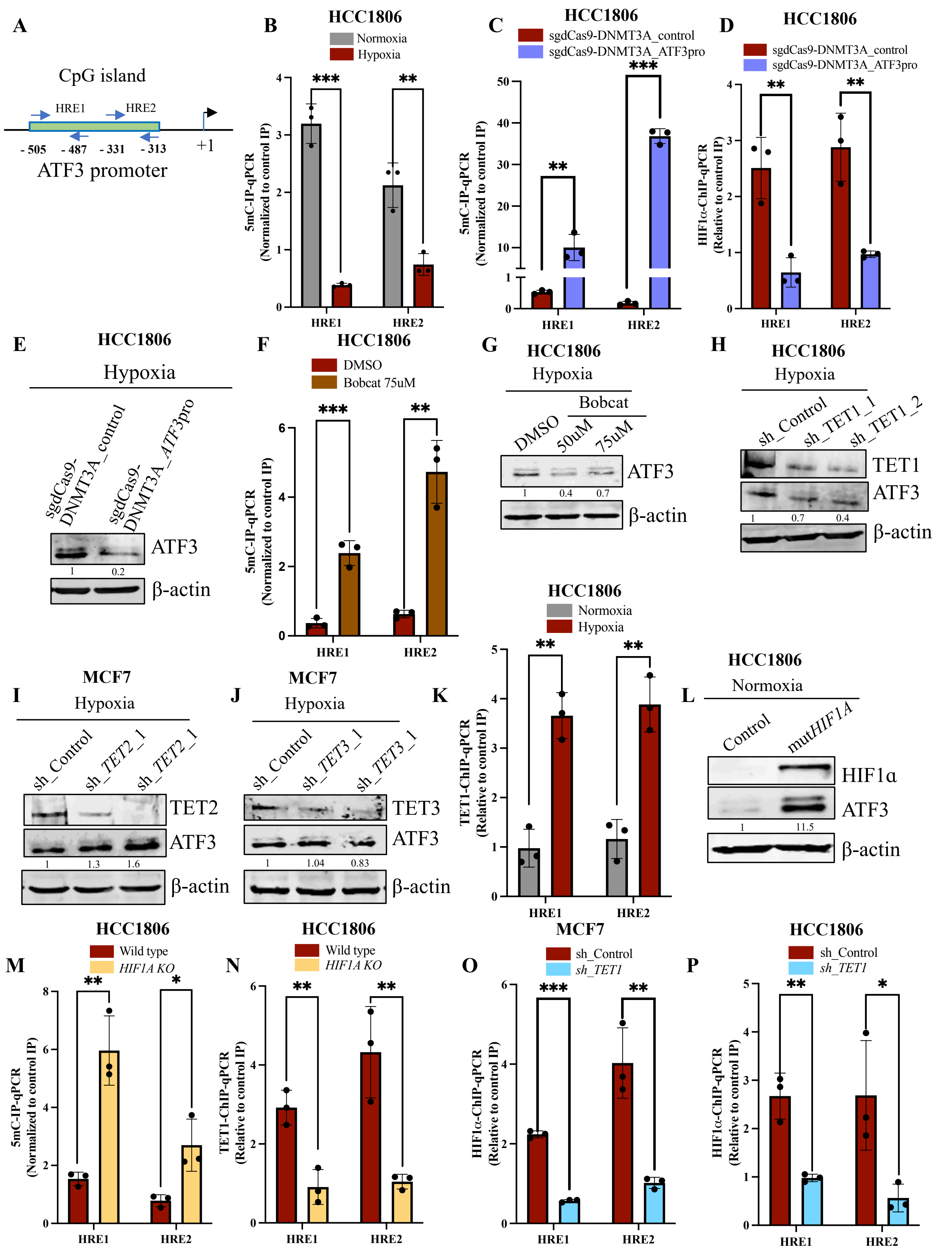

Figure S3. Epigenetic basis of regulation of *ATF3* transcription under hypoxia. A) Schematic representing the dense CpG island at the ATF3 promoter along with the positions of the primers used to study the two HREs **B)** MeDIP-qPCR showing decreased methylation

at the *ATF3* promoter under hypoxia in HCC1806 cells **C)** MeDIP-qPCR showing increased methylation at *ATF3* promoter in sgdCas9-DNMT3A_ATFpro transfected HCC1806 cells under hypoxia **D)** HIF1α-ChIP-qPCR depicting decreased occupancy of HIF1α at the *ATF3* promoter in sgdCas9-DNMT3A_ATFpro transfected HCC1806 cells compared to the sgdCas9-DNMT3A_control transfected control HCC1806 cells under hypoxia **E)** Immunoblot comparing ATF3 expression in sgdCas9-DNMT3A_ATFpro transfected HCC1806 cells and sgdCas9-DNMT3A_control transfected HCC1806 cells under hypoxia **F)** MeDIP-qPCR showing increased methylation at *ATF3* promoter after Bobcat treatment in HCC1806 cells under hypoxic condition **G)** Immunoblot for ATF3 expression after Bobcat treatment revealing decreased expression of ATF3 after treatment in HCC1806 cells **H)** Immunoblot showing decreased expression of ATF3 in *TET1* knockdown HCC1806 cells under hypoxic condition **I)** Immunoblot showing expression of ATF3 in TET2 knock down cells and **J)** TET3 knock down cells **K)** TET1-ChIP-qPCR showing increased occupancy of TET1 at *ATF3* promoter in HCC1806 cells under hypoxia **L)** Immunoblot for comparing ATF3 expression in control and mut *HIF1A* transfected HCC1806 cells under normoxic condition **M)** MeDIP-qPCR showing increased methylation at *ATF3* promoter in *HIF1A* knockout HCC1806 cells under hypoxic condition **N)** TET1-ChIP-qPCR showing decreased occupancy of TET1 at *ATF3* promoter in *HIF1A* knockout HCC1806 cells under hypoxic condition **O)** HIF1α-ChIP-qPCR showing decreased occupancy of HIF1α at *ATF3* promoter in *TET1* knockdown MCF7 cells and **P)** HCC1806 cells. Error bars show mean values ± SD (n=3, unless otherwise specified) as calculated using two-tailed Student's t-test, unless otherwise specified, *$P \leq 0.05$, **$P \leq 0.01$, ***$P \leq 0.001$, ns = not significant.

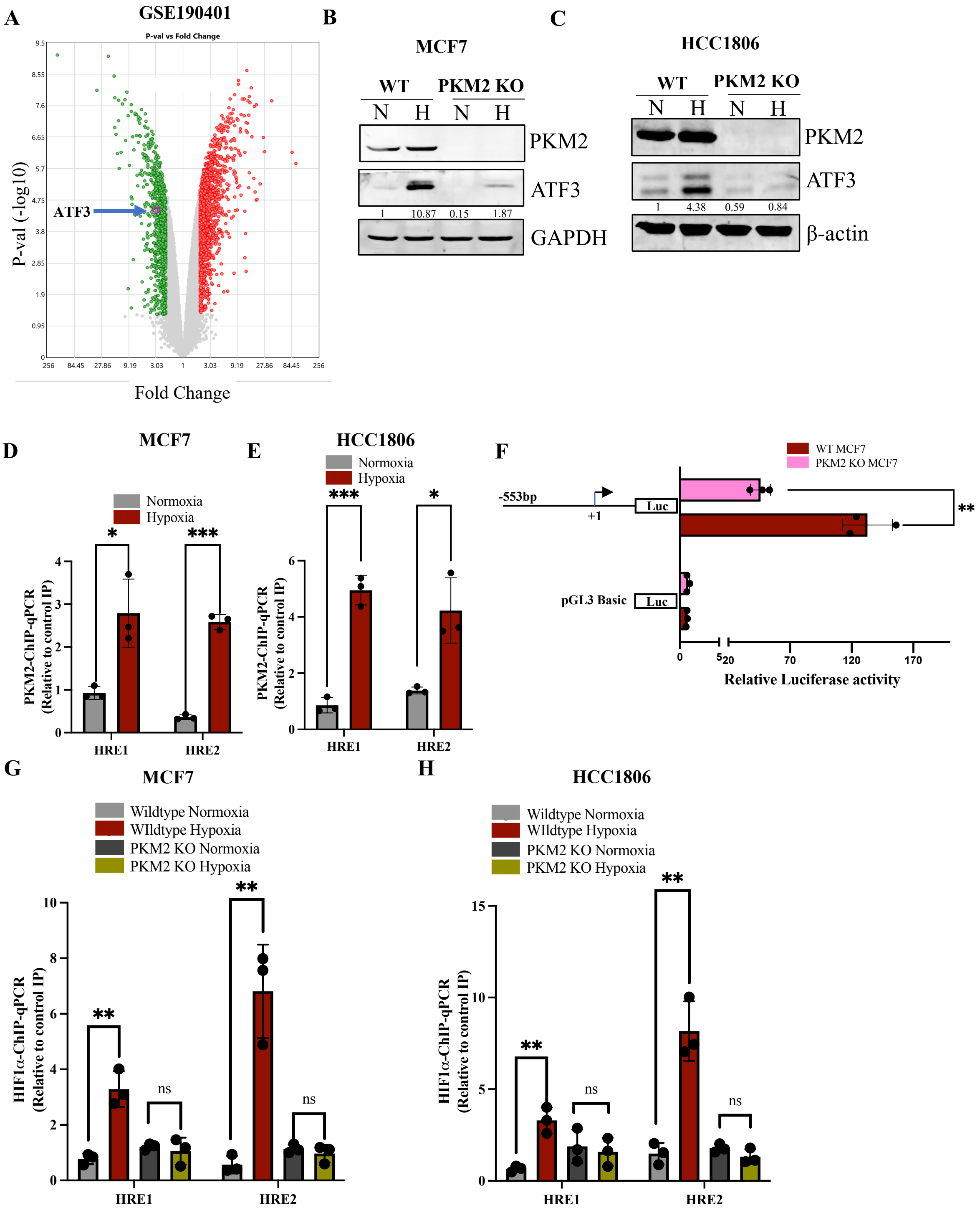

A
GSE190401
P-val vs Fold Change
P-val (-log10)
ATF3
Fold Change
B
MCF7
WT
PKM2 KO
N H N H
PKM2
ATF3
1 10.87 0.15 1.87
GAPDH
C
HCC1806
WT
PKM2 KO
N H N H
PKM2
ATF3
1 4.38 0.59 0.84
β-actin
D
MCF7
Normoxia
Hypoxia
PKM2-ChIP-qPCR (Relative to control IP)
HRE1
HRE2
E
HCC1806
Normoxia
Hypoxia
PKM2-ChIP-qPCR (Relative to control IP)
HRE1
HRE2
F
WT MCF7
PKM2 KO MCF7
-553bp
+1
Luc
pGL3 Basic
Luc
Relative Luciferase activity
G
MCF7
Wildtype Normoxia
WIldtype Hypoxia
PKM2 KO Normoxia
PKM2 KO Hypoxia
HIF1α-ChIP-qPCR (Relative to control IP)
HRE1
HRE2
H
HCC1806
Wildtype Normoxia
WIldtype Hypoxia
PKM2 KO Normoxia
PKM2 KO Hypoxia
HIF1α-ChIP-qPCR (Relative to control IP)
HRE1
HRE2

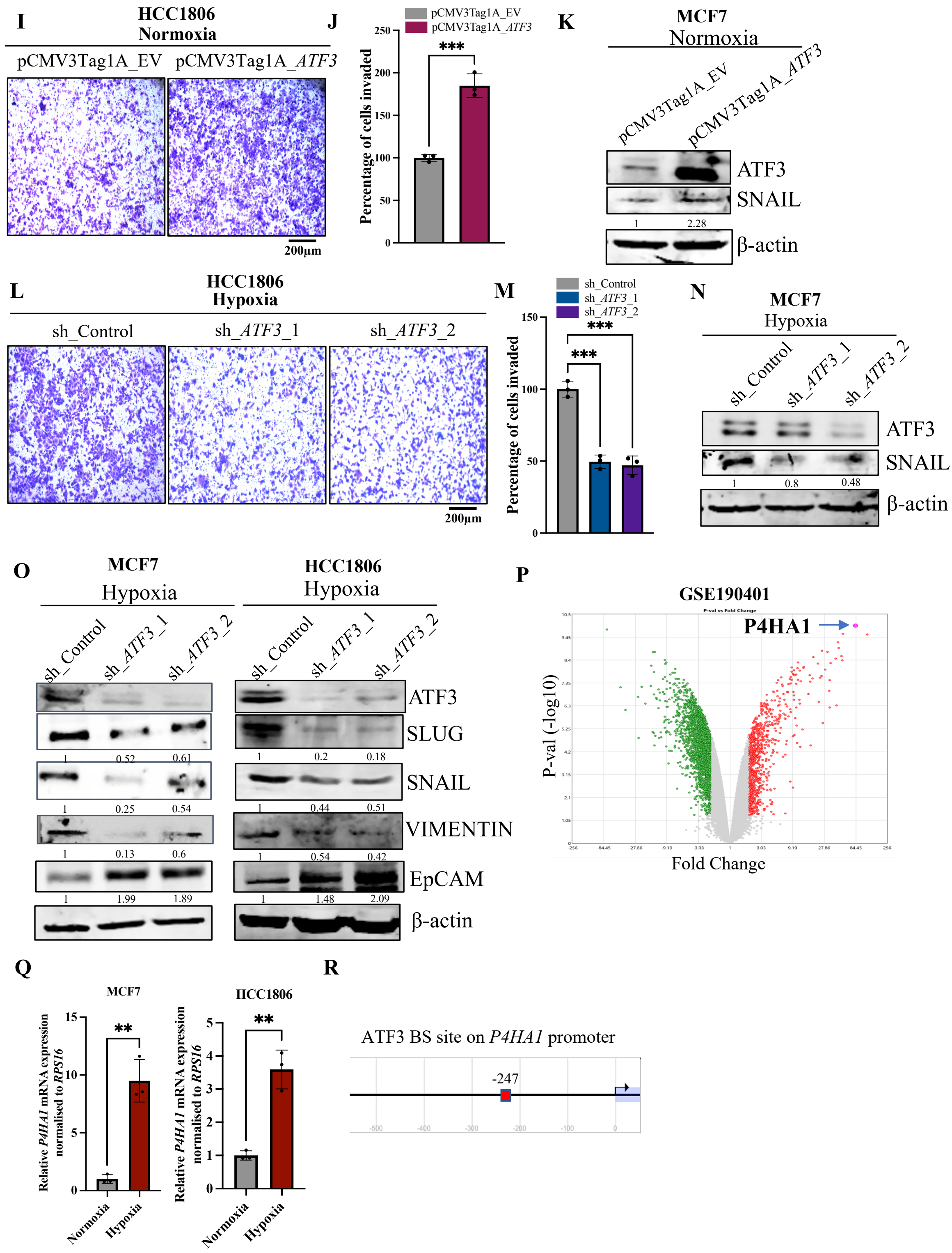

Figure S4. Role of PKM2 in regulation of *ATF3* transcription under hypoxia and enhancement of invasive potential of hypoxic breast cancer cells by ATF3 via induction of P4HA1. A) Volcano Plot from HTA 2.0 microarray data analysis (GSE190401) showing

decreased expression of ATF3 in *PKM2* knockout MCF7 cells under hypoxia **B)** Immunoblot showing expression of ATF3 in wild type and *PKM2* knockout MCF7 cells and **C)** HCC1806 cells **D)** PKM2-ChIP-qPCR showing increased occupancy of PKM2 at *ATF3* promoter in MCF7 cells and **E)** HCC1806 cells **F)** Luciferase promoter assay for *ATF3* promoter showing decreased activity in *PKM2* knockout MCF7 cells **G)** HIF1α-ChIP-qPCR showing increased occupancy of HIF1α at *ATF3* promoter in wildtype MCF7 cells and no significant difference in HIF1α occupancy in *PKM2* knockout MCF7 cells **H)** HIF1α-ChIP-qPCR showing increased occupancy of HIF1α at *ATF3* promoter in wildtype HCC1806 cells and no significant difference in HIF1α occupancy in *PKM2* knockout HCC1806 cells **I)** Invasion assay and **J)** its quantification in control and *ATF3* overexpressing HCC1806 cells **K)** Immunoblot showing increased expression of SNAIL in *ATF3* overexpressing MCF7 cells under normoxia **L)** Invasion assay and **M)** its quantification in control and *ATF3* knockdown (two hairpins used) HCC1806 cells (one-way ANOVA) **N)** Immunoblot showing decreased expression of SNAIL in *ATF3* knockdown MCF7 cells **O)** Immunoblot showing decreased expression of SLUG, SNAIL, Vimentin and increased expression EpCAM in *ATF3* knockdown MCF7 and HCC1806 cells **P)** Volcano Plot from HTA 2.0 microarray data analysis showing expression of P4HA1 to be most significantly induced under hypoxia (GSE190401) **Q)** qPCR analysis for P4HA1 expression in normoxic and hypoxic MCF7 and HCC1806 cells **R)** Schematic representation of ATF3 binding site on *P4HA1*promoter from Eukaryotic Promoter Database. Error bars show mean values $\pm$ SD (n=3, unless otherwise specified) as calculated using two-tailed Student's t-test, unless otherwise specified, *$P \leq 0.05$, **$P \leq 0.01$, ***$P \leq 0.001$, ns = not significant.

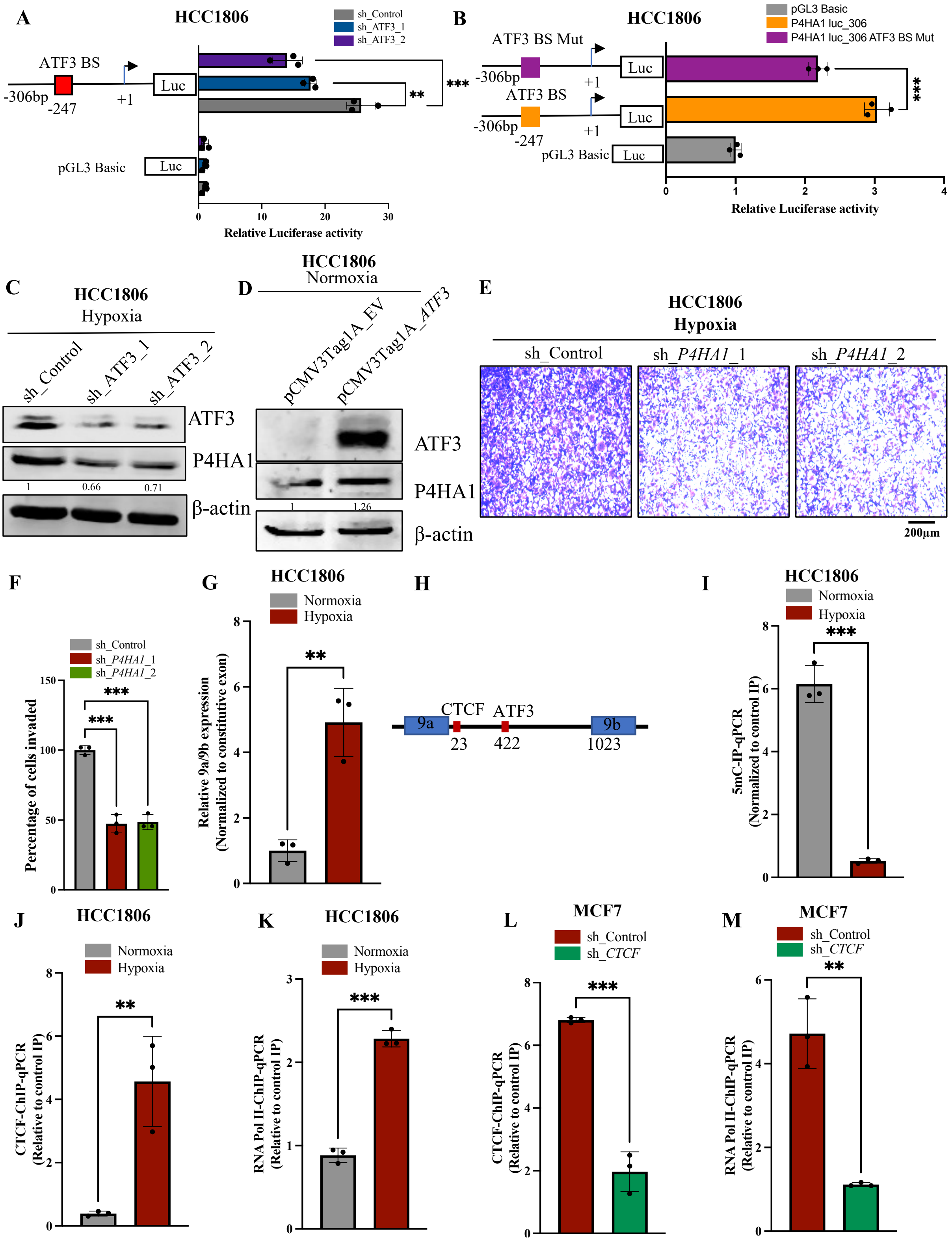
A
HCC1806
sh_Control
sh_ATF3_1
sh_ATF3_2
ATF3 BS
-306bp
-247
+1
Luc
pGL3 Basic
Luc
***
**
0
10
20
30
Relative Luciferase activity
B
HCC1806
pGL3 Basic
P4HA1 luc_306
P4HA1 luc_306 ATF3 BS Mut
ATF3 BS Mut
-306bp
+1
Luc
ATF3 BS
-306bp
-247
+1
Luc
pGL3 Basic
Luc
***
0
1
2
3
4
Relative Luciferase activity
C
HCC1806
Hypoxia
sh_Control
sh_ATF3_1
sh_ATF3_2
ATF3
P4HA1
1
0.66
0.71
β-actin
D
HCC1806
Normoxia
pCMV3Tag1A_EV
pCMV3Tag1A_ATF3
ATF3
P4HA1
1
1.26
β-actin
E
HCC1806
Hypoxia
sh_Control
sh_P4HA1_1
sh_P4HA1_2
200μm
F
sh_Control
sh_P4HA1_1
sh_P4HA1_2
***
***
Percentage of cells invaded
0
50
100
150
G
HCC1806
Normoxia
Hypoxia
**
Relative 9a/9b expression
(Normalized to constitutive exon)
0
2
4
6
8
H
CTCF
ATF3
9a
9b
23
422
1023
I
HCC1806
Normoxia
Hypoxia
***
5mC-IP-qPCR
(Normalized to control IP)
0
2
4
6
8
J
HCC1806
Normoxia
Hypoxia
**
CTCF-ChIP-qPCR
(Relative to control IP)
0
2
4
6
8
K
HCC1806
Normoxia
Hypoxia
***
RNA Pol II-ChIP-qPCR
(Relative to control IP)
0
1
2
3
L
MCF7
sh_Control
sh_CTCF
***
CTCF-ChIP-qPCR
(Relative to control IP)
0
2
4
6
8
M
MCF7
sh_Control
sh_CTCF
**
RNA Pol II-ChIP-qPCR
(Relative to control IP)
0
2
4
6

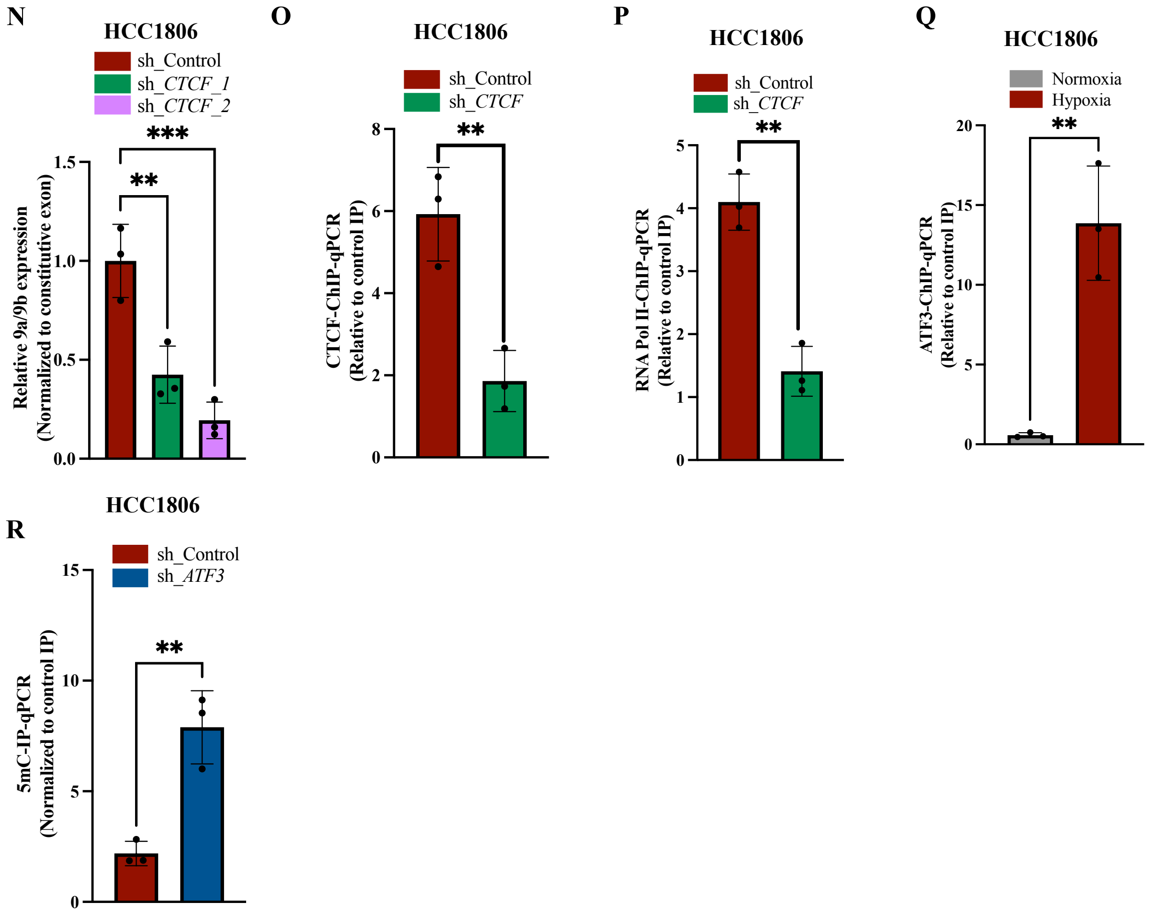

Figure S5. Role of ATF3 in P4HA1 induction under hypoxia and mechanism of *P4HA1* splicing under hypoxic condition. A) Luciferase promoter assay for *P4HA1* promoter showing decreased activity in *ATF3* knockdown HCC1806 cells (one-way ANOVA) **B)** Luciferase promoter assay comparing wild type pGL3_*P4HA1*pro to mutant pGL3_*P4HA1*pro luciferase activity in HCC1806 cells (one-way ANOVA) **C)** Immunoblot showing decreased expression of P4HA1 in *ATF3* knockdown HCC1806 cells **D)** Immunoblot showing increased expression of P4HA1 in *ATF3* overexpressing HCC1806 cells **E)** Invasion assay and **F)** its quantification in control and *P4HA1* knockdown (two hairpins used) HCC1806 cells (one-way ANOVA) **G)** qPCR analysis showing increased 9a/9b ratio in hypoxic conditions in HCC1806 cells **H)** Schematic showing positions of CTCF and ATF3 binding sites at intron9a as obtained from JASPAR **I)** MeDIP-qPCR depicting decreased methylation at the intron9a under hypoxia in HCC1806 cells **J)** CTCF-ChIP-qPCR showing increased occupancy of CTCF at intron9a in

hypoxic HCC1806 cells **K)** RNA PolII-ChIP-qPCR showing increased occupancy of RNA PolII at intron9a in hypoxic HCC1806 cells **L)** CTCF-ChIP-qPCR showing decreased occupancy of CTCF at intron9a in hypoxic *CTCF* knockdown MCF7 cells **M)** RNA PolII-ChIP-qPCR showing decreased occupancy of RNA PolII at intron9a in hypoxic *CTCF* knockdown MCF7 cells **N)** qPCR analysis showing decreased 9a/9b ratio in hypoxic *CTCF* knockdown HCC1806 cells **O)** CTCF-ChIP-qPCR showing decreased occupancy of CTCF at intron9a in hypoxic *CTCF* knockdown HCC1806 cells **P)** RNA PolII-ChIP-qPCR showing decreased occupancy of RNA PolII at intron9a in hypoxic *CTCF* knockdown HCC1806 cells **Q)** ATF3-ChIP-qPCR showing increased occupancy of ATF3 at intron9a in hypoxic HCC1806 cells **R)** MeDIP-qPCR depicting increased methylation at the intron9a under hypoxia in *ATF3* knockdown HCC1806 cells. Error bars show mean values $\pm$ SD (n=3, unless otherwise specified) as calculated using two-tailed Student's t-test, unless otherwise specified, *$P \leq 0.05$, **$P \leq 0.01$, ***$P \leq 0.001$, ns = not significant.

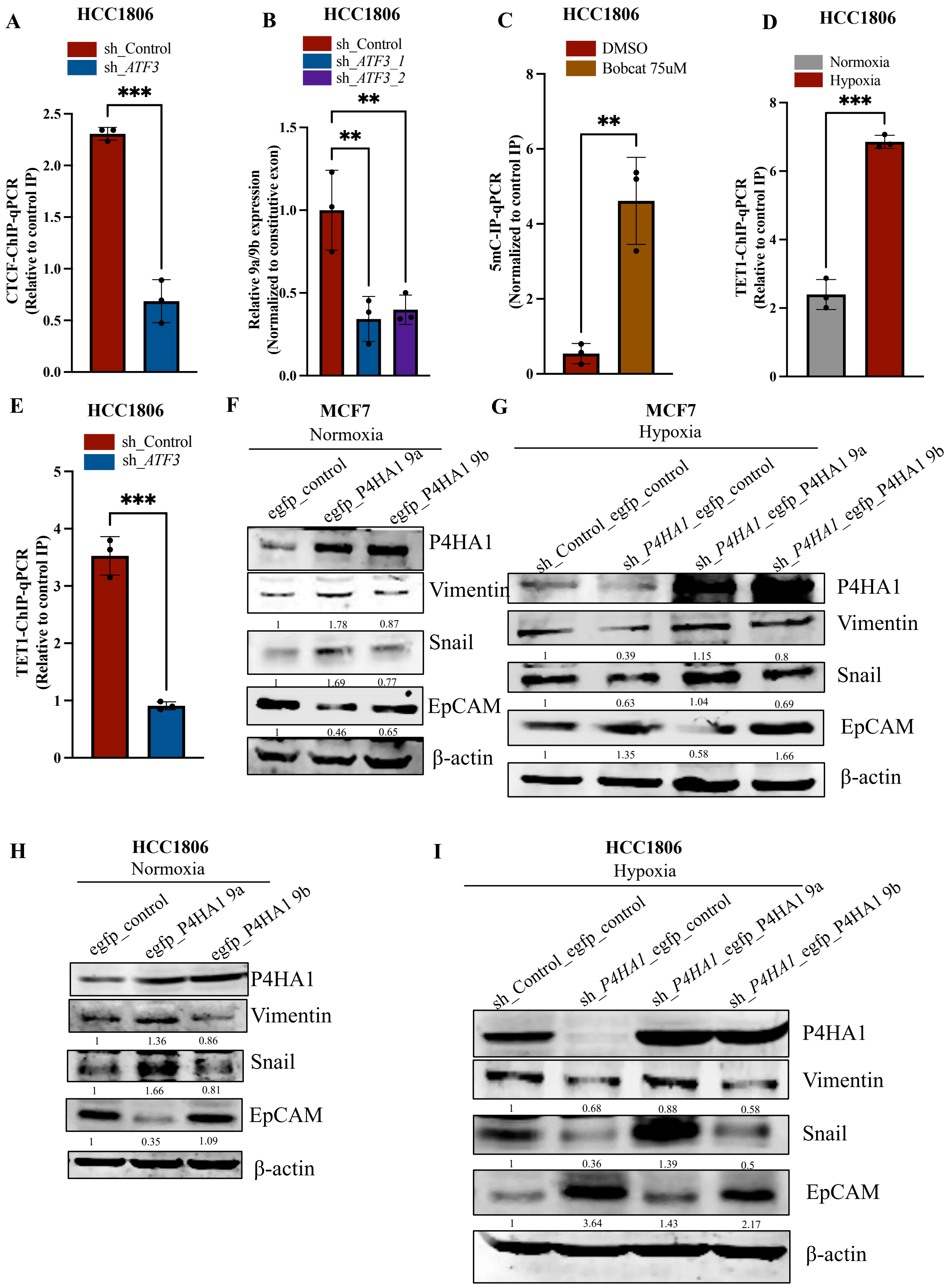

A
HCC1806
sh_Control
sh_ATF3
***
CTCF-ChIP-qPCR (Relative to control IP)
B
HCC1806
sh_Control
sh_ATF3_1
sh_ATF3_2
Relative 9a/9b expression (Normalized to constitutive exon)
C
HCC1806
DMSO
Bobcat 75uM
5mC-IP-qPCR (Normalized to control IP)
D
HCC1806
Normoxia
Hypoxia
TET1-ChIP-qPCR (Relative to control IP)
E
HCC1806
sh_Control
sh_ATF3
TET1-ChIP-qPCR (Relative to control IP)
F
MCF7
Normoxia
egfp_control
egfp_P4HA1 9a
egfp_P4HA1 9b
P4HA1
Vimentin
Snail
EpCAM
β-actin
G
MCF7
Hypoxia
sh_Control_egfp_control
sh_P4HA1_egfp_control
sh_P4HA1_egfp_P4HA1 9a
sh_P4HA1_egfp_P4HA1 9b
H
HCC1806
Normoxia
I
HCC1806
Hypoxia

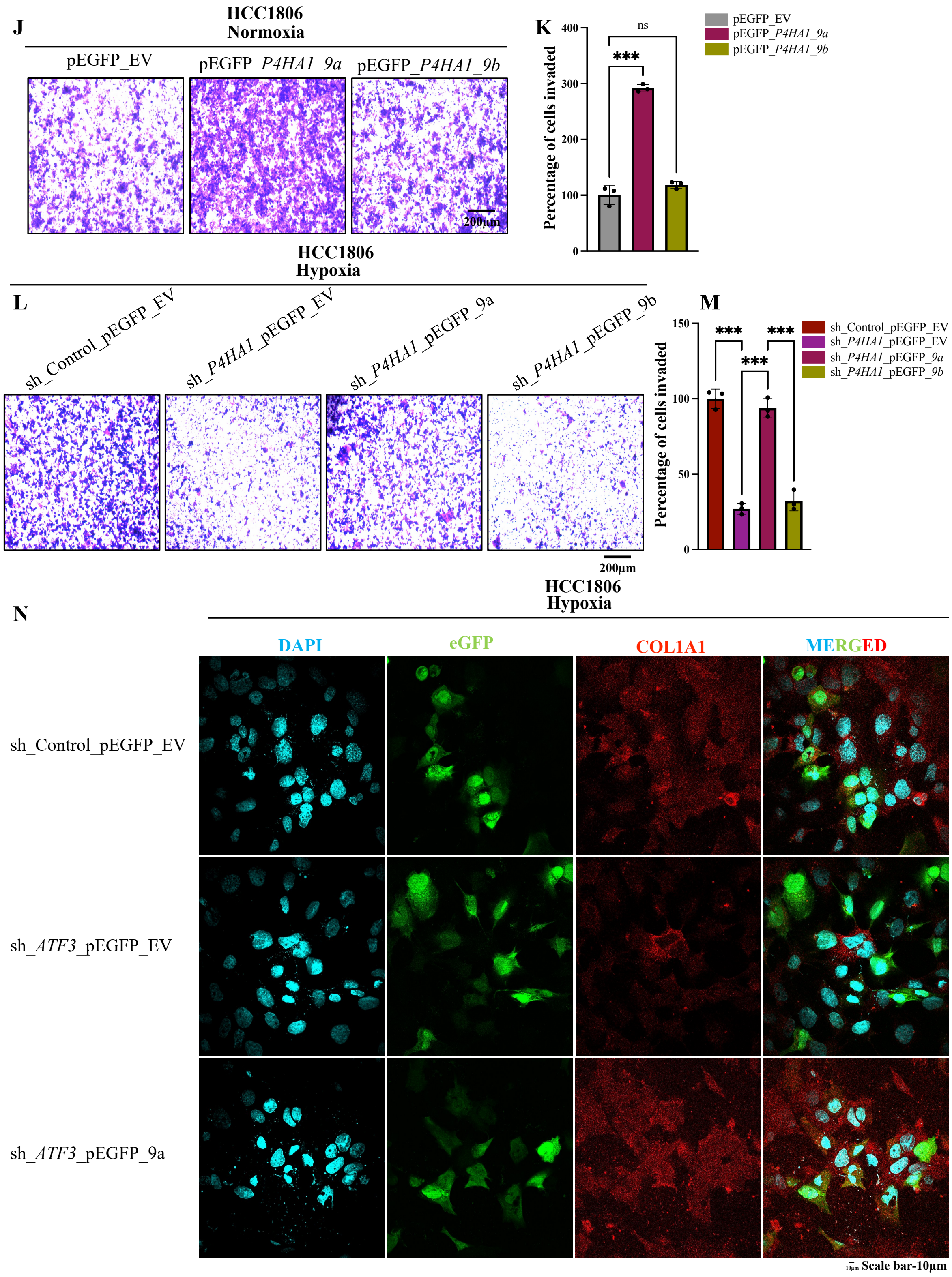
J
HCC1806
Normoxia
pEGFP_EV
pEGFP_P4HA1_9a
pEGFP_P4HA1_9b
200μm
K
Percentage of cells invaded
400
300
200
100
0
ns
***
pEGFP_EV
pEGFP_P4HA1_9a
pEGFP_P4HA1_9b
HCC1806
Hypoxia
L
sh_Control_pEGFP_EV
sh_P4HA1_pEGFP_EV
sh_P4HA1_pEGFP_9a
sh_P4HA1_pEGFP_9b
200μm
M
Percentage of cells invaded
150
100
50
0
***
***
***
sh_Control_pEGFP_EV
sh_P4HA1_pEGFP_EV
sh_P4HA1_pEGFP_9a
sh_P4HA1_pEGFP_9b
N
HCC1806
Hypoxia
DAPI
eGFP
COL1A1
MERGED
sh_Control_pEGFP_EV
sh_ATF3_pEGFP_EV
sh_ATF3_pEGFP_9a
Scale bar-10μm

O

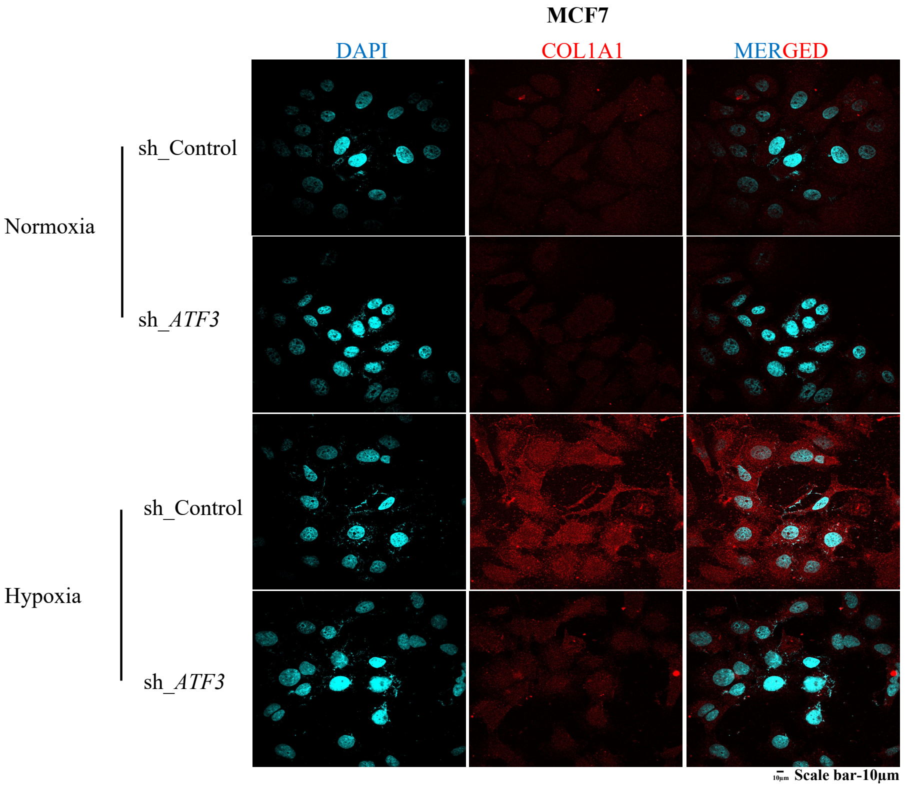
MCF7
DAPI
COL1A1
MERGED
sh_Control
Normoxia
sh_ATF3
sh_Control
Hypoxia
sh_ATF3
Scale bar-10µm

P

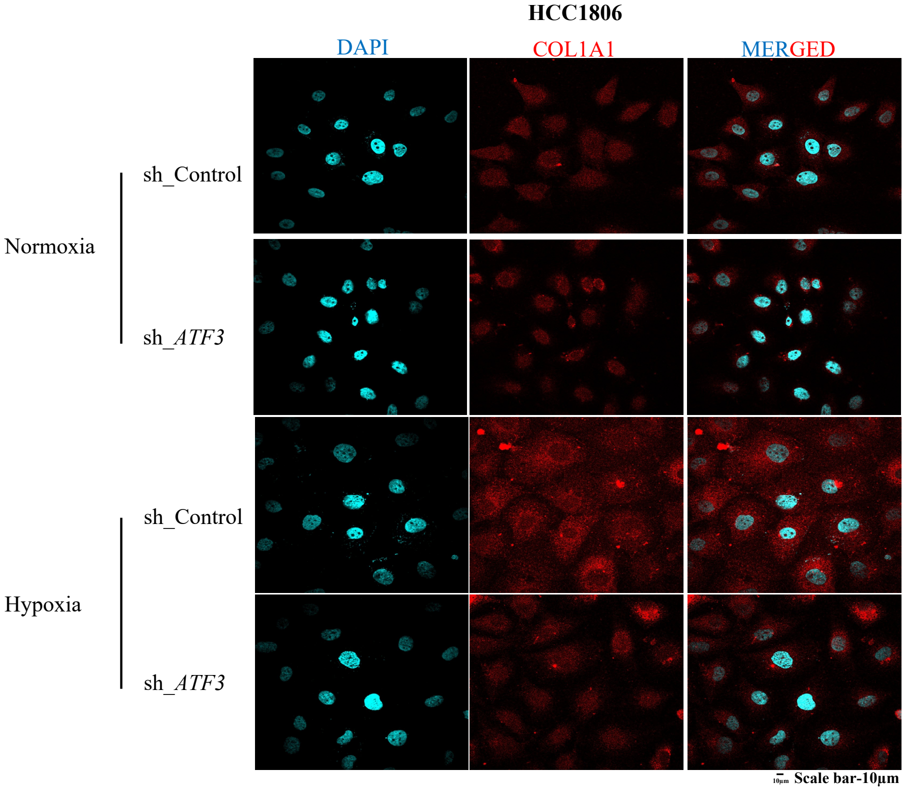
HCC1806
DAPI
COL1A1
MERGED
sh_Control
Normoxia
sh_ATF3
sh_Control
Hypoxia
sh_ATF3
Scale bar-10µm

Q

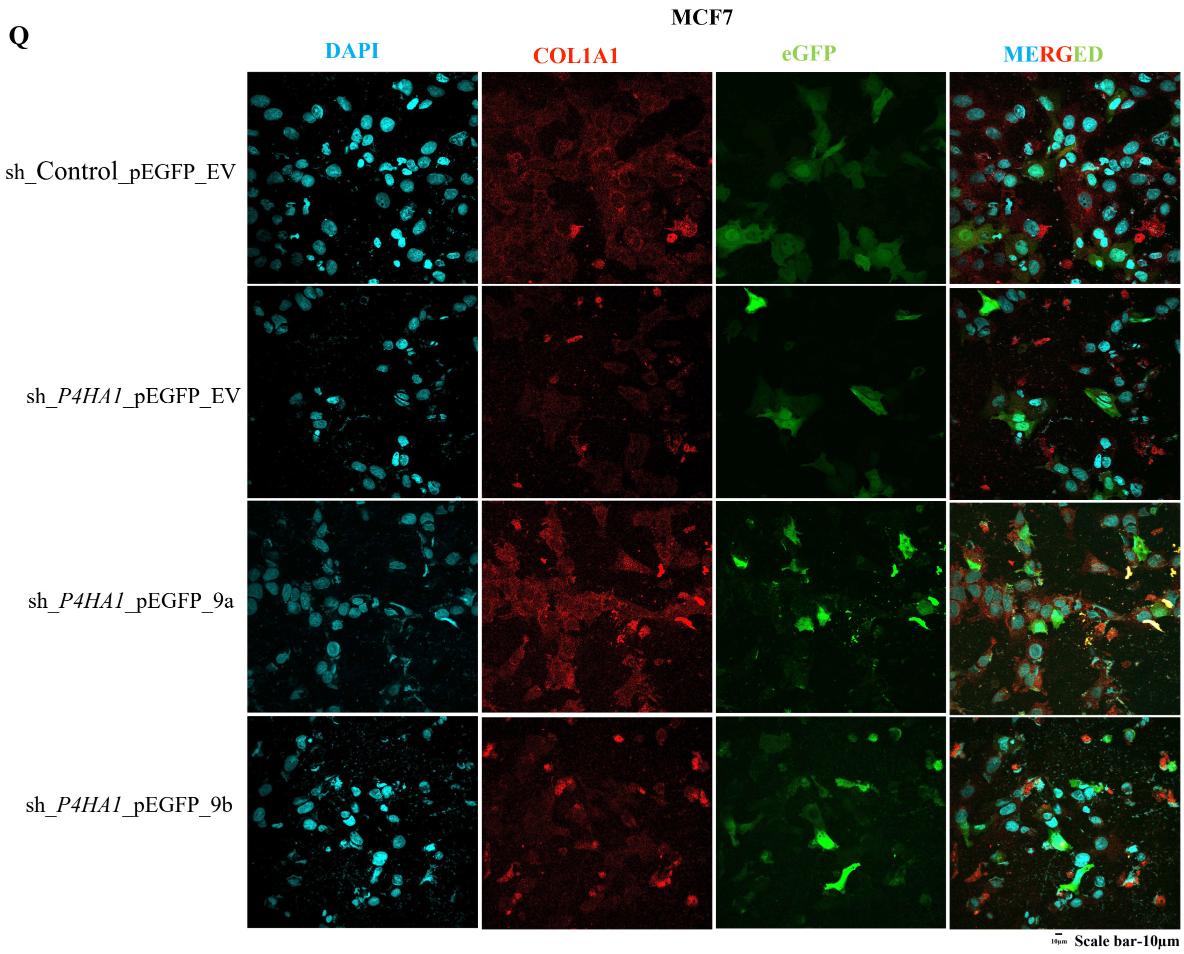

Figure S6. Mechanism of *P4HA1* splicing under hypoxic condition and functional roles of *P4HA1* isoforms in Collagen deposition. A) CTCF-ChIP-qPCR showing decreased occupancy of CTCF at intron9a in hypoxic *ATF3* knockdown HCC1806 cells **B)** qPCR analysis showing decreased 9a/9b ratio in hypoxic *ATF3* knockdown HCC1806 cells **C)** MeDIP-qPCR showing increased methylation at the intron9a upon Bobcat treatment in hypoxic HCC1806 cells **D)** TET1-ChIP-qPCR showing increased occupancy of TET1 at intron9a in hypoxic HCC1806 cells **E)** TET1-ChIP-qPCR showing decreased occupancy of TET1 at intron9a in hypoxic *ATF3* knockdown HCC1806 cells **F)** Immunoblot showing expression of Vimentin, SNAIL and EpCAM in MCF7 cells overexpressing *P4HA1* 9a or 9b isoform as compared to the control cells in normoxic conditions **G)** Immunoblot showing expression of Vimentin, SNAIL and EpCAM in *P4HA1* knockdown MCF7 cells overexpressing *P4HA1* 9a or 9b isoform as compared to the control *P4HA1* knockdown cells as well as the control cells

transfected with a non-targeting shRNA in hypoxic conditions **H)** Immunoblot showing expression of Vimentin, SNAIL and EpCAM in HCC1806 cells overexpressing *P4HA1* 9a or 9b isoform as compared to the control cells in normoxic conditions **I)** Immunoblot showing expression of Vimentin, SNAIL and EpCAM in *P4HA1* knockdown HCC1806 cells overexpressing *P4HA1* 9a or 9b isoform as compared to the control *P4HA1* knockdown cells as well as the control cells transfected with a non-targeting shRNA in hypoxic conditions **J)** Invasion assay and **K)** its quantification in control and *P4HA1 9a* or *9b* isoform overexpressing HCC1806 cells under normoxic condition **L)** Invasion assay and **M)** its quantification in *P4HA1* knockdown HCC1806 cells rescued by overexpressing *P4HA1* 9a or 9b isoform as compared to the control *P4HA1* knockdown cells as well as the control cells transfected with a non-targeting shRNA in hypoxic conditions **N)** Immunostaining of COL1A1 in *ATF3* knockdown HCC1806 cells which is then rescued by *P4HA1 9a* overexpression (DAPI staining is done to show the nucleus and overexpression of the *P4HA1* isoforms is confirmed by GFP, scale bars, 10 μm) **O)** Immunostaining of COL1A1 in *ATF3* knockdown MCF7 and **P)** HCC1806 cells in normoxic and hypoxic conditions (DAPI staining is done to show the nucleus, scale bars, 10 μm) **Q)** Immunostaining of COL1A1 in *P4HA1* knockdown MCF7 cells rescued by overexpressing *P4HA1* 9a or 9b isoform as compared to the control *P4HA1* knockdown cells as well as the control cells transfected with a non-targeting shRNA in hypoxic conditions (DAPI staining is done to show the nucleus and overexpression of the *P4HA1* isoforms is confirmed by GFP, scale bars, 10 μm). Error bars show mean values $\pm$ SD (n=3, unless otherwise specified) as calculated using two-tailed Student's t-test, unless otherwise specified, *$P\leq0.05$, **$P\leq0.01$, ***$P\leq0.001$, ns = not significant.

A

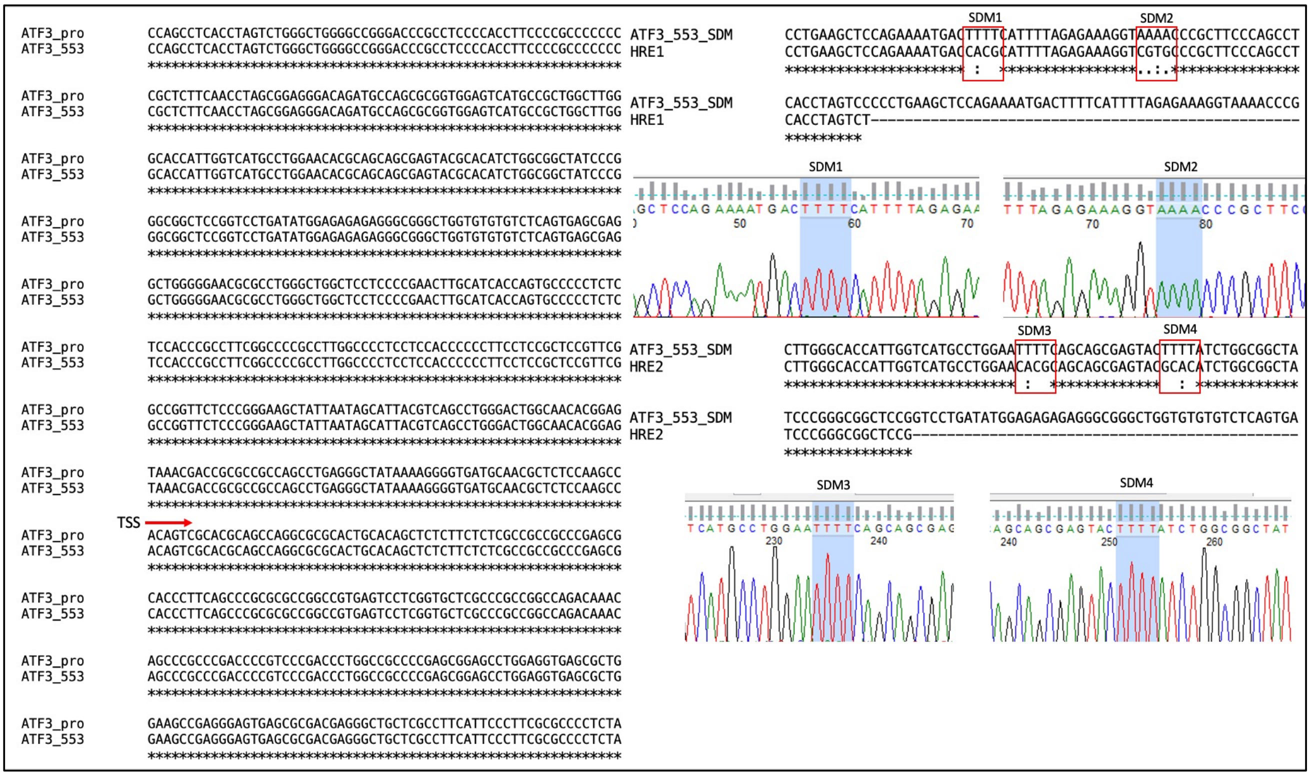

ATF3_pro CCAGCCTCACCTAGTCTGGGCTGGGGCCGGGACCCGCCTCCCCACCTTCCCCGCCCCCCC
ATF3_553 CCAGCCTCACCTAGTCTGGGCTGGGGCCGGGACCCGCCTCCCCACCTTCCCCGCCCCCCC
****
ATF3_pro CGCTCTTCAACCTAGCGGAGGGACAGATGCCAGCGCGGTGGAGTCATGCCGCTGGCTTGG
ATF3_553 CGCTCTTCAACCTAGCGGAGGGACAGATGCCAGCGCGGTGGAGTCATGCCGCTGGCTTGG
****
ATF3_pro GCACCATTGGTCATGCCTGGAACACGCAGCAGCGAGTACGCACATCTGGCGGCTATCCCG
ATF3_553 GCACCATTGGTCATGCCTGGAACACGCAGCAGCGAGTACGCACATCTGGCGGCTATCCCG
****
ATF3_pro GGCGGCTCCGGTCCTGATATGGAGAGAGAGGGCGGGCTGGTGTGTGTCTCAGTGAGCGAG
ATF3_553 GGCGGCTCCGGTCCTGATATGGAGAGAGAGGGCGGGCTGGTGTGTGTCTCAGTGAGCGAG
****
ATF3_pro GCTGGGGGAACGCGCCTGGGCTGGCTCCTCCCCGAACTTGCATCACCAGTGCCCCCTCTC
ATF3_553 GCTGGGGGAACGCGCCTGGGCTGGCTCCTCCCCGAACTTGCATCACCAGTGCCCCCTCTC
****
ATF3_pro TCCACCCGCCTTCGGCCCCGCCTTGGCCCCTCCTCCACCCCCCTTCCTCCGCTCCGTTCG
ATF3_553 TCCACCCGCCTTCGGCCCCGCCTTGGCCCCTCCTCCACCCCCCTTCCTCCGCTCCGTTCG
****
ATF3_pro GCCGGTTCTCCCGGGAAGCTATTAATAGCATTACGTCAGCCTGGGACTGGCAACACGGAG
ATF3_553 GCCGGTTCTCCCGGGAAGCTATTAATAGCATTACGTCAGCCTGGGACTGGCAACACGGAG
****
ATF3_pro TAAACGACCGCGCCGCCAGCCTGAGGGCTATAAAAGGGGTGATGCAACGCTCTCCAAGCC
ATF3_553 TAAACGACCGCGCCGCCAGCCTGAGGGCTATAAAAGGGGTGATGCAACGCTCTCCAAGCC
****
TSS
ATF3_pro ACAGTCGCACGCAGCCAGGCGCGCACTGCACAGCTCTCTTCTCTCGCCGCCGCCCGAGCG
ATF3_553 ACAGTCGCACGCAGCCAGGCGCGCACTGCACAGCTCTCTTCTCTCGCCGCCGCCCGAGCG
****
ATF3_pro CACCCTTCAGCCCGCGCGCCGGCCGTGAGTCCTCGGTGCTCGCCCGCCGGCCAGACAAAC
ATF3_553 CACCCTTCAGCCCGCGCGCCGGCCGTGAGTCCTCGGTGCTCGCCCGCCGGCCAGACAAAC
****
ATF3_pro AGCCCGCCCGACCCCGTCCCGACCCTGGCCGCCCCGAGCGGAGCCTGGAGGTGAGCGCTG
ATF3_553 AGCCCGCCCGACCCCGTCCCGACCCTGGCCGCCCCGAGCGGAGCCTGGAGGTGAGCGCTG
****
ATF3_pro GAAGCCGAGGGAGTGAGCGCGACGAGGGCTGCTCGCCTTCATTCCCTTCGCGCCCCTCTA
ATF3_553 GAAGCCGAGGGAGTGAGCGCGACGAGGGCTGCTCGCCTTCATTCCCTTCGCGCCCCTCTA
****
SDM1
SDM2
ATF3_553_SDM CCTGAAGCTCCAGAAAATGACTTTTCATTTTAGAGAAAGGTAAAACCCGCTTCCCAGCCT
HRE1 CCTGAAGCTCCAGAAAATGACCACGCATTTTAGAGAAAGGTCGTGCCCGCTTCCCAGCCT
********************* : ****************..:.***************
ATF3_553_SDM CACCTAGTCCCCCTGAAGCTCCAGAAAATGACTTTTCATTTTAGAGAAAGGTAAAACCCG
HRE1 CACCTAGTCT--
****
SDM1
GCTCCAGAAAATGACTTTTCATTTTAGAGAA
50 60 70
SDM2
TTTAGAGAAAGGTAAAACCCGCTTC
70 80
SDM3
SDM4
ATF3_553_SDM CTTGGGCACCATTGGTCATGCCTGGAATTTTCAGCAGCGAGTACTTTTATCTGGCGGCTA
HRE2 CTTGGGCACCATTGGTCATGCCTGGAACACGCAGCAGCGAGTACGCACATCTGGCGGCTA
*************************** : ************* : ************
ATF3_553_SDM TCCCGGGCGGCTCCGGTCCTGATATGGAGAGAGAGGGCGGGCTGGTGTGTGTCTCAGTGA
HRE2 TCCCGGGCGGCTCCG---
****
SDM3
TCATGCCTGGAATTTTCAGCAGCGAG
230 240
SDM4
AGCAGCGAGTACTTTTATCTGGCGGCTAT
240 250 260

B

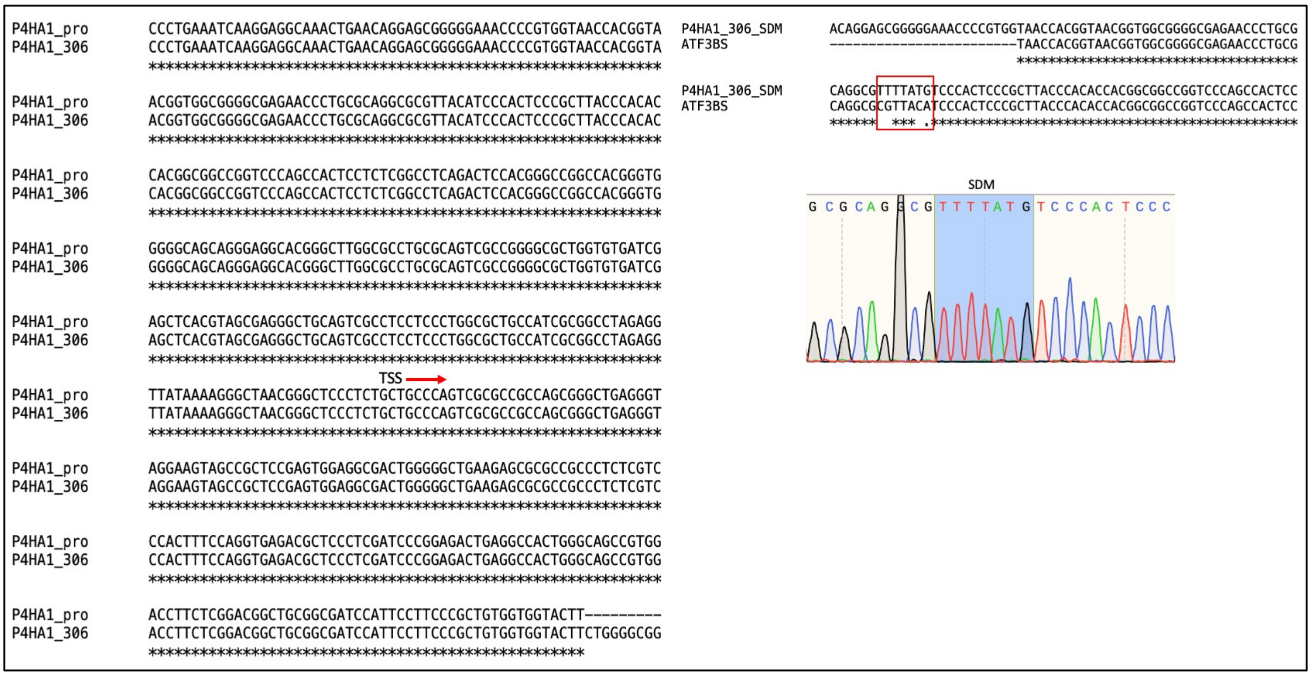

P4HA1_pro CCCTGAAATCAAGGAGGCAAACTGAACAGGAGCGGGGGAAACCCCGTGGTAACCACGGTA
P4HA1_306 CCCTGAAATCAAGGAGGCAAACTGAACAGGAGCGGGGGAAACCCCGTGGTAACCACGGTA
****
P4HA1_pro ACGGTGGCGGGGCGAGAACCCTGCGCAGGCGCGTTACATCCCACTCCCGCTTACCCACAC
P4HA1_306 ACGGTGGCGGGGCGAGAACCCTGCGCAGGCGCGTTACATCCCACTCCCGCTTACCCACAC
****
P4HA1_pro CACGGCGGCCGGTCCCAGCCACTCCTCTCGGCCTCAGACTCCACGGGCCGGCCACGGGTG
P4HA1_306 CACGGCGGCCGGTCCCAGCCACTCCTCTCGGCCTCAGACTCCACGGGCCGGCCACGGGTG
****
P4HA1_pro GGGGCAGCAGGGAGGCACGGGCTTGGCGCCTGCGCAGTCGCCGGGGCGCTGGTGTGATCG
P4HA1_306 GGGGCAGCAGGGAGGCACGGGCTTGGCGCCTGCGCAGTCGCCGGGGCGCTGGTGTGATCG
****
P4HA1_pro AGCTCACGTAGCGAGGGCTGCAGTCGCCTCCTCCCTGGCGCTGCCATCGCGGCCTAGAGG
P4HA1_306 AGCTCACGTAGCGAGGGCTGCAGTCGCCTCCTCCCTGGCGCTGCCATCGCGGCCTAGAGG
****
TSS
P4HA1_pro TTATAAAAGGGCTAACGGGCTCCCTCTGCTGCCCAGTCGCGCCGCCAGCGGGCTGAGGGT
P4HA1_306 TTATAAAAGGGCTAACGGGCTCCCTCTGCTGCCCAGTCGCGCCGCCAGCGGGCTGAGGGT
****
P4HA1_pro AGGAAGTAGCCGCTCCGAGTGGAGGCGACTGGGGGCTGAAGAGCGCGCCGCCCTCTCGTC
P4HA1_306 AGGAAGTAGCCGCTCCGAGTGGAGGCGACTGGGGGCTGAAGAGCGCGCCGCCCTCTCGTC
****
P4HA1_pro CCACTTTCCAGGTGAGACGCTCCCTCGATCCCGGAGACTGAGGCCACTGGGCAGCCGTGG
P4HA1_306 CCACTTTCCAGGTGAGACGCTCCCTCGATCCCGGAGACTGAGGCCACTGGGCAGCCGTGG
****
P4HA1_pro ACCTTCTCGGACGGCTGCGGCGATCCATTCCTTCCCGCTGTGGTGGTACTT---------
P4HA1_306 ACCTTCTCGGACGGCTGCGGCGATCCATTCCTTCCCGCTGTGGTGGTACTTCTGGGGCGG
****
P4HA1_306_SDM ACAGGAGCGGGGGAAACCCCGTGGTAACCACGGTAACGGTGGCGGGGCGAGAACCCTGCG
ATF3BS ------------------------TAACCACGGTAACGGTGGCGGGGCGAGAACCCTGCG
****
P4HA1_306_SDM CAGGCGTTTTATGTCCCACTCCCGCTTACCCACACCACGGCGGCCGGTCCCAGCCACTCC
ATF3BS CAGGCGCGTTACATCCCACTCCCGCTTACCCACACCACGGCGGCCGGTCCCAGCCACTCC
****** *** .***
SDM
GCGCAGGCGTTTTATGTCCCACTCCC

C

```
ATF3_cds          ------------------------------------------------------------
ATF3_OEvector     CTCGAAATTAACCCTCACTAAAGGGAACAAAAGCTGGAGCTCCACCGCGGTGGCGGCCGC


ATF3_cds          ------------------------------------------------------------
ATF3_OEvector     CACCATGGATTACAAGGATGACGACGATAAGGACTATAAGGACGATGATGACAAGGACTA


ATF3_cds          ----------------------------------ATGATGCTTCAACACCCAGGCCAGGT
ATF3_OEvector     CAAAGATGATGACGATAAAGCCCGGGCGGGATCCATGATGCTTCAACACCCAGGCCAGGT
                                                    **************************

ATF3_cds          CTCTGCCTCGGAAGTGAGTGCTTCTGCCATCGTCCCCTGCCTGTCCCCTCCTGGGTCACT
ATF3_OEvector     CTCTGCCTCGGAAGTGAGTGCTTCTGCCATCGTCCCCTGCCTGTCCCCTCCTGGGTCACT
                  ************************************************************

ATF3_cds          GGTGTTTGAGGATTTTGCTAACCTGACGCCCTTTGTCAAGGAAGAGCTGAGGTTTGCCAT
ATF3_OEvector     GGTGTTTGAGGATTTTGCTAACCTGACGCCCTTTGTCAAGGAAGAGCTGAGGTTTGCCAT
                  ************************************************************

ATF3_cds          CCAGAACAAGCACCTCTGCCACCGGATGTCCTCTGCGCTGGAATCAGTCACTGTCAGCGA
ATF3_OEvector     CCAGAACAAGCACCTCTGCCACCGGATGTCCTCTGCGCTGGAATCAGTCACTGTCAGCGA
                  ************************************************************

ATF3_cds          CAGACCCCTCGGGGTGTCCATCACAAAAGCCGAGGTAGCCCCTGAAGAAGATGAAAGGAA
ATF3_OEvector     CAGACCCCTCGGGGTGTCCATCACAAAAGCCGAGGTAGCCCCTGAAGAAGATGAAAGGAA
                  ************************************************************

ATF3_cds          AAAGAGGCGACGAGAAAGAAATAAGATTGCAGCTGCAAAGTGCCGAAACAAGAAGAAGGA
ATF3_OEvector     AAAGAGGCGACGAGAAAGAAATAAGATTGCAGCTGCAAAGTGCCGAAACAAGAAGAAGGA
                  ************************************************************

ATF3_cds          GAAGACGGAGTGCCTGCAGAAAGAGTCGGAGAAGCTGGAAAGTGTGAATGCTGAACTGAA
ATF3_OEvector     GAAGACGGAGTGCCTGCAGAAAGAGTCGGAGAAGCTGGAAAGTGTGAATGCTGAACTGAA
                  ************************************************************

ATF3_cds          GGCTCAGATTGAGGAGCTCAAGAACGAGAAGCAGCATTTGATATACATGCTCAACCTTCA
ATF3_OEvector     GGCTCAGATTGAGGAGCTCAAGAACGAGAAGCAGCATTTGATATACATGCTCAACCTTCA
                  ************************************************************

ATF3_cds          TCGGCCCACGTGTATTGTCCGGGCTCAGAATGGGAGGACTCCAGAAGATGAGAGAAACCT
ATF3_OEvector     TCGGCCCACGTGTATTGTCCGGGCTCAGAATGGGAGGACTCCAGAAGATGAGAGAAACCT
                  ************************************************************

ATF3_cds          CTTTATCCAACAGATAAAAGAAGGAACATTGCAGAGCTAA--------------------
ATF3_OEvector     CTTTATCCAACAGATAAAAGAAGGAACATTGCAGAGCTAACTCGAGGGGGGGCCCGGTAC
                  ****************************************

ATF3_cds          ------------------------------------------------------------
ATF3_OEvector     CTTAATTAATTAAGGTACCAGGTAAGTGTACCCAATTCGCCCTATAGTGAGTCGTATTAC


ATF3_cds          ------------------------------------------------------------
ATF3_OEvector     AATTCACTCGATCGGCTCGCTGATCAGCCTCGACTGTGCCTTCTAGTTGCCAGCCATCTG
```

D

```
P4HA1_204_9a__cds    ------------------------------------------------------------
P4HA1_9a_OEvector    CACCATGGATTACAAGGATGACGACGATAAGGACTATAAGGACGATGATGACAAGGACTA

P4HA1_204_9a__cds    ----------------------------------ATGATCTGGTATATATTAATTATAGG
P4HA1_9a_OEvector    CAAAGATGATGACGATAAAGCCCGGGCGGGATCCATGATCTGGTATATATTAATTATAGG
                                                       **************************

P4HA1_204_9a__cds    AATTCTGCTTCCCCAGTCTTTGGCTCATCCAGGCTTTTTTACTTCAATTGGTCAGATGAC
P4HA1_9a_OEvector    AATTCTGCTTCCCCAGTCTTTGGCTCATCCAGGCTTTTTTACTTCAATTGGTCAGATGAC
                     ************************************************************

P4HA1_204_9a__cds    TGATTTGATCCATACTGAGAAAGATCTGGTGACTTCTCTGAAAGATTATATTAAGGCAGA
P4HA1_9a_OEvector    TGATTTGATCCATACTGAGAAAGATCTGGTGACTTCTCTGAAAGATTATATTAAGGCAGA
                     ************************************************************

P4HA1_204_9a__cds    AGAGGACAAGTTAGAACAAATAAAAAAATGGGCAGAGAAGTTAGATCGGCTAACTAGTAC
P4HA1_9a_OEvector    AGAGGACAAGTTAGAACAAATAAAAAAATGGGCAGAGAAGTTAGATCGGCTAACTAGTAC
                     ************************************************************

P4HA1_204_9a__cds    AGCGACAAAAGATCCAGAAGGATTTGTTGGGCATCCAGTAAATGCATTCAAATTAATGAA
P4HA1_9a_OEvector    AGCGACAAAAGATCCAGAAGGATTTGTTGGGCATCCAGTAAATGCATTCAAATTAATGAA
                     ************************************************************

P4HA1_204_9a__cds    ACGTCTGAATACTGAGTGGAGTGAGTTGGAGAATCTGGTCCTTAAGGATATGTCAGATGG
P4HA1_9a_OEvector    ACGTCTGAATACTGAGTGGAGTGAGTTGGAGAATCTGGTCCTTAAGGATATGTCAGATGG
                     ************************************************************

P4HA1_204_9a__cds    CTTTATCTCTAACCTAACCATTCAGAGACAGTACTTTCCTAATGATGAAGATCAGGTTGG
P4HA1_9a_OEvector    CTTTATCTCTAACCTAACCATTCAGAGACAGTACTTTCCTAATGATGAAGATCAGGTTGG
                     ************************************************************

P4HA1_204_9a__cds    GGCAGCCAAAGCTCTGTTACGTCTCCAGGATACCTACAATTTGGATACAGATACCATCTC
P4HA1_9a_OEvector    GGCAGCCAAAGCTCTGTTACGTCTCCAGGATACCTACAATTTGGATACAGATACCATCTC
                     ************************************************************

P4HA1_204_9a__cds    AAAGGGTAATCTTCCAGGAGTGAAACACAAATCTTTTCTAACGGCTGAGGACTGCTTTGA
P4HA1_9a_OEvector    AAAGGGTAATCTTCCAGGAGTGAAACACAAATCTTTTCTAACGGCTGAGGACTGCTTTGA
                     ************************************************************

P4HA1_204_9a__cds    GTTGGGCAAAGTGGCCTATACAGAAGCAGATTATTACCATACGGAACTGTGGATGGAACA
P4HA1_9a_OEvector    GTTGGGCAAAGTGGCCTATACAGAAGCAGATTATTACCATACGGAACTGTGGATGGAACA
                     ************************************************************

P4HA1_204_9a__cds    AGCCCTAAGGCAACTGGATGAAGGCGAGATTTCTACCATAGATAAAGTCTCTGTTCTAGA
P4HA1_9a_OEvector    AGCCCTAAGGCAACTGGATGAAGGCGAGATTTCTACCATAGATAAAGTCTCTGTTCTAGA
                     ************************************************************

P4HA1_204_9a__cds    TTATTTGAGCTATGCGGTATATCAGCAGGGAGACCTGGATAAGGCACTTTTGCTCACAAA
P4HA1_9a_OEvector    TTATTTGAGCTATGCGGTATATCAGCAGGGAGACCTGGATAAGGCACTTTTGCTCACAAA
                     ************************************************************

P4HA1_204_9a__cds    GAAGCTTCTTGAACTAGATCCTGAACATCAGAGAGCTAATGGTAACTTAAAATATTTTGA
P4HA1_9a_OEvector    GAAGCTTCTTGAACTAGATCCTGAACATCAGAGAGCTAATGGTAACTTAAAATATTTTGA
                     ************************************************************

P4HA1_204_9a__cds    GTATATAATGGCTAAAGAAAAAGATGTCAATAAGTCTGCTTCAGATGACCAATCTGATCA
P4HA1_9a_OEvector    GTATATAATGGCTAAAGAAAAAGATGTCAATAAGTCTGCTTCAGATGACCAATCTGATCA
                     ************************************************************

P4HA1_204_9a__cds    GAAAACTACACCAAAGAAAAAAGGGGTTGCTGTGGATTACCTGCCAGAGAGACAGAAGTA
P4HA1_9a_OEvector    GAAAACTACACCAAAGAAAAAAGGGGTTGCTGTGGATTACCTGCCAGAGAGACAGAAGTA
                     ************************************************************

P4HA1_204_9a__cds    CGAAATGCTGTGCCGTGGGGAGGGTATCAAAATGACCCCTCGGAGACAGAAAAAACTCTT
P4HA1_9a_OEvector    CGAAATGCTGTGCCGTGGGGAGGGTATCAAAATGACCCCTCGGAGACAGAAAAAACTCTT
                     ************************************************************

P4HA1_204_9a__cds    TTGCCGCTACCATGATGGAAACCGTAATCCTAAATTTATTCTGGCTCCAGCTAAACAGGA
P4HA1_9a_OEvector    TTGCCGCTACCATGATGGAAACCGTAATCCTAAATTTATTCTGGCTCCAGCTAAACAGGA
                     ************************************************************

P4HA1_204_9a__cds    GGATGAATGGGACAAGCCTCGTATTATTCGCTTCCATGATATTATTTCTGATGCAGAAAT
P4HA1_9a_OEvector    GGATGAATGGGACAAGCCTCGTATTATTCGCTTCCATGATATTATTTCTGATGCAGAAAT
                     ************************************************************

P4HA1_204_9a__cds    TGAAATCGTCAAAGACCTAGCAAAACCAAGGCTGAGGCGAGCCACCATTTCAAACCCAAT
P4HA1_9a_OEvector    TGAAATCGTCAAAGACCTAGCAAAACCAAGGCTGAGGCGAGCCACCATTTCAAACCCAAT
                     ************************************************************ P4HA1_9a

P4HA1_204_9a__cds    AACAGGAGACTTGGAGACGGTACATTACAGAATTAGCAAAAGTGCCTGGCTCTCTGGCTA
P4HA1_9a_OEvector    AACAGGAGACTTGGAGACGGTACATTACAGAATTAGCAAAAGTGCCTGGCTCTCTGGCTA
                     ************************************************************

P4HA1_204_9a__cds    TGAAAATCCTGTGGTGTCTCGAATTAATATGAGAATACAAGATCTAACAGGACTAGATGT
P4HA1_9a_OEvector    TGAAAATCCTGTGGTGTCTCGAATTAATATGAGAATACAAGATCTAACAGGACTAGATGT
                     ************************************************************

P4HA1_204_9a__cds    TTCCACAGCAGAGGAATTACAGGTAGCAAATTATGGAGTTGGAGGACAGTATGAACCCCA
P4HA1_9a_OEvector    TTCCACAGCAGAGGAATTACAGGTAGCAAATTATGGAGTTGGAGGACAGTATGAACCCCA
                     ************************************************************

P4HA1_204_9a__cds    TTTTGACTTTGCACGGAAAGATGAGCCAGATGCTTTCAAAGAGCTGGGGACAGGAAATAG
P4HA1_9a_OEvector    TTTTGACTTTGCACGGAAAGATGAGCCAGATGCTTTCAAAGAGCTGGGGACAGGAAATAG
                     ************************************************************
```

```
P4HA1_204_9a__cds    AATTGCTACATGGCTGTTTTATATGAGTGATGTGTCTGCAGGAGGAGCCACTGTTTTTCC
P4HA1_9a_OEvector    AATTGCTACATGGCTGTTTTATATGAGTGATGTGTCTGCAGGAGGAGCCACTGTTTTTCC
                     ************************************************************

P4HA1_204_9a__cds    TGAAGTTGGAGCTAGTGTTTGGCCCAAAAAAGGAACTGCTGTTTTCTGGTATAATCTGTT
P4HA1_9a_OEvector    TGAAGTTGGAGCTAGTGTTTGGCCCAAAAAAGGAACTGCTGTTTTCTGGTATAATCTGTT
                     ************************************************************

P4HA1_204_9a__cds    TGCCAGTGGAGAAGGAGATTATAGTACACGGCATGCAGCCTGTCCAGTGCTAGTTGGCAA
P4HA1_9a_OEvector    TGCCAGTGGAGAAGGAGATTATAGTACACGGCATGCAGCCTGTCCAGTGCTAGTTGGCAA
                     ************************************************************

P4HA1_204_9a__cds    CAAATGGGTATCCAATAAATGGCTCCATGAACGTGGACAAGAATTTCGAAGACCTTGTAC
P4HA1_9a_OEvector    CAAATGGGTATCCAATAAATGGCTCCATGAACGTGGACAAGAATTTCGAAGACCTTGTAC
                     ************************************************************

P4HA1_204_9a__cds    GTTGTCAGAATTGGAATGA-----------------------------------------
P4HA1_9a_OEvector    GTTGTCAGAATTGGAATGAGGATCCACCGGATCTAGATAACTGATCATAATCAGCCATAC
                     *******************
```

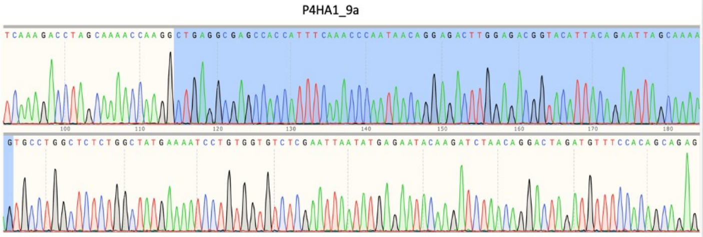

E

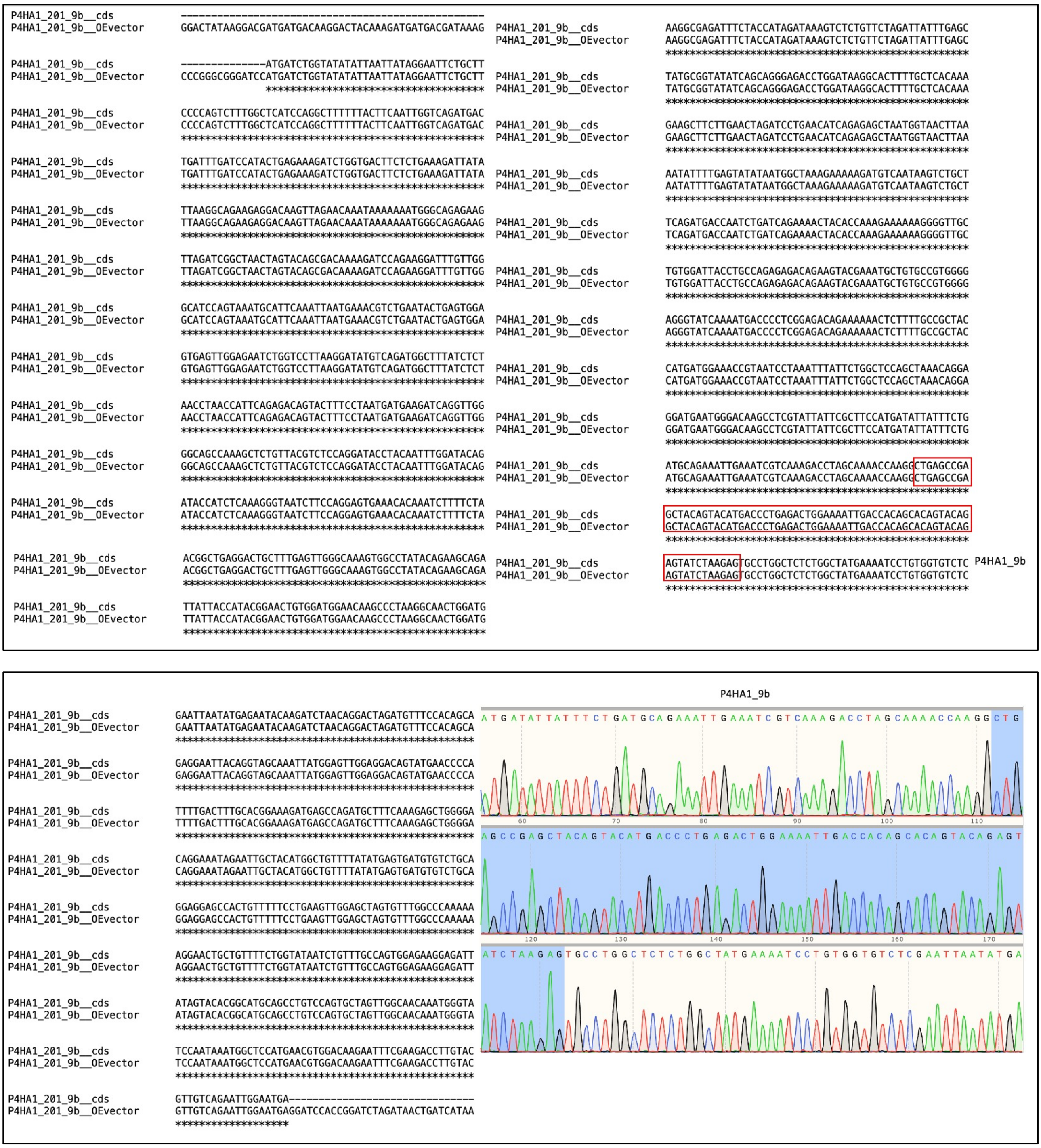

Figure S7. Sanger sequencing for confirming the sequences of A) pGL3_*ATF3*pro luciferase vector **B)** pGL3_*P4HA1*pro luciferase vector (confirmed sequencing results of *ATF3* and *P4HA1* promoter luciferase vectors were obtained after alignment (performed on Clustal Omega Multiple Sequence Alignment tool) with the promoter sequence of these genes

(obtained from Eukaryotic Promoter Database) The TSS has been marked within the sequences. Also, we have checked for the SDM and confirmed the mutated motifs in the SDM vectors, which have been marked within the sequences and shown in the chromatogram as well) **C)** pCMV3tag1A_*ATF3* overexpression vector **D)** egfp_*P4HA1_9a* overexpression vector and **E)** egfp_*P4HA1_9b* overexpression vector (confirmed sequencing results of pCMV3tag1A_*ATF3* and pegfp_*P4HA1* overexpression vectors were obtained after alignment (performed on Clustal Omega Multiple Sequence Alignment tool) with the coding sequences of these genes (obtained from Ensembl). To differentiate between the *P4HA1 9a* and *P4HA1 9b* sequences, we have marked the exons 9a and 9b within the sequences as well as shown in the chromatogram).
