# Supplementary material for: Hypoxia-induced ATF3 escalates breast cancer invasion by increasing collagen deposition via P4HA1
Source: Cell Death Dis. 2025 Feb 27;16(1):142. doi: 10.1038/s41419-025-07461-y (PMC11868403; doi:10.1038/s41419-025-07461-y)

Supplementary file 2- Uncropped images of Western blots.

Figure 1D

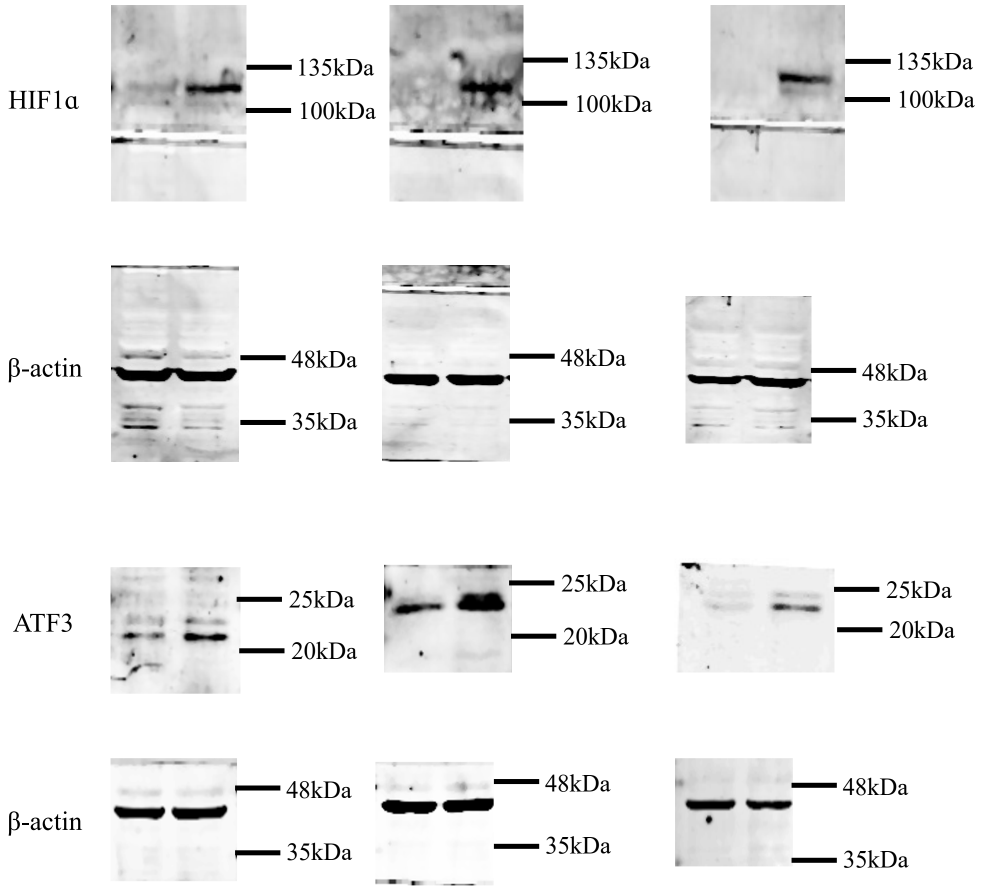

Figure 1I

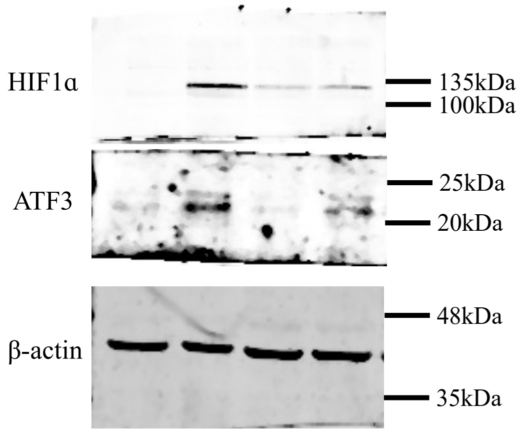

Figure 1J

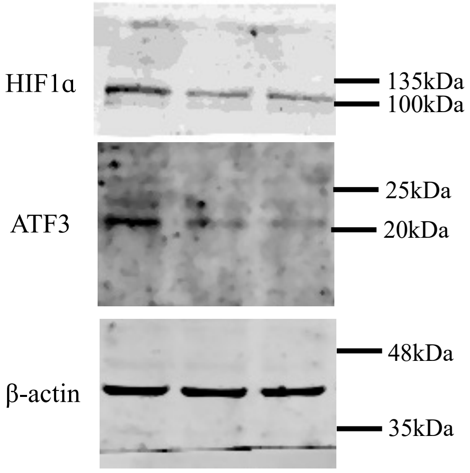

Figure 2E

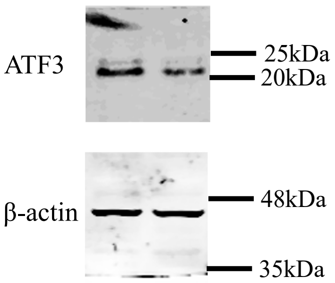

Figure 2G

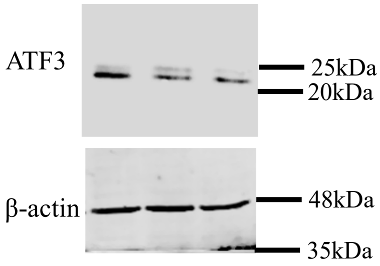

Figure 2H

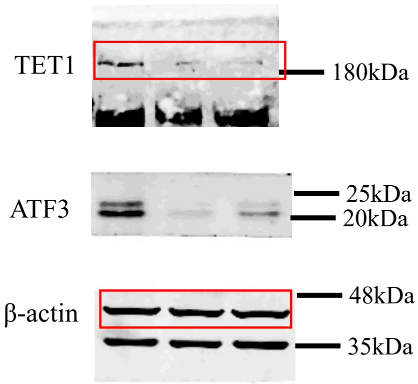

Figure 2J

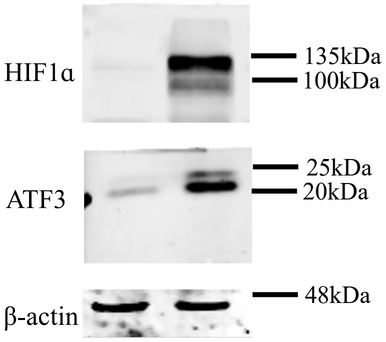

Figure 3F

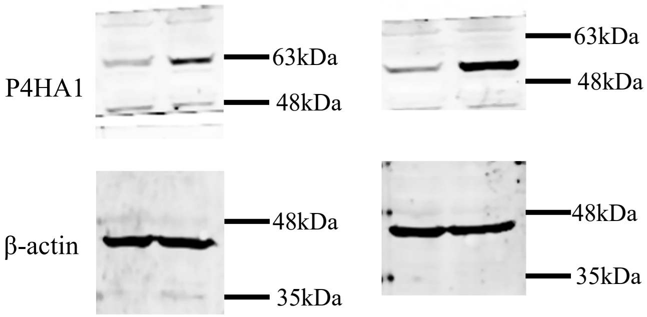

Figure 3J

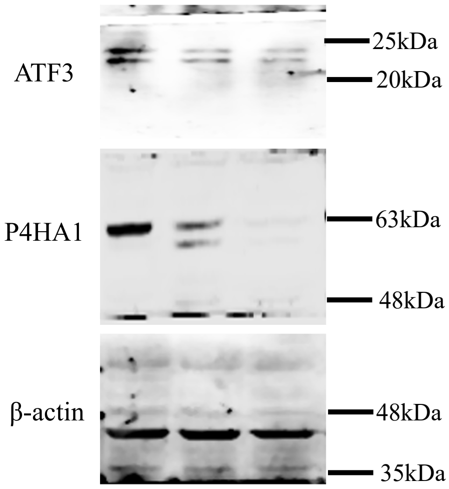

Figure 3K

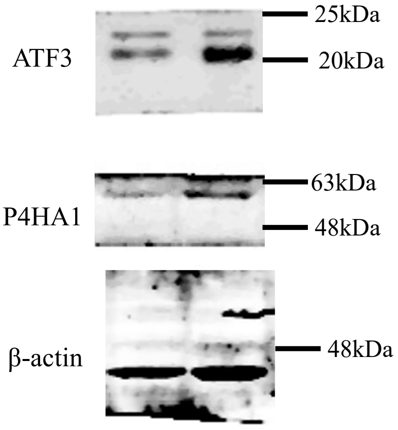

Figure S1H

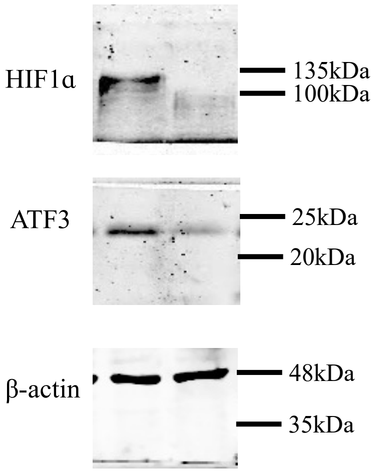

Figure S3E

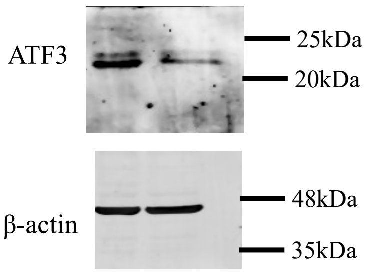

Figure S3G

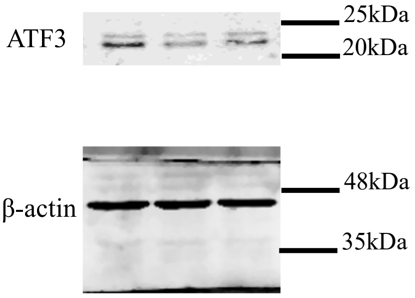

Figure S3H

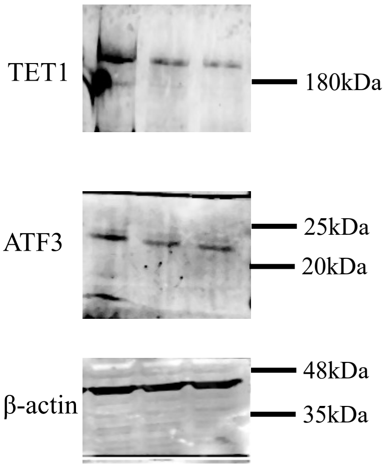

Figure S3I

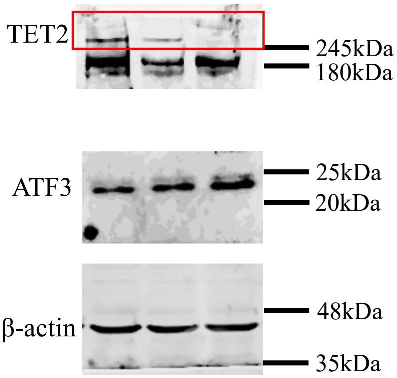

Figure S3J

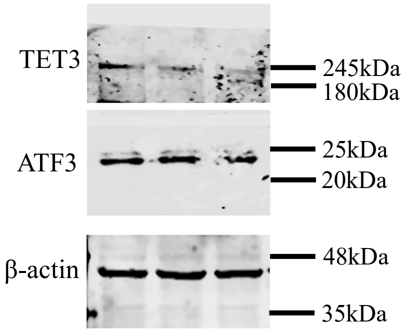

Figure S3L

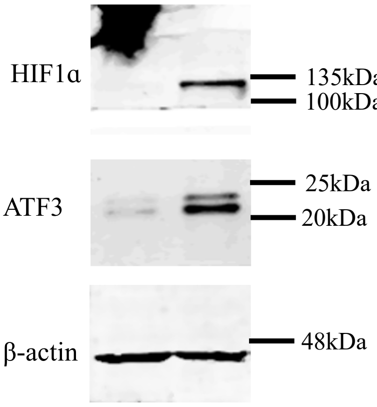

Figure S4B

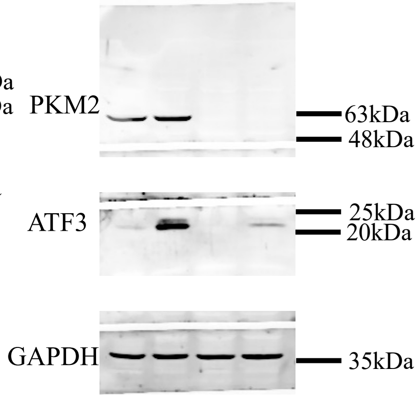

Figure S4C

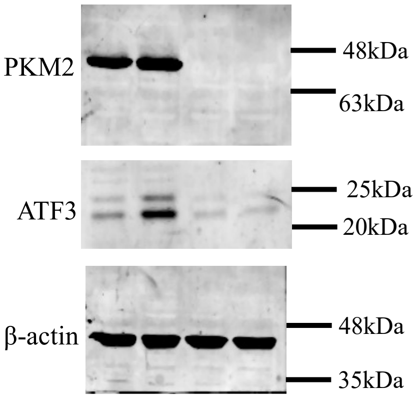

Figure S4K

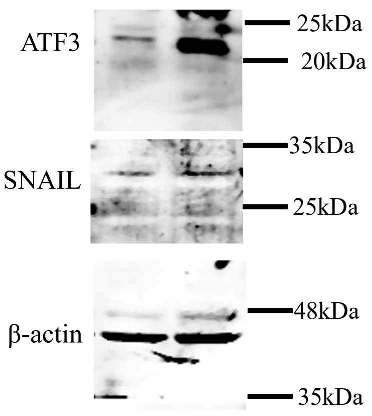

Figure S4N

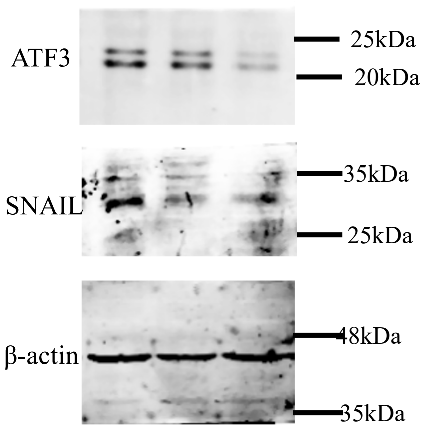

Figure S4O

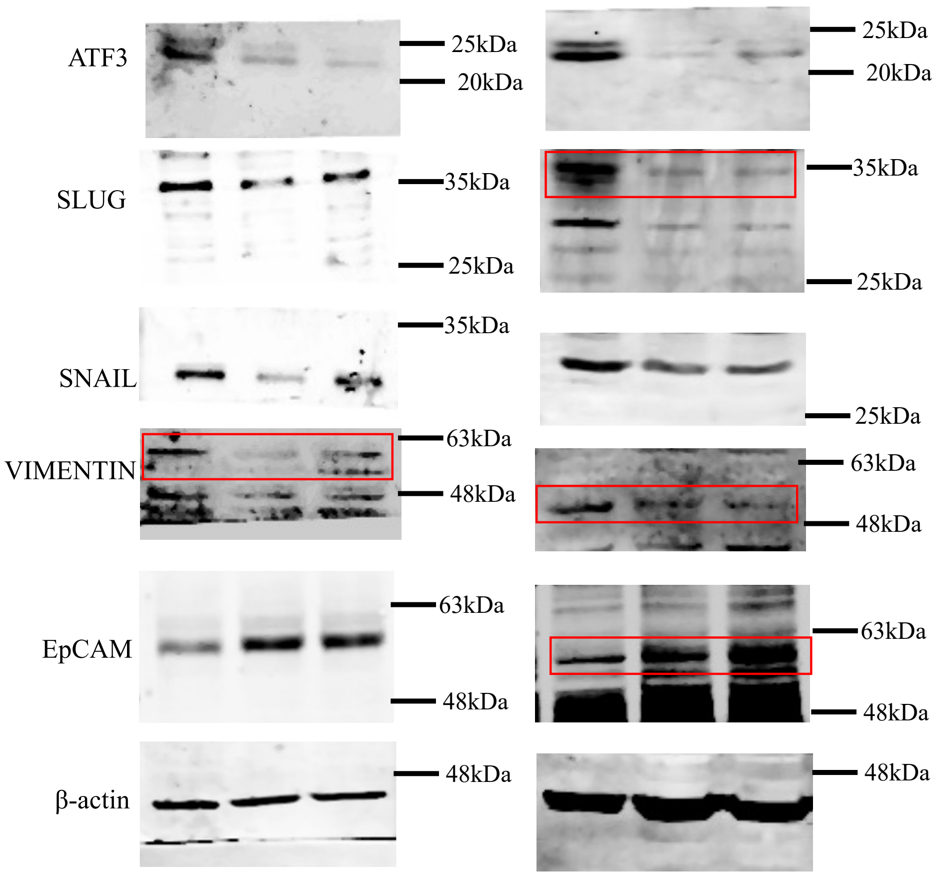

Figure S5C

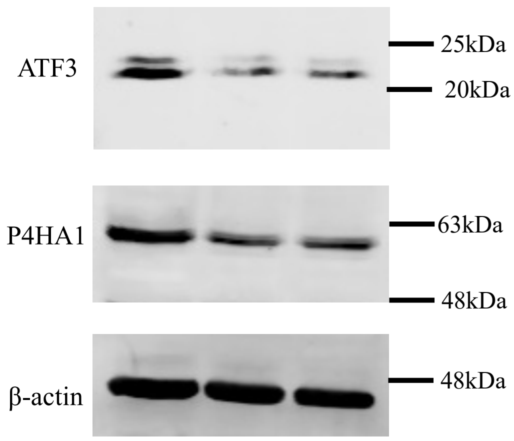

Figure S5D

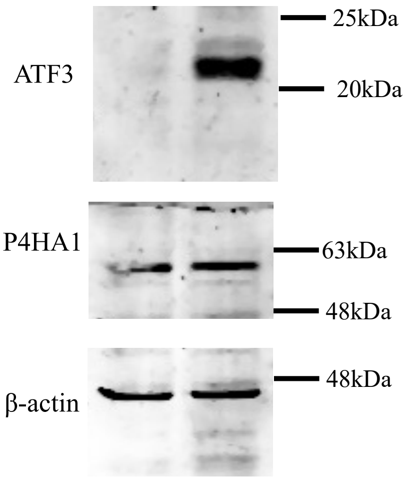

Figure S6F

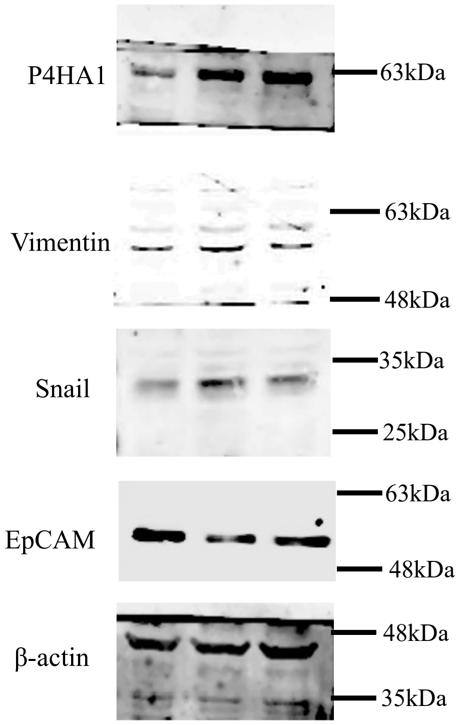

Figure S6G

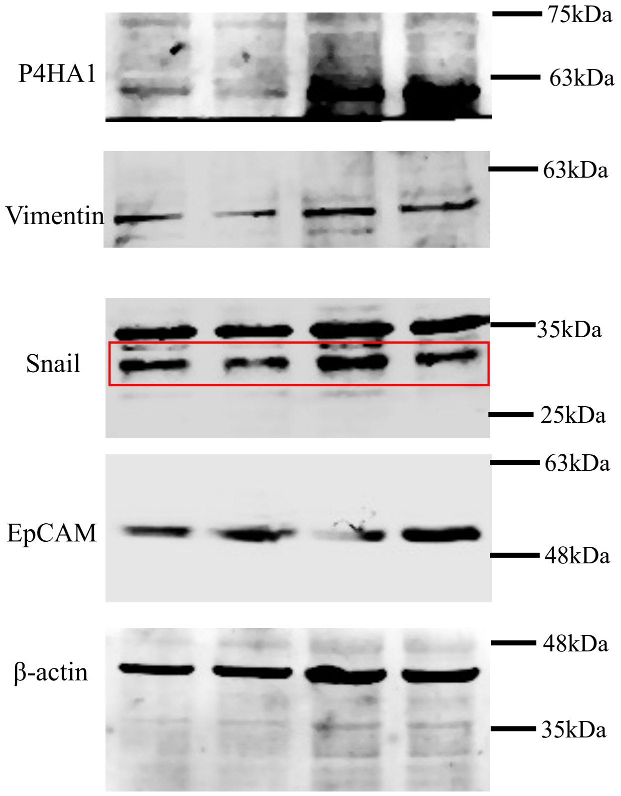

Figure S6H

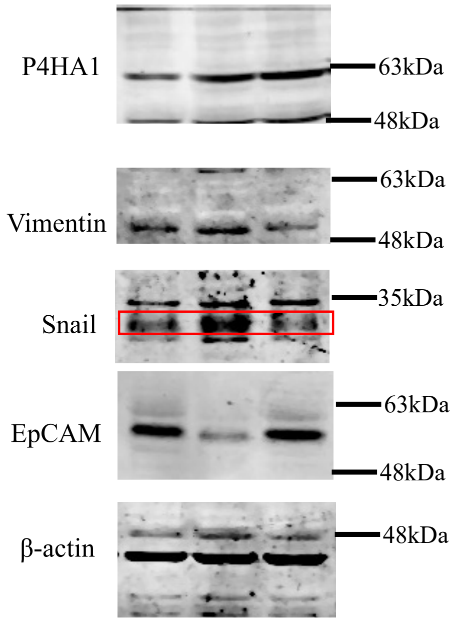

Figure S6I

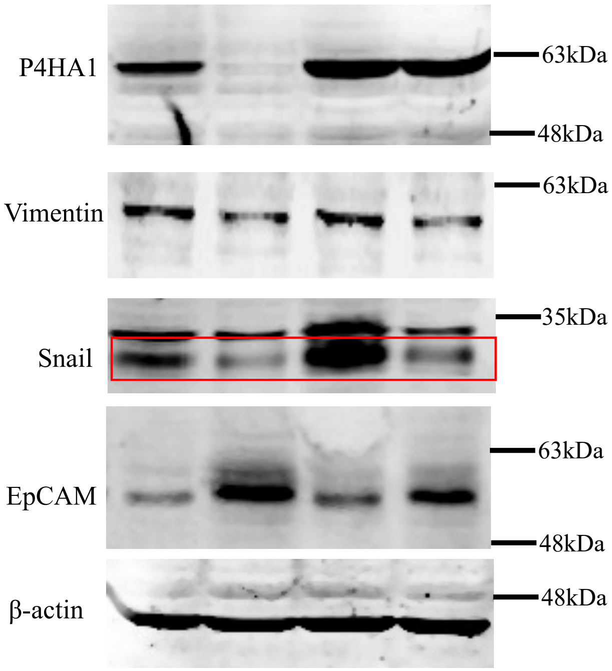

Supplement: Supplementary file 2 [file 41419_2025_7461_MOESM2_ESM.pdf]
