# Supplementary material for: Hypoxia-induced ATF3 escalates breast cancer invasion by increasing collagen deposition via P4HA1
Source: Cell Death Dis. 2025 Feb 27;16(1):142. doi: 10.1038/s41419-025-07461-y (PMC11868403; doi:10.1038/s41419-025-07461-y)
Supplement: Supplementary file 3 [file 41419_2025_7461_MOESM3_ESM.pdf]

Supplementary file 3- Uncropped images of Immunohistochemistry for ATF3 and CAIX in breast cancer patient samples from Figure 1B and Supplementary Figure S2.

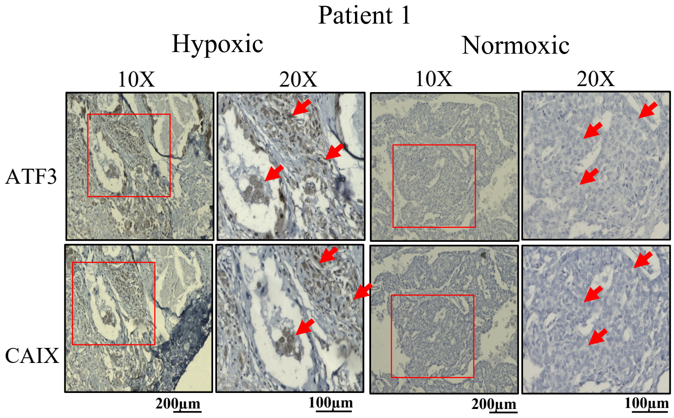

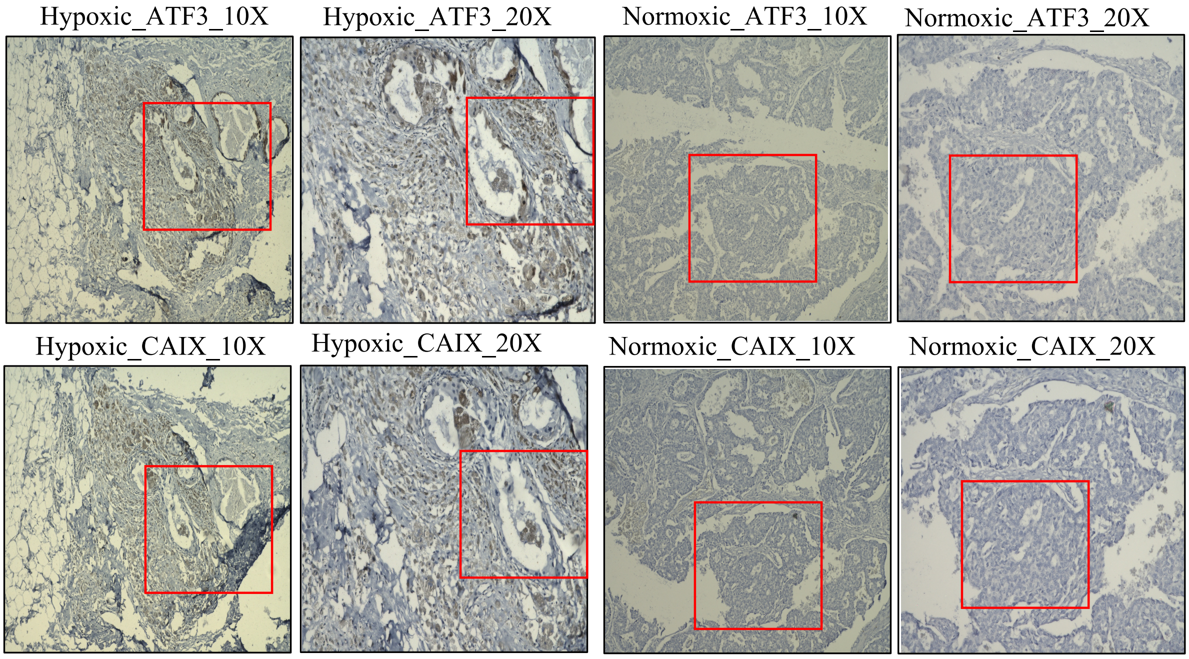

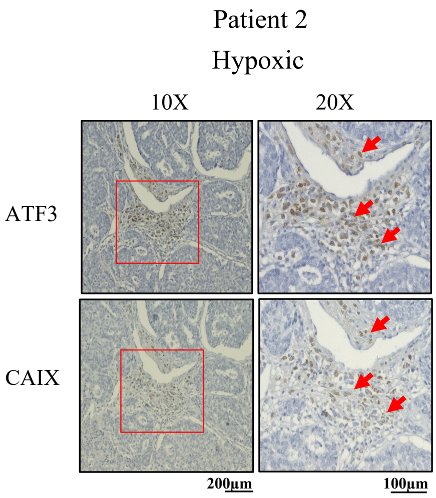
Patient 2
Hypoxic
10X
20X
ATF3
CAIX
200µm
100µm

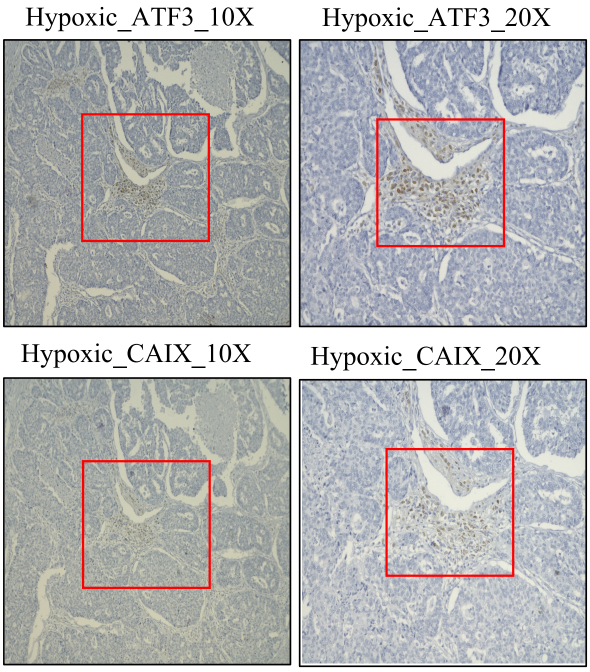
Hypoxic_ATF3_10X
Hypoxic_ATF3_20X
Hypoxic_CAIX_10X
Hypoxic_CAIX_20X

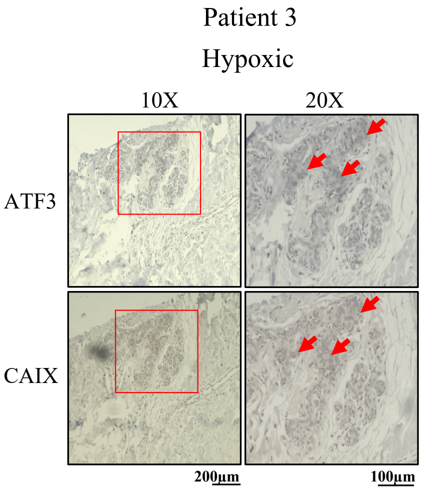
Patient 3
Hypoxic
10X
20X
ATF3
CAIX
200µm
100µm

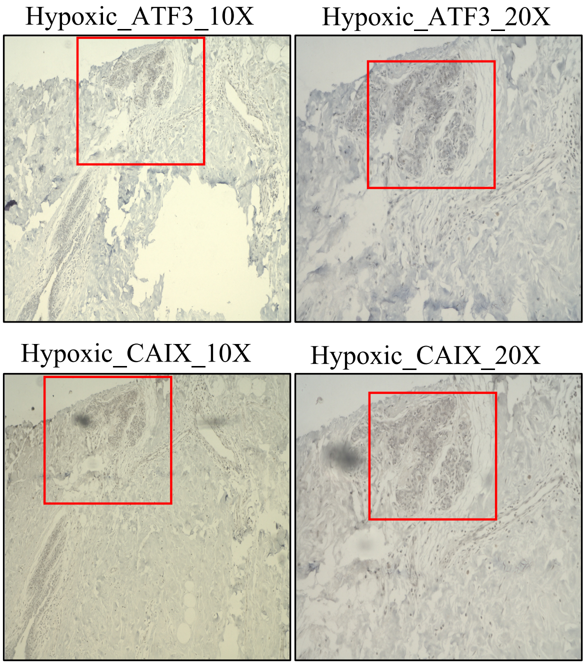
Hypoxic_ATF3_10X
Hypoxic_ATF3_20X
Hypoxic_CAIX_10X
Hypoxic_CAIX_20X

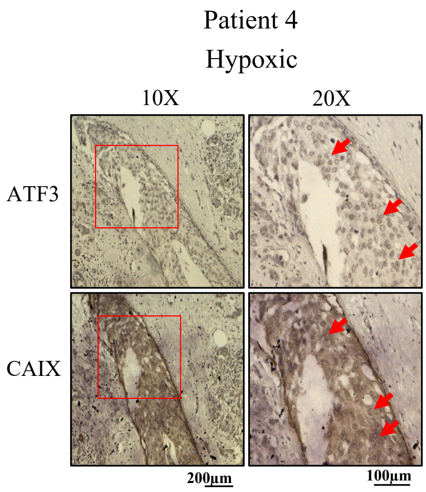
Patient 4
Hypoxic
10X
20X
ATF3
CAIX
200μm
100μm

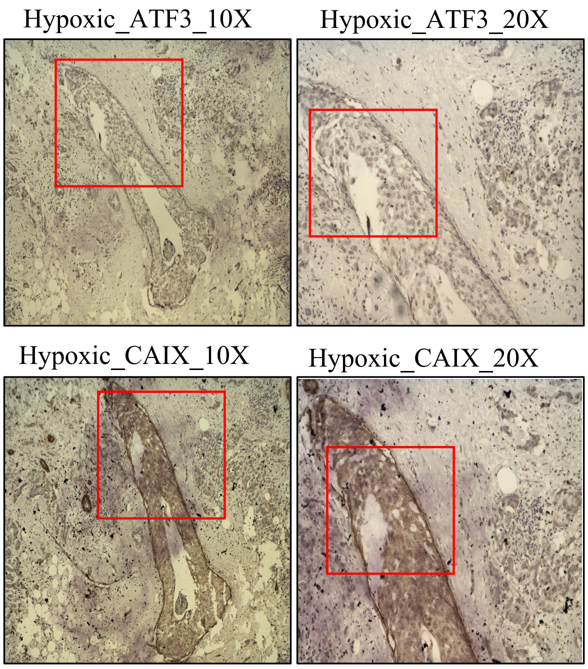
Hypoxic_ATF3_10X
Hypoxic_ATF3_20X
Hypoxic_CAIX_10X
Hypoxic_CAIX_20X

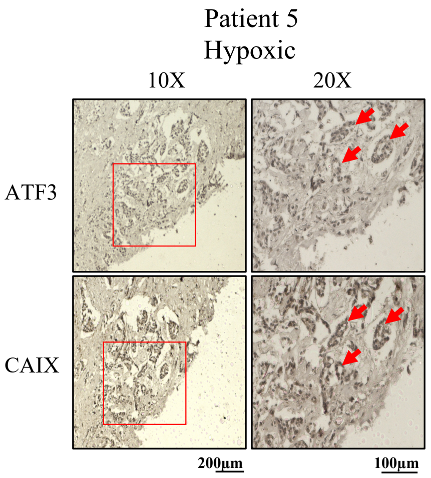
Patient 5
Hypoxic
10X
20X
ATF3
CAIX
200µm
100µm

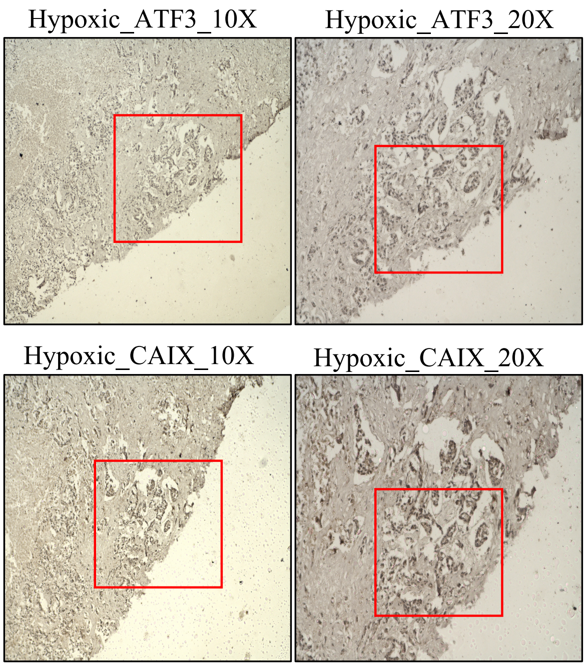
Hypoxic_ATF3_10X
Hypoxic_ATF3_20X
Hypoxic_CAIX_10X
Hypoxic_CAIX_20X

Patient 6
Hypoxic

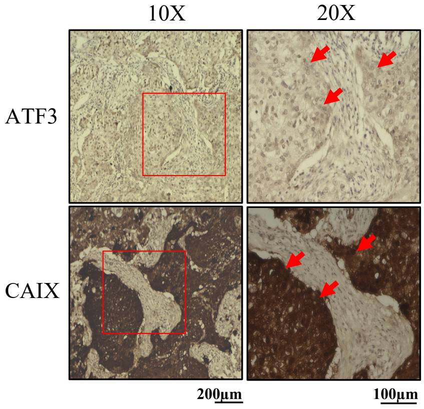

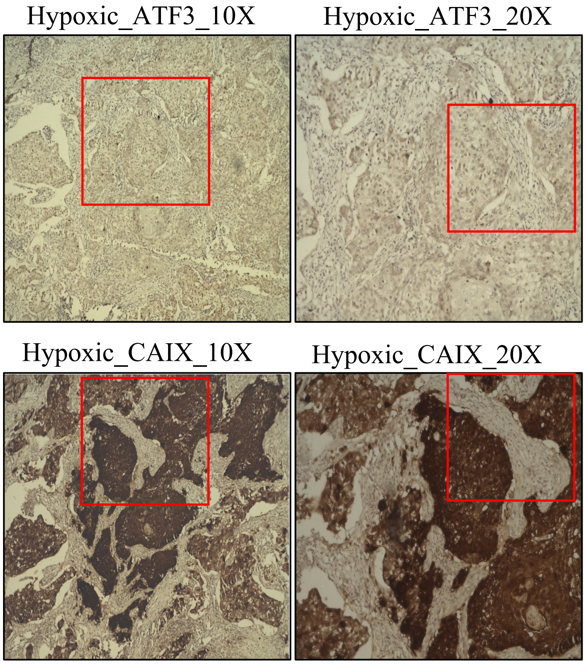

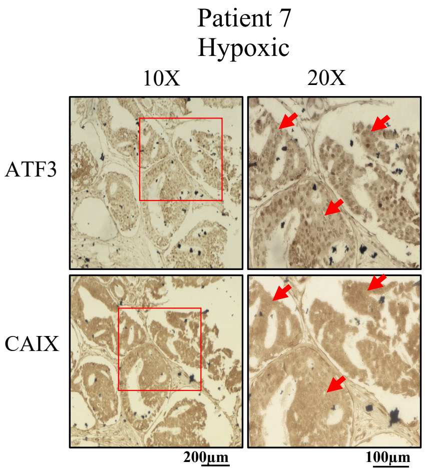
Patient 7
Hypoxic
10X
20X
ATF3
CAIX
200μm
100μm

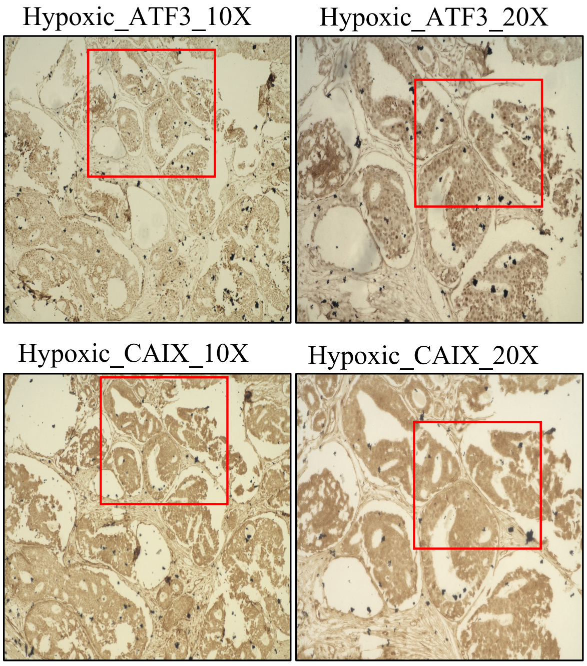
Hypoxic_ATF3_10X
Hypoxic_ATF3_20X
Hypoxic_CAIX_10X
Hypoxic_CAIX_20X

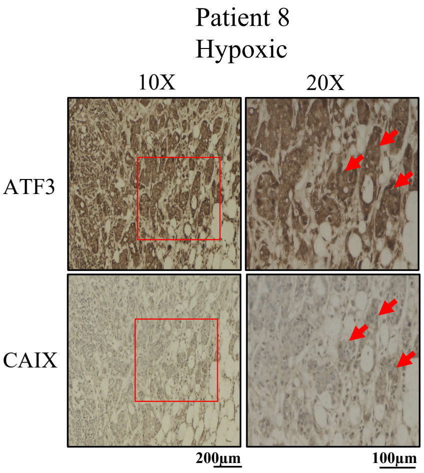
Patient 8
Hypoxic
10X
20X
ATF3
CAIX
200µm
100µm

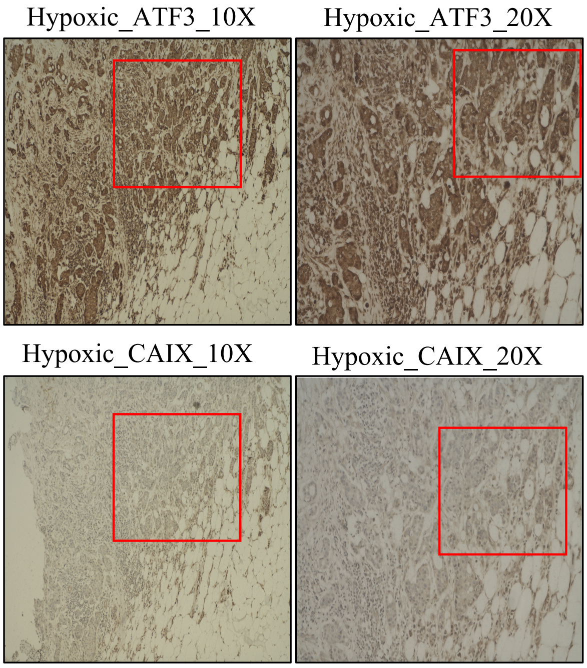
Hypoxic_ATF3_10X
Hypoxic_ATF3_20X
Hypoxic_CAIX_10X
Hypoxic_CAIX_20X

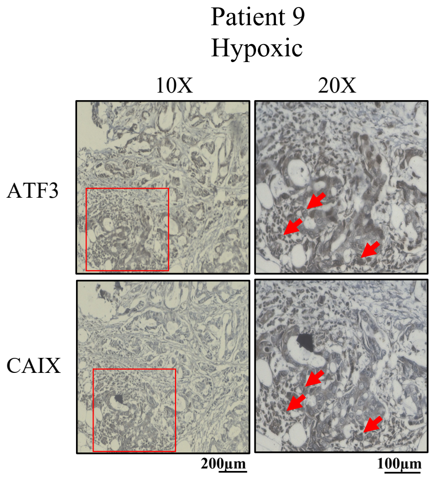
Patient 9
Hypoxic
10X
20X
ATF3
CAIX
200µm
100µm

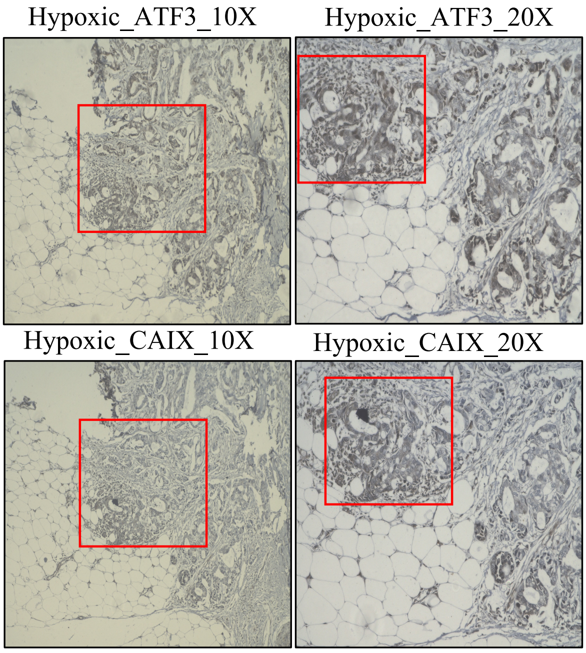
Hypoxic_ATF3_10X
Hypoxic_ATF3_20X
Hypoxic_CAIX_10X
Hypoxic_CAIX_20X

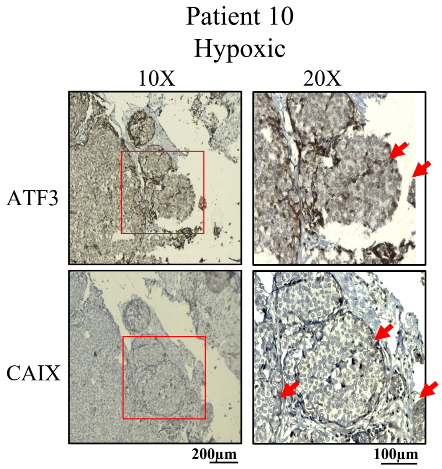
Patient 10
Hypoxic
10X
20X
ATF3
CAIX
200µm
100µm

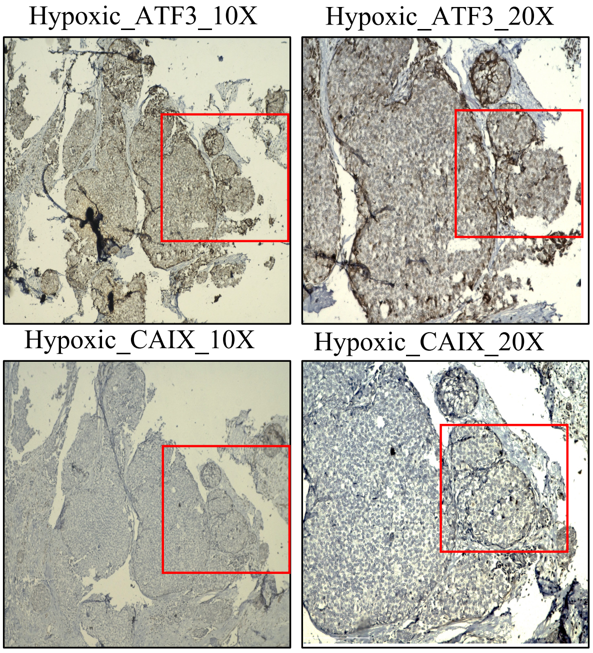
Hypoxic_ATF3_10X
Hypoxic_ATF3_20X
Hypoxic_CAIX_10X
Hypoxic_CAIX_20X

Patient 11

Hypoxic

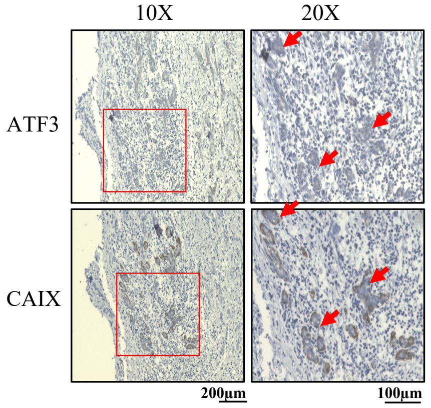

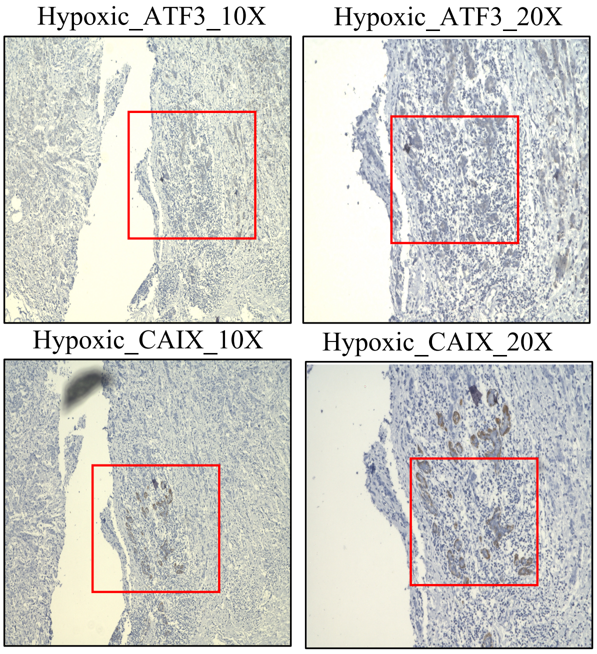

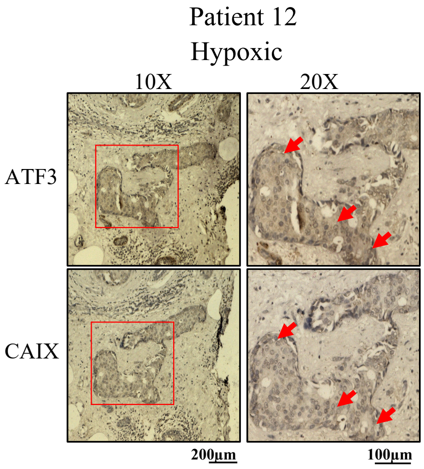
Patient 12
Hypoxic
10X
20X
ATF3
CAIX
200µm
100µm

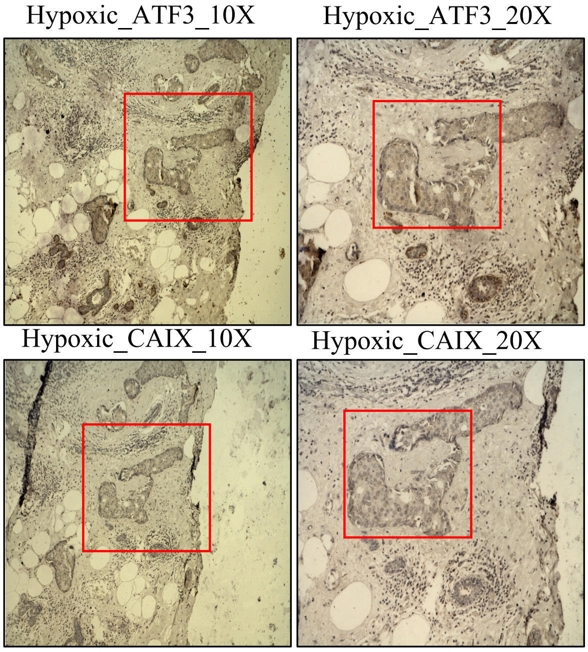
Hypoxic_ATF3_10X
Hypoxic_ATF3_20X
Hypoxic_CAIX_10X
Hypoxic_CAIX_20X

Patient 13
Normoxic

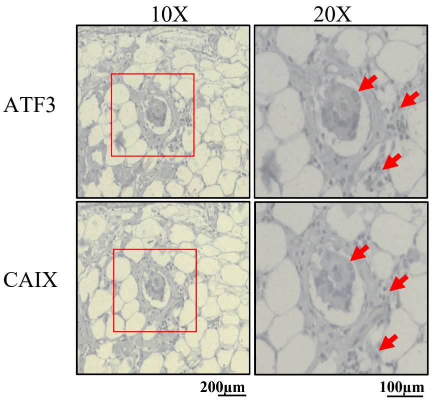

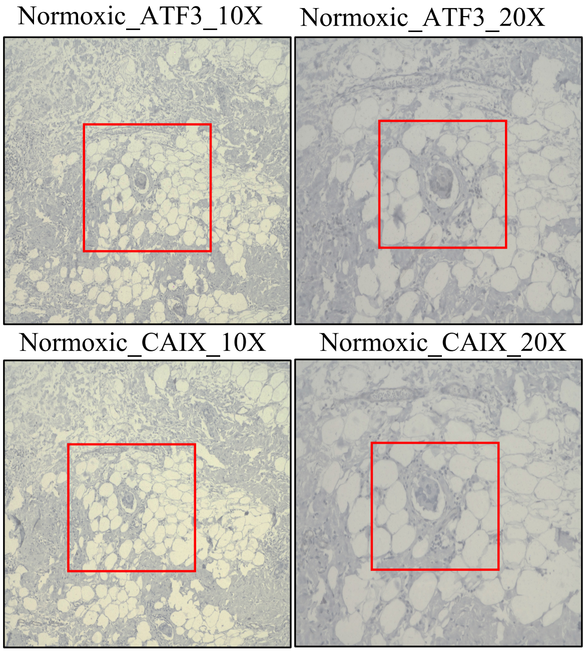

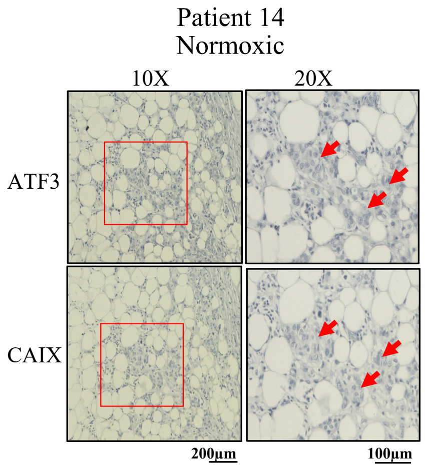
Patient 14
Normoxic
10X
20X
ATF3
CAIX
200µm
100µm

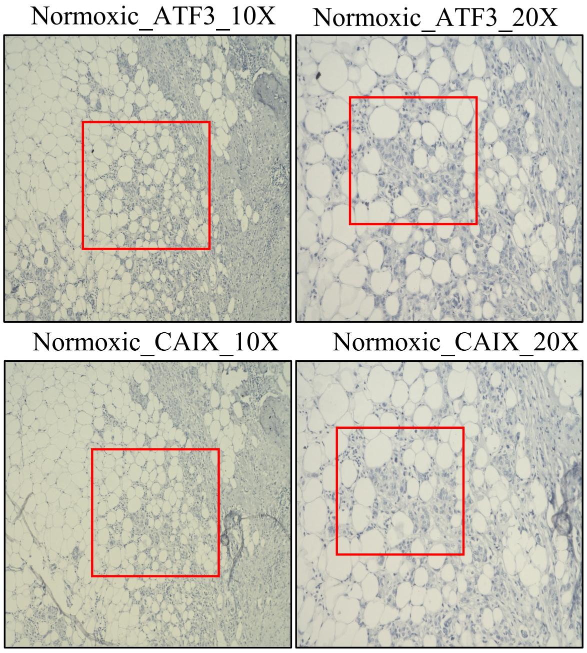
Normoxic_ATF3_10X
Normoxic_ATF3_20X
Normoxic_CAIX_10X
Normoxic_CAIX_20X

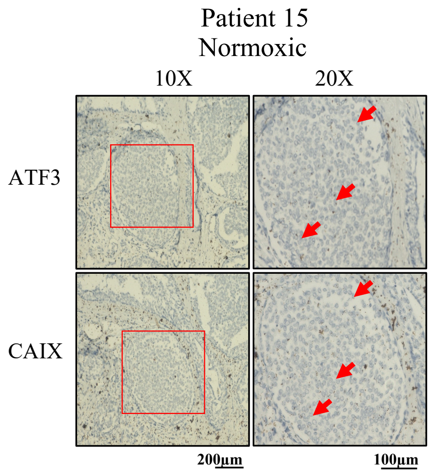
Patient 15
Normoxic
10X
20X
ATF3
CAIX
200μm
100μm

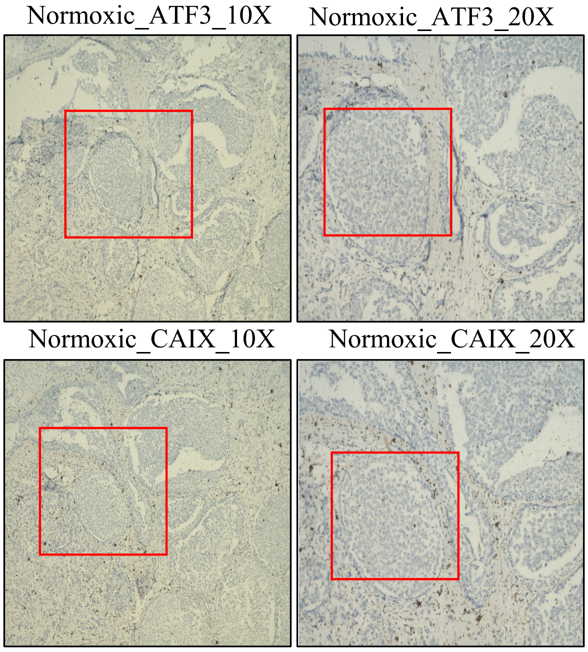
Normoxic_ATF3_10X
Normoxic_ATF3_20X
Normoxic_CAIX_10X
Normoxic_CAIX_20X

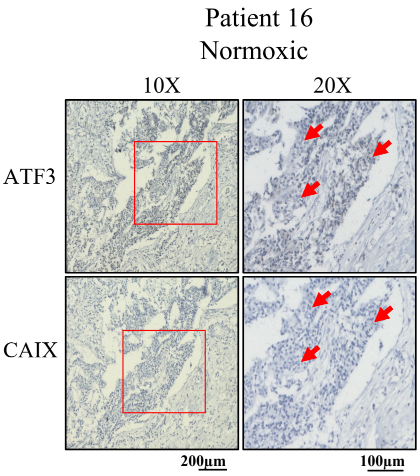
Patient 16
Normoxic
10X
20X
ATF3
CAIX
200µm
100µm

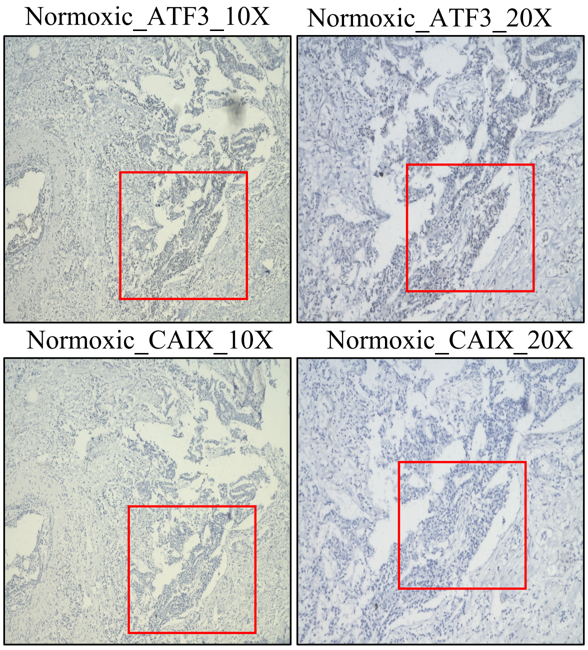
Normoxic_ATF3_10X
Normoxic_ATF3_20X
Normoxic_CAIX_10X
Normoxic_CAIX_20X
